# Early Divergent Strains of *Yersinia pestis* in Eurasia 5,000 Years Ago

**DOI:** 10.1016/j.cell.2015.10.009

**Published:** 2015-10-22

**Authors:** Simon Rasmussen, Morten Erik Allentoft, Kasper Nielsen, Ludovic Orlando, Martin Sikora, Karl-Göran Sjögren, Anders Gorm Pedersen, Mikkel Schubert, Alex Van Dam, Christian Moliin Outzen Kapel, Henrik Bjørn Nielsen, Søren Brunak, Pavel Avetisyan, Andrey Epimakhov, Mikhail Viktorovich Khalyapin, Artak Gnuni, Aivar Kriiska, Irena Lasak, Mait Metspalu, Vyacheslav Moiseyev, Andrei Gromov, Dalia Pokutta, Lehti Saag, Liivi Varul, Levon Yepiskoposyan, Thomas Sicheritz-Pontén, Robert A. Foley, Marta Mirazón Lahr, Rasmus Nielsen, Kristian Kristiansen, Eske Willerslev

**Affiliations:** 1 Center for Biological Sequence Analysis, Department of Systems Biology, Technical University of Denmark, Kemitorvet, Building 208, 2800 Kongens Lyngby, Denmark; 2 Centre for GeoGenetics, Natural History Museum of Denmark, University of Copenhagen, Øster Voldgade 5–7, 1350 Copenhagen, Denmark; 3 Department of Historical Studies, University of Gothenburg, 405 30 Gothenburg, Sweden; 4 Section for Organismal Biology, Department of Plant and Environmental Sciences, University of Copenhagen, Thorvaldsensvej 40, 1871 Frederiksberg C, Denmark; 5 Novo Nordisk Foundation Center for Protein Research, University of Copenhagen, 2200 Copenhagen, Denmark; 6 Division of Armenology and Social Sciences, Institute of Archaeology and Ethnography, National Academy of Sciences, 0025 Yerevan, Republic of Armenia; 7 Institute of History and Archaeology RAS (South Ural Department), South Ural State University, 454080 Chelyabinsk, Russia; 8 Orenburg Museum of Fine Arts, 460000 Orenburg, Russia; 9 Department of Archaeology and Ethnography, Yerevan State University, 0025 Yerevan, Republic of Armenia; 10 Department of Archaeology, University of Tartu, 51003 Tartu, Estonia; 11 Institute of Archaeology, University of Wrocław, 50-139 Wrocław, Poland; 12 Department of Evolutionary Biology, Estonian Biocentre and University of Tartu, 51010 Tartu, Estonia; 13 Peter the Great Museum of Anthropology and Ethnography (Kunstkamera) RAS, 199034 St. Petersburg, Russia; 14 Laboratory of Ethnogenomics, Institute of Molecular Biology, National Academy of Sciences, 0014 Yerevan, Armenia; 15 Leverhulme Centre for Human Evolutionary Studies, Department of Archaeology and Anthropology, University of Cambridge, Cambridge CB2 1QH, UK; 16 Center for Theoretical Evolutionary Genetics, University of California, Berkeley, California 94720-3140, USA; 17 Department of Zoology, University of Cambridge, Downing Street, Cambridge CB2 3EJ, UK

## Abstract

The bacteria *Yersinia pestis* is the etiological agent of plague and has caused human pandemics with millions of deaths in historic times. How and when it originated remains contentious. Here, we report the oldest direct evidence of *Yersinia pestis* identified by ancient DNA in human teeth from Asia and Europe dating from 2,800 to 5,000 years ago. By sequencing the genomes, we find that these ancient plague strains are basal to all known *Yersinia pestis*. We find the origins of the *Yersinia pestis* lineage to be at least two times older than previous estimates. We also identify a temporal sequence of genetic changes that lead to increased virulence and the emergence of the bubonic plague. Our results show that plague infection was endemic in the human populations of Eurasia at least 3,000 years before any historical recordings of pandemics.

## Highlights

• *Yersinia pestis* was common across Eurasia in the Bronze Age
• The most recent common ancestor of all *Y. pestis* was 5,783 years ago
• The *ymt* gene was acquired before 951 cal BC, giving rise to transmission via fleas
• Bronze Age *Y. pestis* was not capable of causing bubonic plague

## Graphical Abstract

**Figure:**
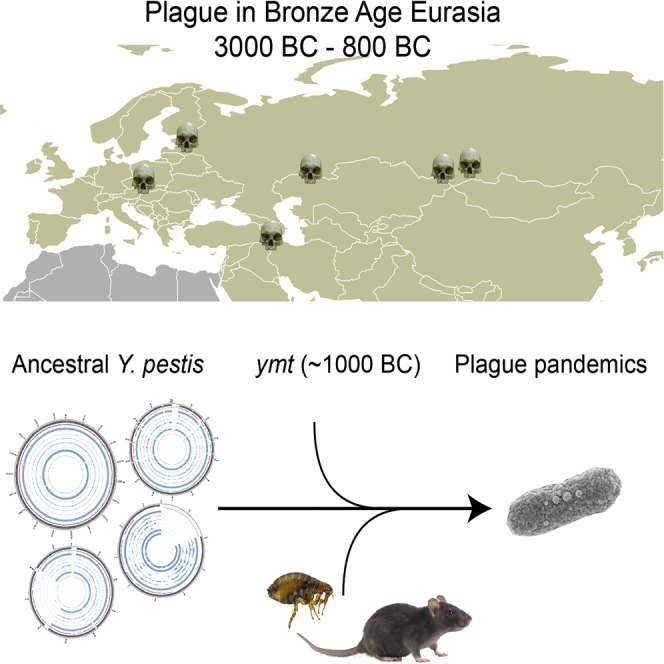

## Teaser

The plague-causing bacteria *Yersinia pesti*s infected humans in Bronze Age Eurasia, three millennia earlier than any historical records of plague, but only acquired the genetic changes making it a highly virulent, flea-borne bubonic strain ∼3,000 years ago.

## Introduction

Plague is caused by the bacteria *Yersinia pestis* and is being directly transmitted through human-to-human contact (pneumonic plague) or via fleas as a common vector (bubonic or septicemic plague) (Treille and Yersin, 1894). Three historic human plague pandemics have been documented: (1) the First Pandemic, which started with the Plague of Justinian (541–544 AD), but continued intermittently until ∼750 AD; (2) the Second Pandemic, which began with the Black Death in Europe (1347–1351 AD) and included successive waves, such as the Great Plague (1665–1666 AD), until the 18^th^ century; (3) the Third Pandemic, which emerged in China in the 1850s and erupted there in a major epidemic in 1894 before spreading across the world as a series of epidemics until the middle of the 20^th^ century (Bos et al., 2011, Cui et al., 2013, Drancourt et al., 1998, Harbeck et al., 2013, Parkhill et al., 2001, Perry and Fetherston, 1997, Wagner et al., 2014). Earlier outbreaks such as the Plague of Athens (430–427 BC) and the Antonine Plague (165–180 AD) may also have occurred, but there is no direct evidence that allows confident attribution to *Y. pestis* (Drancourt and Raoult, 2002, McNeill, 1976).

The consequences of the plague pandemics have been well-documented and the demographic impacts were dramatic (Little et al., 2007). The Black Death alone is estimated to have killed 30%–50% of the European population. Economic and political collapses have also been in part attributed to the devastating effects of the plague. The Plague of Justinian is thought to have played a major role in weakening the Byzantine Empire, and the earlier putative plagues have been associated with the decline of Classical Greece and likely undermined the strength of the Roman army.

Molecular clock estimates have suggested that *Y. pestis* diversified from the more prevalent and environmental stress-tolerant, but less pathogenic, enteric bacterium *Y. pseudotuberculosis* between 2,600 and 28,000 years ago (Achtman et al., 1999, Achtman et al., 2004, Cui et al., 2013, Wagner et al., 2014). However, humans may potentially have been exposed to *Y. pestis* for much longer than the historical record suggests, though direct molecular evidence for *Y. pestis* has not been obtained from skeletal material older than 1,500 years (Bos et al., 2011, Wagner et al., 2014). The most basal strains of *Y. pestis* (0.PE7 clade) recorded to date were isolated from the Qinghai-Tibet Plateau in China in 1961–1962 (Cui et al., 2013).

We investigated the origin of *Y. pestis* by sequencing ancient bacterial genomes from the teeth of Bronze Age humans across Europe and Asia. Our findings suggest that the virulent, flea-borne *Y. pestis* strain that caused the historic bubonic plague pandemics evolved from a less pathogenic *Y. pestis* lineage infecting human populations long before recorded evidence of plague outbreaks.

## Results

### Identification of *Yersinia pestis* in Bronze Age Eurasian Individuals

**Figure 1:**
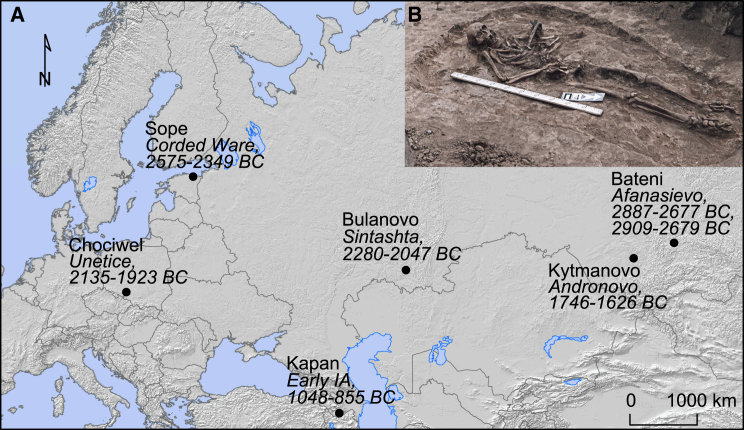
Archaeological Sites of Bronze Age *Yersinia pestis* (A) Map of Eurasia indicating the position, radiocarbon dated ages and associated cultures of the samples in which *Y. pestis* were identified. Dates are given as 95% confidence interval calendar BC years. IA: Iron Age. (B) Burial four from Bulanovo site. Picture by Mikhail V. Khalyapin. See also Table S1.

**Figure 2:**
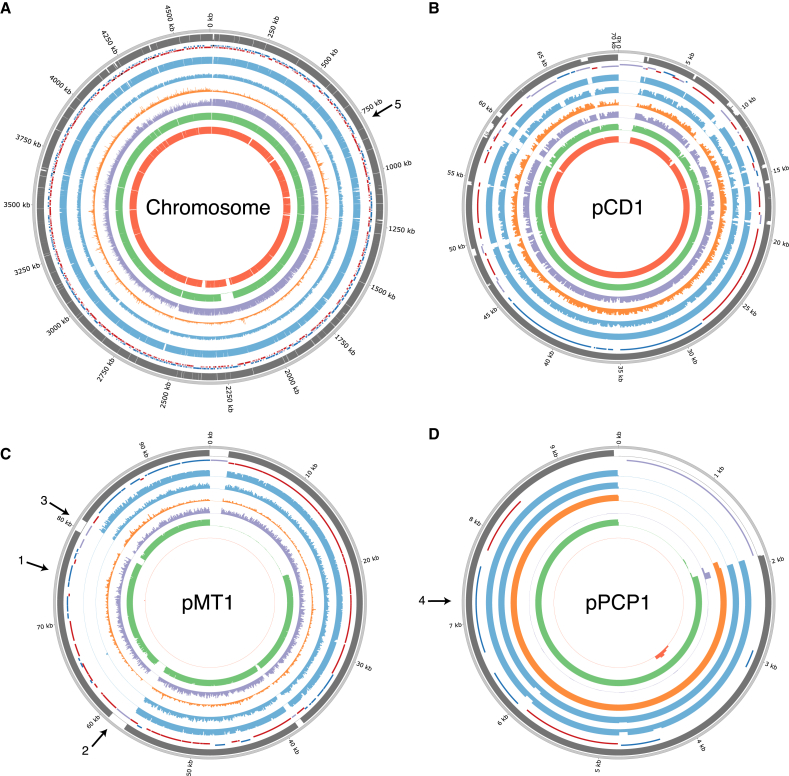
*Y. pestis* Depth of Coverage Plots (A–D) Depth of coverage plots for (A) CO92 chromosome, (B) pCD1, (C) pMT1, (D) pPCP1. Outer ring: Mappability (gray), genes (RNA: black, transposon: purple, positive strand: blue, negative strand: red), RISE505 (blue), RISE509 (blue), Justinian plague (orange), Black Death plague (purple), modern *Y. pestis* D1982001 (green), *Y. pseudotuberculosis* IP32881 (red) sample. The modern *Y. pestis* and *Y. pseudotuberculosis* samples are included for reference. The histograms show sequence depth in 1 kb windows for the chromosome and 100 bp windows for the plasmids with a max of 20X depth for each ring. Arrow 1: *ymt* gene, arrow 2: transposon at start of missing region on pMT1, arrow 3: transposon at end of missing region on pMT1, arrow 4: *pla* gene, arrow 5: missing flagellin region on chromosome. The plots were generated using Circos (Krzywinski et al., 2009). See also Tables S2, S3 and S8.

We screened c. 89 billion raw DNA sequence reads obtained from teeth of 101 Bronze Age individuals from Europe and Asia (Allentoft et al., 2015) and found that seven individuals carried sequences resembling *Y. pestis* (Figure 1, Table S1, Supplemental Experimental Procedures). Further sequencing allowed us to assemble the *Y. pestis* genomes to an average depth of 0.14–29.5X, with 12%–95% of the positions in the genome covered at least once (Table 1, Table S2, S3, and S4). We also recovered the sequences of the three plasmids pCD1, pMT1, and pPCP1 (0.12 to 50.3X in average depth) the latter two of which are crucial for distinguishing *Y. pestis* from its highly similar ancestor *Y. pseudotuberculosis* (Table 1, Figure 2, Table S3) (Bercovier et al., 1980, Chain et al., 2004, Parkhill et al., 2001). The host individuals from which *Y. pestis* was recovered belong to Eurasian Late Neolithic and Bronze Age cultures (Allentoft et al., 2015), represented by the Afanasievo culture in Altai, Siberia (2782 cal BC, 2794 cal BC, n = 2), the Corded Ware culture in Estonia (2462 cal BC, n = 1), the Sintashta culture in Russia (2163 cal BC, n = 1), the Unetice culture in Poland (2029 cal BC, n = 1), the Andronovo culture in Altai, Siberia (1686 cal BC, n = 1), and an early Iron Age individual from Armenia (951 cal BC, n = 1) (Table S1).

### Authentication of *Yersinia pestis* Ancient DNA

**Figure 3:**
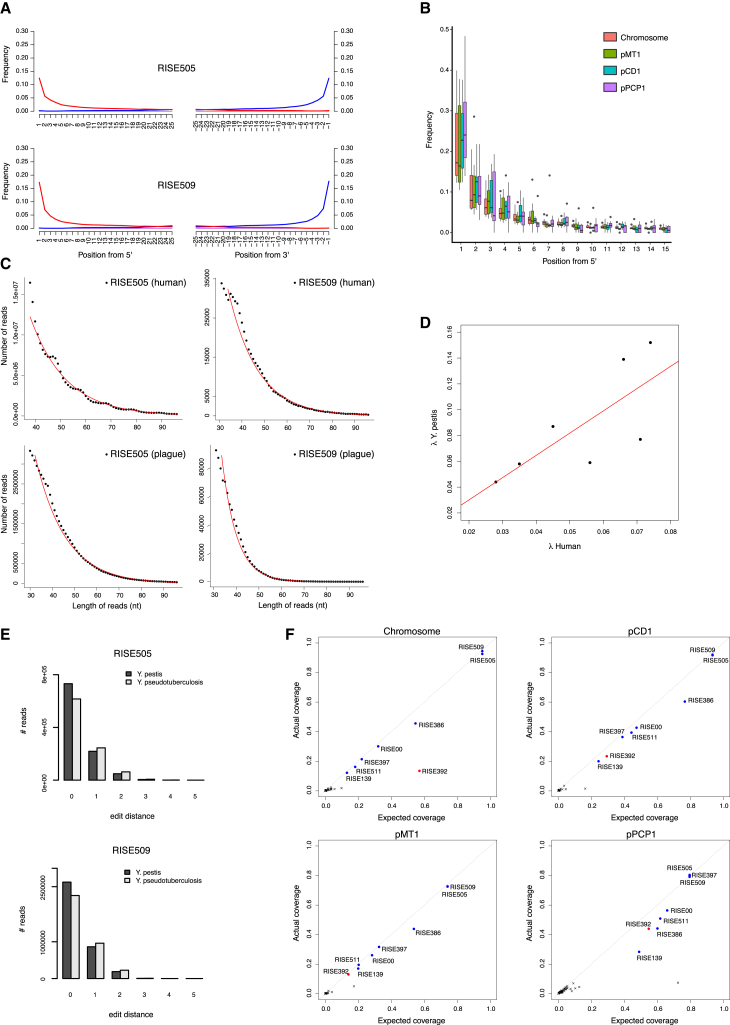
Authenticity of *Y. pestis* DNA (A) DNA damage patterns for RISE505 and RISE509. The frequencies of all possible mismatches observed between the *Y. pestis* CO92 chromosome and the reads are reported in gray as a function of distance from 5′ (left panel, first 25 nucleotides sequenced) and distance to 3′ (right panel, last 25 nucleotides). The typical DNA damage mutations C>T (5′) and G>A (3′) are reported in red and blue, respectively. (B) Ancient DNA damage patterns (n = 7) of the reads aligned to the CO92 chromosome and the *Y. pestis* associated plasmids pMT1, pCD1 and pPCP1. The boxplots show the distribution of C-T damage in the 5′ of the reads. The lower and upper hinges of the boxes correspond to the 25th and 75th percentiles, the whiskers represent the 1.5 inter-quartile range (IQR) extending from the hinges, and the dots represent outliers from these. (C) DNA fragment length distributions from RISE505 and RISE509 samples representing both the *Y. pestis* DNA and the DNA of the human host. The declining part of the distributions is fitted to an exponential model (red). (D) Linear correlation (red) between the decay constant in the DNA of the human host and the associated *Y. pestis* DNA extracted from the same individual (R^2^ = 0.55, p = 0.055). The decay constant (λ) describes the damage fraction (i.e., the fraction of broken bonds on the DNA strand). (E) Distribution of edit distance of high quality reads from RISE505 and RISE509 samples mapped to either *Y. pestis* (dark gray) or *Y. pseudotuberculosis* (light gray) reference genomes. The reads have a higher affinity to *Y. pestis* than to *Y. pseudotuberculosis*. (F) Plots of actual coverage versus expected coverage for the 101 screened samples. Expected coverage was computed taking into account read length distributions, mappable fractions of reference sequences, and the deletions in pMT1 for some of the samples. Samples assumed to contain *Y. pestis* are shown in blue and RISE392 that is classified as not *Y. pestis* appears is shown in red. See also Figure S1 and S2, Table S3.

**Figure 4:**
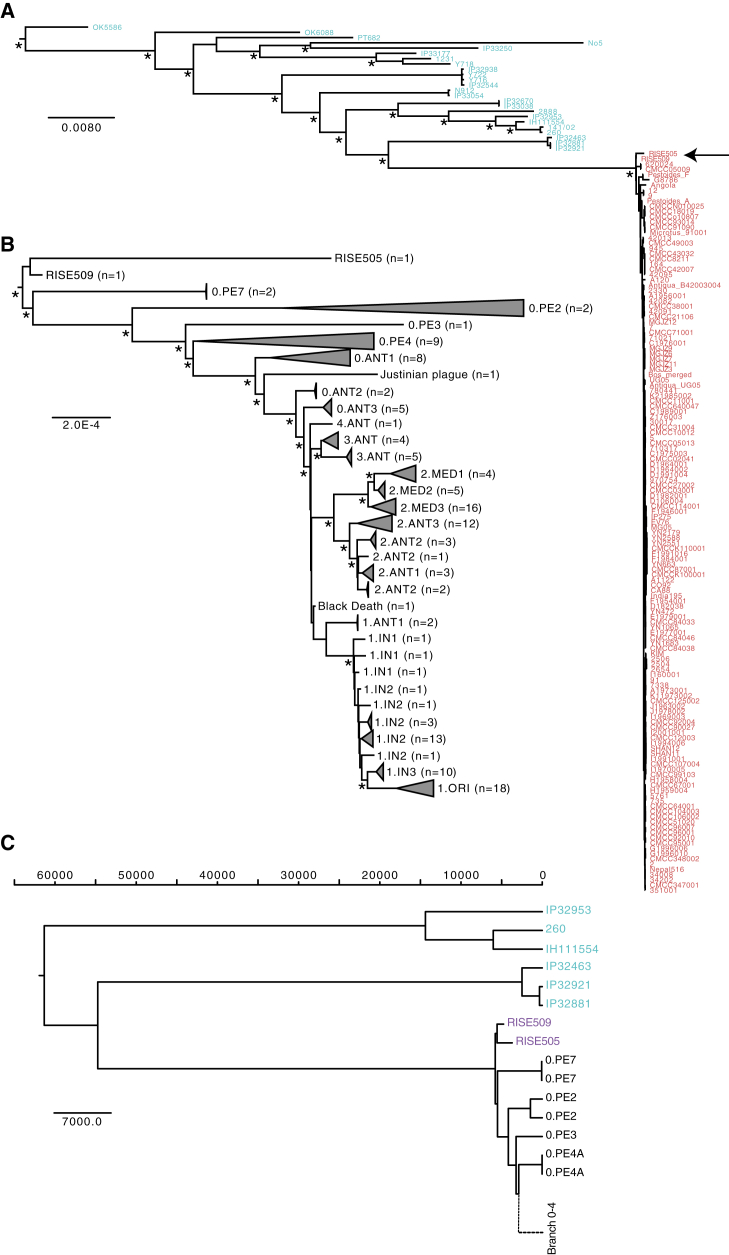
Phylogenetic Reconstructions (A) Maximum Likelihood reconstruction of the phylogeny of *Y. pseudotuberculosis* (blue) and *Y. pestis* (red). The tree is rooted using *Y. similis* (not shown). The full tree including three additional *Y. pseudotuberculosis* strains (O:15 serovar) can be seen in Figure S4. Major branching nodes within *Y. pseudotuberculosis* with > 95% bootstrap support are indicated with an asterisk and branch lengths are given as substitutions per site. (B) Maximum Likelihood reconstruction of the phylogeny in (A) showing only the *Y. pestis* clade. The clades are collapsed by population according to branches and serovars, as given in (Achtman et al., 1999, Achtman et al., 2004, Cui et al., 2013). See Figure S4 for an uncollapsed tree and Table S2 for details on populations. Nodes with more than 95% bootstrap support are indicated with an asterisk and branch lengths are given as substitutions per site. (C) BEAST2 maximum clade credibility tree showing median divergence dates. Branch lengths are given as years before the present (see Divergence estimations in Experimental Procedures). Only the *Y. pseudotuberculosis* (blue), the ancient *Y. pestis* samples (magenta) and the most basal branch 0 strains (black) are shown. For a full tree including all *Y. pestis* see Figure S5. See also Figure S3, S4, and S5 and Table S5.

**Figure S1:**
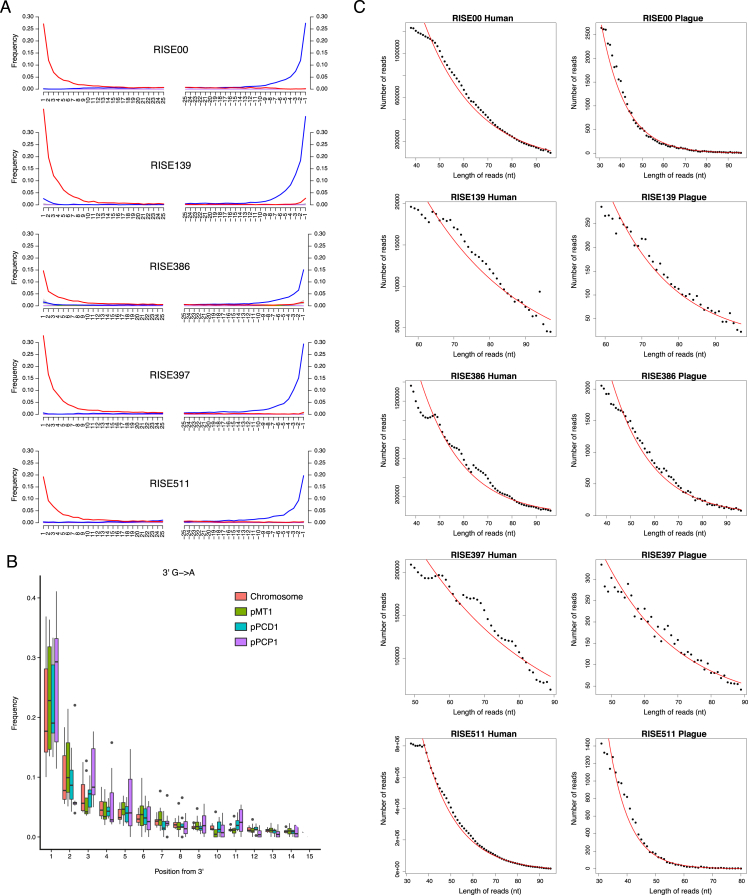
DNA Damage and Decay, Related to Figure 3 (A) DNA damage patterns for the five *Y. pestis* associated samples not shown in Figure 3. The frequencies of all possible mismatches observed between the *Y. pestis* CO92 chromosome and the reads are reported in gray as a function of distance from 5′ (left panel, first 25 nucleotides sequenced) and distance to 3′ (right panel, last 25 nucleotides). The typical DNA damage bases are C>T (5′) and G>A (3′) mutations are reported in red and blue, respectively. (B) Ancient DNA damage patterns of the reads aligned to the CO92 chromosome and the *Y. pestis* associated plasmids pMT1, pCD1 and pPCP1. The boxplots show the distribution of G-A damage in the 3′ of the reads. The distributions are made from the seven *Y. pestis* samples. The lower and upper hinges of the boxes correspond to the 25th and 75th percentiles, the whiskers represent the 1.5 inter-quartile range (IQR) extending from the hinges, and the dots represent outliers from these. (C) DNA fragment length distributions from five *Y. pestis* samples not shown in Figure 3 representing both the *Y. pestis* DNA and the DNA of the human host. The declining part of the distributions is fitted to an exponential model (red).

**Figure S2:**
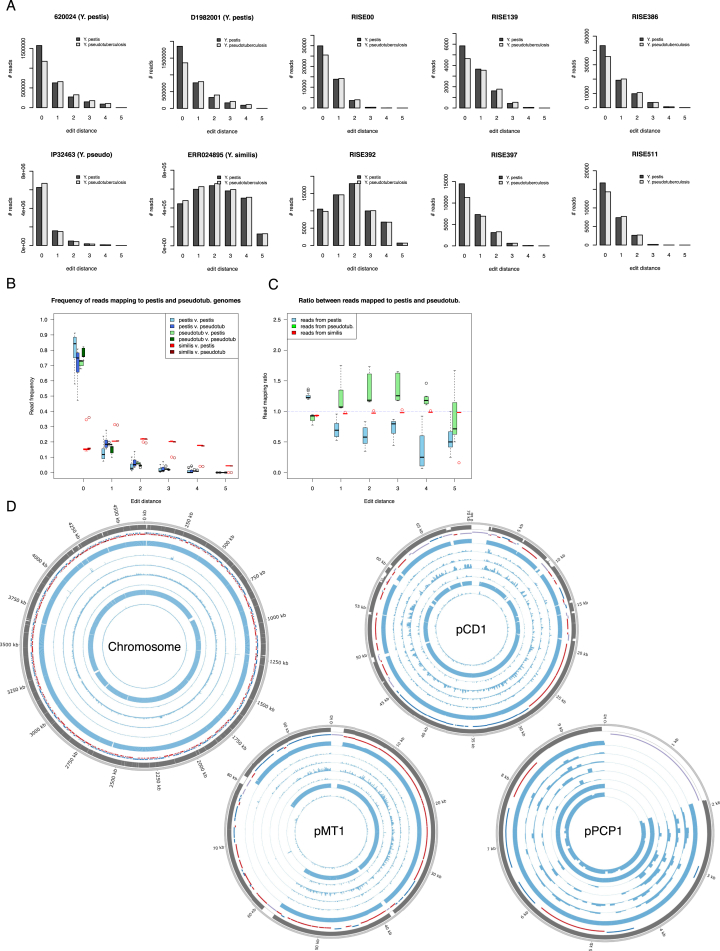
Mapping Affinity, Related to Figure 3 (A) Distribution of edit distance of high quality reads of known origin and the eight *Yersinia* associated samples. The investigated, known reads are from *Y. pestis* 620024 (0.PE7), *Y. pestis* D1982001 (1.IN2), *Y. pseudo* (IP32464) (from the clade closest to *Y. pestis*), and *Y. similis* (which is an outgroup to both *Y. pestis* and *Y. pseudotuberculosis*). For RISE00, RISE139, RISE386, RISE397, RISE505, RISE509 and RISE511 the reads are closer to *Y. pestis* than to *Y. pseudotuberculosis*, and there are far more hits at low edit distances (RISE505 and RISE509 are shown in Figure 3). This is consistent with these reads originating from *Y. pestis*. Reads from the RISE392 sample instead have more hits at higher edit distances and have similar distances to both the *Y. pestis* and *Y. pseudotuberculosis* reference genomes. This suggests that RISE392 is neither *Y. pestis* nor *Y. pseudotuberculosis*, but a more distantly related species. (B) Distribution of the amount of reads mapping to the *Y. pestis* reference genome, at different edit distances. For each of the three investigated species (*Y. pestis* n = 10, *Y. pseudotuberculosis* n = 10, and *Y. similis* n = 5) several different sets of reads were mapped against the reference, and the number of reads matching at different edit distances was counted. For each edit distance the distribution of reads for each species is shown in the form of a boxplot. The lower and upper hinges of the boxes correspond to the 25th and 75th percentiles, the whiskers represent the 1.5 inter-quartile range (IQR) extending from the hinges, and the dots represent outliers from these. (C) Ratio between the number of reads mapping to *Y. pestis* and the number of reads mapping to *Y. pseudotuberculosis*, for different edit distances, for three investigated species. Input data as in B. For each sample the ratio between the number of reads matching *Y. pestis*, and the number of reads matching *Y. pseudotuberculosis* was calculated, and the distribution of these ratios then shown in the form of a boxplot for each edit distance. These features were used to predict the taxonomy of unknown samples. The lower and upper hinges of the boxes correspond to the 25th and 75th percentiles, the whiskers represent the 1.5 inter-quartile range (IQR) extending from the hinges, and the dots represent outliers from these. (D) Depth of coverage plots for the seven ancient *Y. pestis* samples mapped to the CO92 chromosome, pCD1, pMT1 and pPCP1. The RISE samples are ordered according to age where the oldest sample is the outermost histogram. Outer ring: Mappability (gray), genes (RNA: black, transposon: purple, positive strand: blue, negative strand: red), RISE509, RISE511, RISE00, RISE386, RISE139, RISE505 and RISE397 (blue). Depth histograms show sequence depth in 1 kb windows for the chromosome and 100 bp for the plasmids with a max of 5X depth for each ring. The plots were generated using Circos.

Besides applying standard precautions for working with ancient DNA (Willerslev and Cooper, 2005), the authenticity of our findings are supported by the following observations: (1) The *Y. pestis* sequences were identified in significant amounts in shotgun data from eight of 101 samples, showing that this finding is not due to a ubiquitous contaminant in our lab or in the reagents. Indeed, further analysis showed that one of these eight was most likely not *Y. pestis*. We also sequenced all negative DNA extraction controls and found no signs of *Y. pestis* DNA in these (Table S3). (2) Consistent with an ancient origin, the *Y. pestis* reads were highly fragmented, with average read lengths of 43–65 bp (Table S3) and also displayed clear signs of C-T deamination damage at the 5′ termini typical of ancient DNA (Figure 3, Figure S1). Because the plasmids are central for discriminating between *Y. pestis* and *Y. pseudotuberculosis*, we tested separately for DNA damage patterns for the chromosome and for each of the plasmids. For the seven samples, we observe similar patterns of DNA damage for chromosome and plasmid sequences (Figure 3, Figure S1). (3) We observe correlated DNA degradation patterns when comparing DNA degradation in the *Y. pestis* sequences and the human sequences from the host individual. Given that DNA decay can be described as a rate process (Allentoft et al., 2012), this suggests that the DNA molecules of the pathogen and the human host have a similar age (Figure 3, Figure S1, Table S3 and Supplemental Experimental Procedures). (4) Because of the high sequence similarity between *Y. pestis* and *Y. pseudotuberculosis*, we mapped all reads both to the *Y. pestis* CO92 and to the *Y. pseudotuberculosis* IP32953 reference genomes (Chain et al., 2004). Consistent with being *Y. pestis*, the seven investigated samples displayed more reads matching perfectly (edit distance = 0) toward *Y. pestis* (Figure 3, Figure S2). One sample (RISE392) was most likely not *Y. pestis* based on this criterion. (5) A naive Bayesian classifier trained on known genomes predicts the seven samples to be *Y. pestis* with 100% posterior probability, while RISE392 is predicted to have 0% probability of being *Y. pestis* (Figure S2, Table S3). (6) If the DNA was from other organisms than *Y. pestis*, we would expect the reads to be more frequently associated with either highly conserved or low-complexity regions. However, we find the reads to be distributed across the entire genome (Figure S2), and comparison of actual coverage versus the coverage that would be expected from read length distributions and mappability of the reference sequences are also in agreement for the seven samples (Figure 3). (7) In a maximum likelihood phylogeny, the recovered *Y. pestis* genomic sequences of RISE505 and RISE509 are clearly within the *Y. pestis* clade and basal to all contemporary *Y. pestis* strains (Figure 4) (see below).

### The Phylogenetic Position of the Bronze Age *Yersinia pestis* Strains

**Figure S3:**
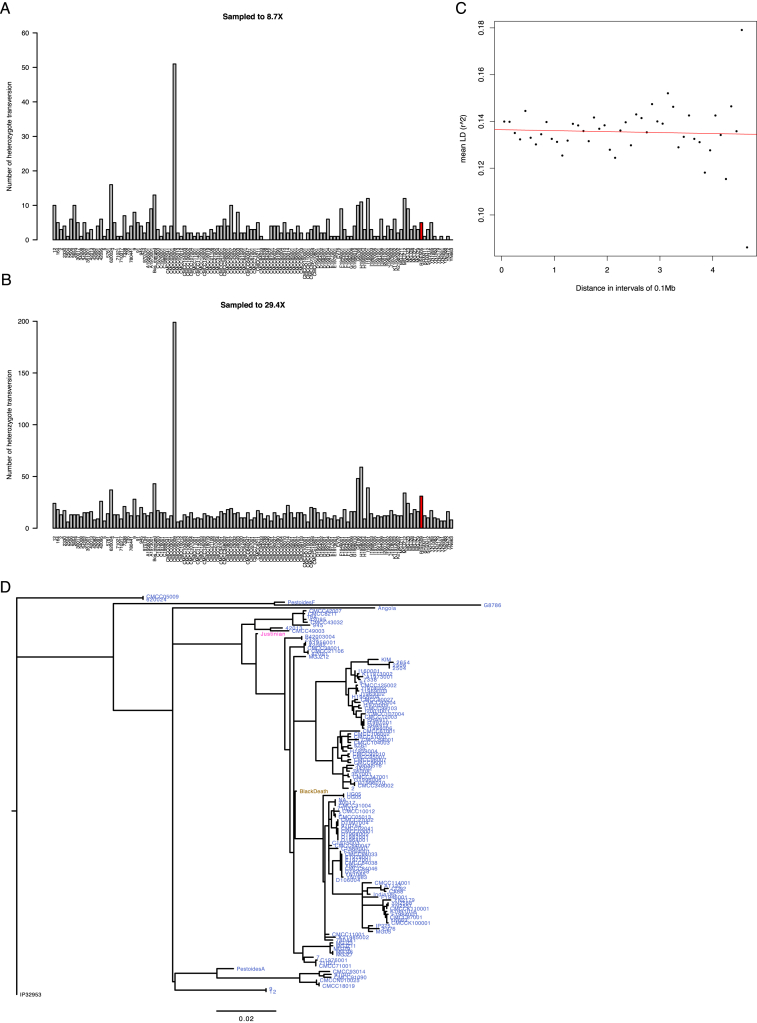
Phylogenetics, Related to Figure 4 (A and B) Heterozygosity estimates of RISE505 (A) and RISE509 (B), the respective ancient *Y. pestis* samples are shown in red. All samples were downsampled to the same depth as either RISE505 or RISE509 and the number of heterozygote transversions determined (y axis). (C) Linkage Disequilibrium (LD) determined from 141 *Y. pestis* strains in 0.1Mb intervals across the *Y. pestis* CO92 chromosome. There is no decay in LD across the genome which means that there are no recombination and the phylogenetic tree can be averaged across the individual genes. (D) Maximum Likelihood tree generated using RAxML and the 2,298 phylogenetic informative sites described by Morelli et al. (2010) and Cui et al. (2013). The strains are colored by species with *Y. pseudotuberculosis* IP32953 being black and *Y. pestis* blue. The Justinian plague sample and the Black Death samples are colored in magenta and brown, respectively. Branch lengths are substitutions per site.

**Figure S4:**
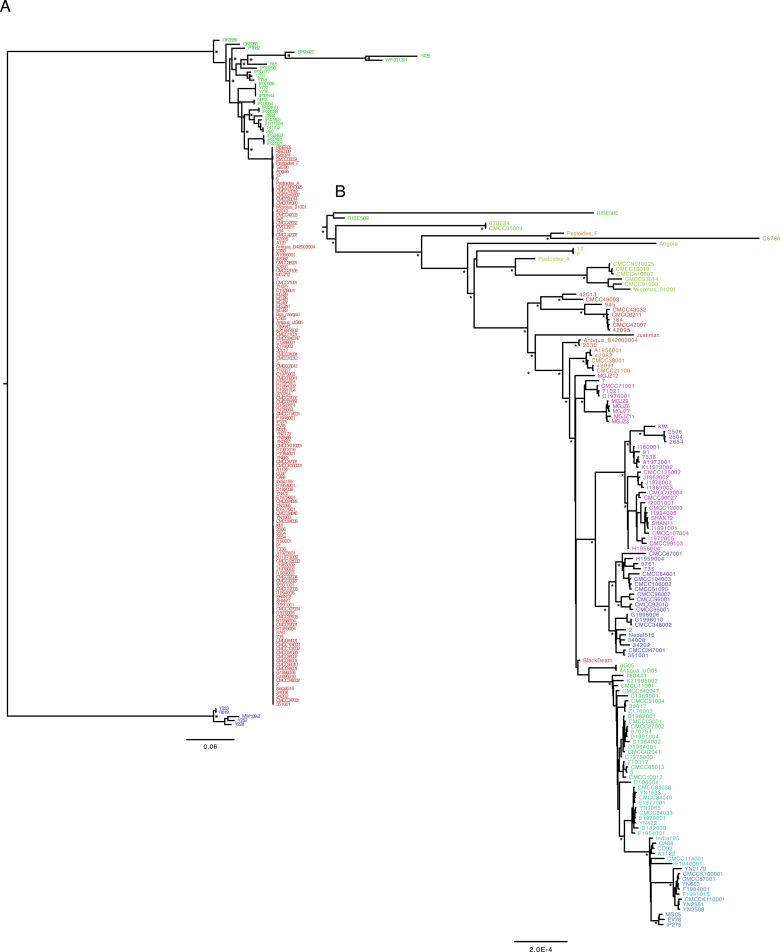
Phylogenetic Trees, Related to Figure 4 (A) Maximum Likelihood phylogeny of all strains used in the analysis. *Y. similis* (blue), *Y. pseudotuberculosis* (green) and *Y. pestis* (red). The strains that were excluded from the phylogeny in Figure 4A: SP93422, Y428 and WP-931201. Major branch nodes with bootstrap support > 95% are indicated with an asterisk. Branch lengths are substitutions per site. (B) Maximum Likelihood tree of the *Y. pestis* clade only. The tree is the un-collapsed version of the tree shown in Figure 4B. Nodes marked with an asterisk have > 95% bootstrap support, not all internal nodes are marked with bootstrap values. Strain names are colored according to the population nomenclature in Table S2. Branch lengths are substitutions per site.

To determine the phylogenetic positions of the two high coverage ancient *Y. pestis* strains, RISE505 (Andronovo culture 1686 cal BC, 8.7X) and RISE509 (Afanasievo culture, 2746 cal BC, 29.7X), we mapped the reads, together with reads from strains of *Yersinia similis* (n = 5), *Y. pseudotuberculosis* (n = 25), and *Y. pestis* (n = 139), to the *Y. pseudotuberculosis* reference genome (IP32953). Only high confidence positions were extracted. To assess whether the individuals were infected with multiple strains of *Y. pestis* we investigated the genotype heterozygosity levels of the ancient genomes and found no indications of mixed infection (Figure S3). There was no decay in Linkage Disequilibrium (LD) across the chromosome (Figure S3), indicating no detectable recombination among strains. We therefore used RAxML (Stamatakis, 2014) to construct a Maximum Likelihood phylogeny from a supermatrix concatenated from 3,141 genes and a total of 3.14 Mbp (Figure 4). This contrasts with earlier phylogenies (Bos et al., 2011, Cui et al., 2013, Morelli et al., 2010, Wagner et al., 2014), which were based on less than 2,300 nucleotides that were ascertained to be variable in *Y. pestis*, likely leading to lower statistical accuracy than with whole-genome analyses. Furthermore, the use of SNPs ascertained to be variable in *Y. pestis* would downwardly bias estimates of branch lengths in *Y. pseudotuberculosis* and lead to underestimates of the *Y. pestis* versus *Y pseudotuberculosis* divergence time, as seen in the branch length of the *Y. pestis* clade to *Y. pseudotuberculosis* (Figure S3). The topology of our whole genome tree shows *Y. pestis* as a monophyletic group within *Y. pseudotuberculosis* with RISE505 and RISE509 (Figure 4A, black arrow, Figure S4) clustered together within the *Y. pestis* clade. The *Y. pestis* sub-tree topology (Figure 4B, Figure S4) is similar to that reported previously (Bos et al., 2011, Cui et al., 2013, Morelli et al., 2010, Wagner et al., 2014), but with the two ancient strains (RISE505 and RISE509) falling basal to all other known strains of *Y. pestis* (100% bootstrap support).

### Determination of *Yersinia pestis* Divergence Dates

**Figure S5:**
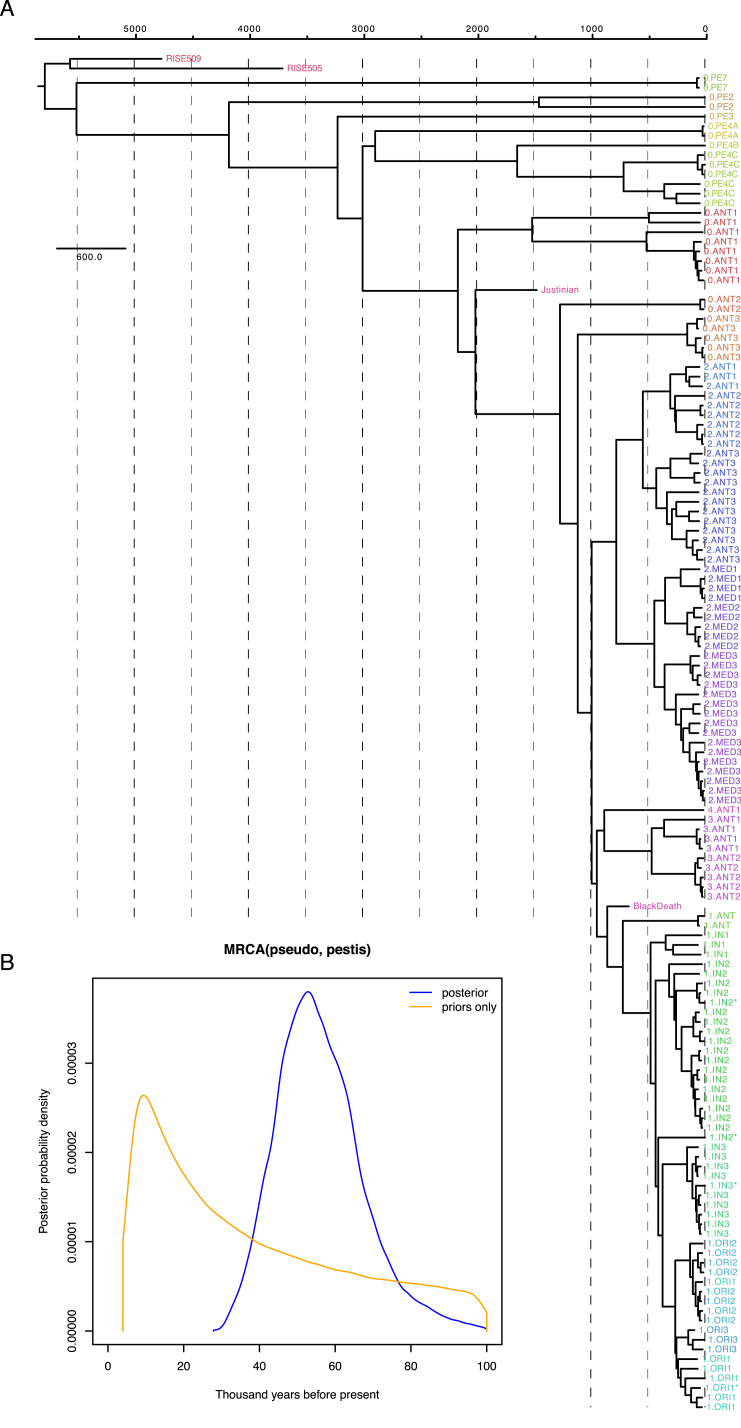
BEAST Divergence Dating, Related to Figures 4 and 6 (A) Maximum clade credibility tree of the *Y. pestis* clade. Strains are annotated based on their population (see Table S2) and colored according to population. Branch lengths are given as years before present. Taxa with asterisks in their name have not previously been assigned a population, but are named according to the clade they are placed in. (B) Posterior probability density distribution for the chain where we sampled from the priors only (orange) and the chains including the alignment data (blue).

To determine the dates for the most recent common ancestor (MRCA) of *Y. pestis* and *Y. pseudotuberculosis,* and for all known *Y. pestis* strains, we used a Bayesian Markov Chain Monte Carlo approach implemented in BEAST2 (Bouckaert et al., 2014) on a subset of the supermatrix. We estimated the MRCA of *Y. pestis* and *Y. pseudotuberculosis* to be 54,735 years ago (95% HPD [highest posterior density] interval: 34,659–78,803 years ago) (Figure 4C, Figure S5, Table S5), which is about twice as old compared to previous estimates of 2,600–28,000 years ago (Achtman et al., 1999, Achtman et al., 2004, Cui et al., 2013, Wagner et al., 2014). Additionally, we estimated the age of the MRCA of all known *Y. pestis* to 5,783 years ago (95% HPD interval: 5,021–7,022 years ago). This is also significantly older and with a much narrower confidence interval than previous findings of 3,337 years ago (1,505–6,409 years ago) (Cui et al., 2013).

### Bronze Age *Yersinia pestis* Strains Lacking Yersinia Murine Toxin

**Figure 5:**
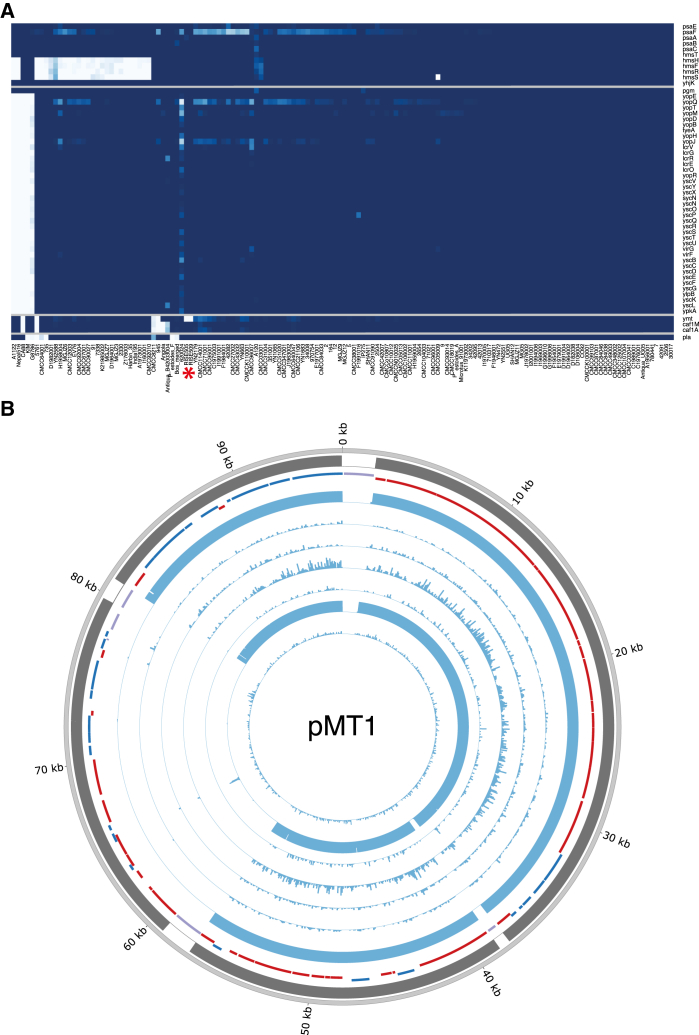
Identification of Virulence Genes (A) Gene coverage heatmap of 55 virulence genes (rows) in 140 *Y. pestis* strains (columns). Sample ordering is based on hierarchical clustering (not shown) of the gene coverage distributions. RISE505 and RISE509 are marked with a red asterisk. Coloring goes from 0% gene coverage (white) to 100% gene coverage (blue). (B) Depth of coverage of high quality reads mapping across pMT1. Outer ring is mappability (gray), genes (RNA: black, transposon: purple, positive strand: blue, negative strand: red) and then the RISE samples ordered after direct AMS dating. Sample ordering are RISE509, RISE511, RISE00, RISE386, RISE139, RISE505 and RISE397. See also Figure S6, Tables S2, S6, and S7. AMS: Accelerator Mass Spectrometry.

For the high-depth ancient *Y. pestis* genomes, we investigated the presence of 55 genes that have been associated with the virulence of *Y. pestis* (Figure 5A, Table S6). We found all virulence genes to be present, except the Yersinia murine toxin (*ymt*) gene that is located at 74.4–76.2 kb on the pMT1 plasmid (Figure 2C, arrow 1). The *ymt* gene encodes a phospholipase D that protects *Y. pestis* inside the flea gut, thus enabling this enteric bacteria to use an arthropod as vector; it further allows for higher titers of *Y. pestis* and higher transmission rates (Hinnebusch, 2005, Hinnebusch et al., 2002). When investigating all seven samples for the presence of *ymt*, we identified a 19 kb region (59–78 kb, Figure 2C arrow 2–3, Figure 5B) to be missing except in the youngest sample (RISE397, 951 cal BC) (Figure 5B, Table S7). We find this region to be present in all other published *Y. pestis* strains (modern and ancient), except three strains (5761, 945, and CA88) that are lacking the pMT1 plasmid completely.

**Figure S6:**
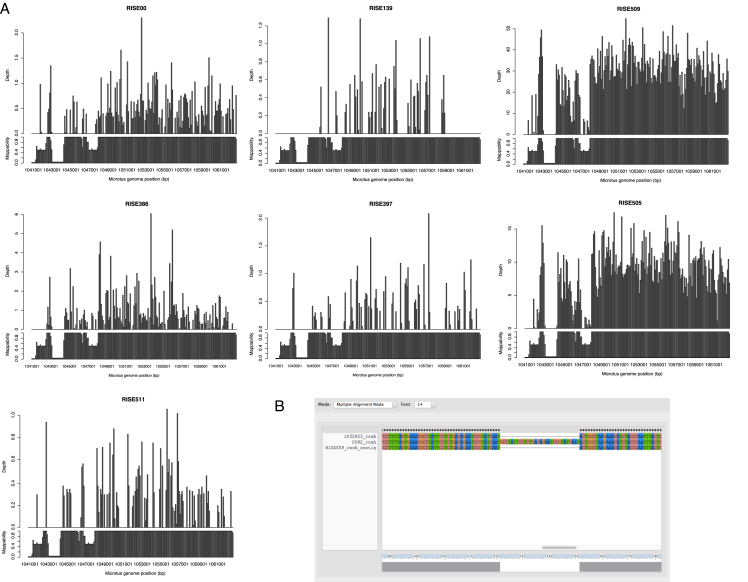
Investigation of Virulence Genes, Related to Figure 5 (A) Depth of coverage for the seven ancient *Y. pestis* samples in 100 bp bins across *Y. pestis Microtus* 91001 genome at 1,041 kb to 1,063 kb. For each sample the upper panel represents the depth of high quality reads in the 100 bp window. The lower panel represent mappability of the particular region calculated using GEM-mappability with a k-mer of 50. (B) Multiple alignment of the *rcsA* gene in *Y. pseudotuberculosis* IP32953, *Y. pestis* CO92 and the contig matching the region from the RISE509 *de novo* assembly. The 30 bp internal duplication in CO92 is absent from the RISE509 sequence that therefore carries the ancestral IP32953 *rcsA* genotype.

Although larger sample sizes are needed for confirmation, our data indicate that the *ymt* gene was not present in *Y. pestis* before 1686 cal BC (n = 6), while after 951 cal BC, it is found in 97.8% of the strains (n = 140), suggesting a late and very rapid spread of *ymt*. This contrasts with previous studies arguing that the *ymt* gene was acquired early in *Y. pestis* evolution due to its importance in its life cycle (Carniel, 2003, Hinnebusch, 2005, Hinnebusch et al., 2002, Sun et al., 2014). Interestingly, we identified two transposase elements flanking the missing 19 kb region, confirming that the *ymt* gene was acquired through horizontal gene transfer, as previously suggested (Lindler et al., 1998). Moreover, it has recently been shown that the transmission of *Y. pestis* by fleas is also dependent on loss of function mutations in the *pde2*, *pde3*, and *rcsA* genes (Sun et al., 2014). The RISE509 sample carries the promoter mutation of *pde3* and the functional *pde2* and *rcsA* alleles (Figure S6). In combination with the absence of *ymt*, these results strongly suggest that the ancestral *Y. pestis* bacteria in these early Bronze Age individuals were not transmitted by fleas.

### Native Plasminogen Activator Gene Present in Bronze Age *Yersinia pestis*

Another hallmark gene of *Y. pestis* pathogenicity is the plasminogen activator gene *pla* (omptin protein family), located on the pPCP1 plasmid (6.6–7.6 kb). The gene facilitates deep tissue invasion and is essential for development of both bubonic and pneumonic plague (Sebbane et al., 2006, Sodeinde et al., 1992, Zimbler et al., 2015). We identify the gene in six of the seven genomes, but not in RISE139, the sample with the lowest overall depth of coverage (0.75X on pPCP1) (Figure 2D, arrow 4, Table S6). Recently, it has been proposed that pPCP1 was acquired after the branching of the 0.PE2 clade (Zimbler et al., 2015); however, we identified pPCP1 in our samples, including in the 0.PE7 clade (strains 620024 and CMCC05009), which diverged prior to the common ancestor of the 0.PE2 lineage (Figure 4B, Figure 5A). This shows that pPCP1 and *pla* likely were present in the most basal *Y. pestis* (RISE509), suggesting that the 0.PE2 strains lost the pPCP1 plasmid. Interestingly, three 2.ANT3 strains (5761, CMCC64001, and 735) are also missing the *pla* gene, indicating that the loss of pPCP1 occurred more than once in the evolutionary history of *Y. pestis*.

Additionally, we investigated whether RISE397, RISE505, and RISE509 had the isoleucine to threonine mutation at amino acid 259 in the Pla protein. This mutation has been shown to be essential for developing bubonic, but not pneumonic, plague (Zimbler et al., 2015). We found that these samples, in agreement with their basal phylogenetic position, carry the ancestral isoleucine residue. However, we also identified a valine to isoleucine mutation at residue 31 for RISE505 (1686 cal BC) and RISE509 (2746 cal BC). This mutation was not found in any of the other 140 *Y. pestis* strains, but was present in other omptin proteins, such as *Escherichia coli* and *Citrobacter koseri*, and very likely represents the ancestral *Y. pestis* state. The youngest of the samples, RISE397 (951 cal BC) carries the derived isoleucine residue, showing that this mutation, similar to the acquisition of *ymt*, was only observed after 1686 cal BC.

An alternative explanation to the acquisition of *ymt* and the *pla* I259T mutation, given the disparate geographical locations of our samples, could be that the Armenian strain (RISE397, 951 cal BC) containing *ymt* and the isoleucine residue in *pla* had a longer history in the Middle East and experienced an expansion during the 1st millennium BC. This would have led to its export to Eurasia and presumably the extinction of the other more ancestral and less virulent *Y. pestis* strains.

### Different Region 4 Present in the Ancestral *Yersinia pestis*

Besides the 55 pathogenicity genes, we also investigated the presence of different region 4 (DFR4) that contains several genes with potential role in *Y. pestis* virulence (Radnedge et al., 2002). This region was reported as present in the Plague of Justinian and Black Death strains, having been lost in the CO92 reference genome (from the Third Pandemic) (Chain et al., 2004, Wagner et al., 2014). Consistent with the ancestral position of our samples, we find evidence that the region is present in all of our seven samples (Figure S6).

### *Yersinia pestis* flagellar Frameshift Mutation Absent in Bronze Age Strains

Another important feature of *Y. pestis* is the ability to evade the mammalian immune system. Flagellin is a potent initiator of the mammalian innate immune system (Hayashi et al., 2001). *Y. pseudotuberculosis* is known to downregulate expression of flagellar systems in a temperature-dependent manner, and none of the known *Y. pestis* strains express flagellin due to a frameshift mutation in the *flhD* regulatory gene (Minnich and Rohde, 2007). However, we do not find this mutation in either RISE505 or RISE509, suggesting that they have fully functional *flhD* genes and that the loss of function occurred after 2746 cal BC. Interestingly, the youngest of these two *Y. pestis* genomes (RISE505, 1686 cal BC) shows partial loss of one of the two flagella systems (758–806 kb), with 39 of 49 genes deleted (Figure 2A, arrow 5, Table S8). This deletion was not found in any of the other *Y. pestis* samples (n = 147). This may point to selective pressure on ancestral *Y. pestis* when emerging as a mammalian pathogen, yielding variably adaptive strains.

## Discussion

**Figure 6:**
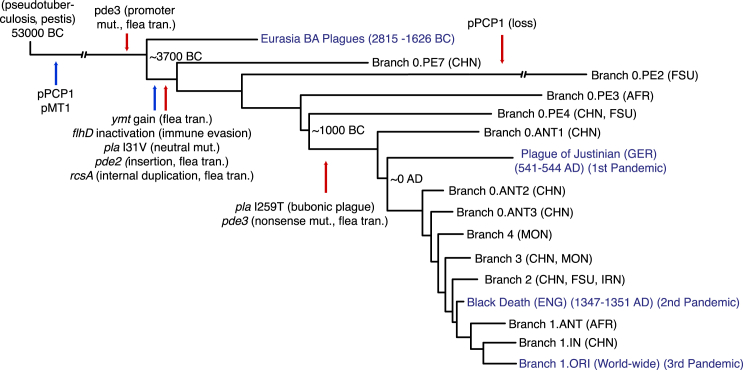
Schematic of *Y. pestis* Evolution Representation of *Y. pestis* phylogeny and important evolutionary events since divergence from *Y. pseudotuberculosis*. Genetic gains (blue) and genetic loss or loss of function mutations (red) are indicated by arrows. Historical recorded pandemics are indicated in blue text. The calendric years indicates the primary outbreak of the Pandemic. Node dates are median divergence times from the BEAST analysis. The events are based on information from this study and Sun et al., 2014. We used the VCFs generated from all *Y. pestis* samples (n = 142) (Table S2) to verify on which branches the genetic events occurred. The figure is based on current knowledge and is subject to change with addition of new samples. See also Figure S5 and Table S5. BA: Bronze Age, CHN: China, FSU: Former Soviet Union, AFR: Africa, GER: Germany, MON: Mongolia, IRN: Iran, ENG: England, flea tran: flea transmission, mut.: mutation.

Our calibrated molecular clock pushes the divergence dates for the early branching of *Y. pestis* back to 5,783 years ago, an additional 2,000 years compared to previous findings (Table S5, Figure S5) (Cui et al., 2013, Morelli et al., 2010). Furthermore, using the temporally stamped ancient DNA data, we are able to derive a time series for the molecular acquisition of the pathogenicity elements and immune avoidance systems that facilitated the evolution from a less virulent bacteria with zoonotic potential, such as *Y. pseudotuberculosis*, to one of the most deadly bacteria ever encountered by humans (Figure 6).

From our findings, we conclude that the ancestor of extant *Y. pestis* strains was present by the end of the 4^th^ millennium BC and was widely spread across Eurasia from at least the early 3^rd^ millennium BC. The occurrence of plague in the Bronze Age Eurasian individuals we sampled (7 of 101) indicates that plague infections were common at least 3,000 years earlier than recorded historically. However, based on the absence of crucial virulence genes, unlike the later *Y. pestis* strains that were responsible for the first to third pandemics, these ancient ancestral *Y. pestis* strains likely did not have the ability to cause bubonic plague, only pneumonic and septicemic plague. These early plagues may have been responsible for the suggested population declines in the late 4^th^ millennium BC and the early 3^rd^ millennium BC (Hinz et al., 2012, Shennan et al., 2013).

It has recently been demonstrated by ancient genomics that the Bronze Age in Europe and Asia was characterized by large-scale population movements, admixture, and replacements (Allentoft et al., 2015, Haak et al., 2015), which accompanied profound and archaeologically well-described social and economic changes (Anthony, 2007, Kristiansen and Larsson, 2005). In light of our findings, it is plausible that plague outbreaks could have facilitated—or have been facilitated by—these highly dynamic demographic events. However, our data suggest that *Y. pestis* did not fully adapt as a flea-borne mammalian pathogen until the beginning of the 1^st^ millennium BC, which precipitated the historically recorded plagues.

## Experimental Procedures

### Samples and Archaeological Sites

We initially re-analyzed the data from Allentoft et al. (Allentoft et al., 2015) and identified *Y. pestis* DNA sequences in 7 of the 101 individuals. Descriptions of the archaeological sites are given in Supplemental Experimental Procedures and Table S1.

### Generation of Additional Sequence Data

In order to increase the depth of coverage on the *Y. pestis* genomes we sequenced more on these seven DNA extracts. Library construction was conducted as in (Allentoft et al., 2015). Briefly, double stranded and blunt-ended DNA libraries were prepared using the NEBNext DNA Sample Prep Master Mix Set 2 (E6070) and Illumina-specific adapters (Meyer and Kircher, 2010). The libraries were “shot-gun” sequenced in two pools on Illumina HiSeq2500 platforms using 100-bp single-read chemistry. We sequenced 32 lanes generating a total of 11.2 billion new DNA sequences for this study. Reads for the seven *Y. pestis* samples are available from ENA: PRJEB10885. Individual sample accessions numbers are available in Table S2.

### Creation of Database for Identification of *Y. pestis* Reads

To identify *Y. pestis* reads in the Bronze Age dataset (Allentoft et al., 2015) we first created a database of all previously sequenced *Y. pestis* strains (n = 140), *Y. pseudotuberculosis* strains (n = 30), *Y. similis* strains (n = 5), and a selection of *Y. enterocolitica* strains (n = 4) (Supplemental Experimental Procedures and Table S2). The genomes were either downloaded from NCBI or downloaded as reads and de novo assembled using SPAdes-3.5.0 (Bankevich et al., 2012) with the–careful and–cov-cutoff auto options.

### Identification and Assembly of *Y. pestis* From Ancient Samples

Raw reads were trimmed for adaptor sequences using AdapterRemoval-1.5.4 (Lindgreen, 2012). Additionally leading and trailing Ns were removed as well as bases with quality 2 or less. Hereafter, the trimmed reads with a length of at least 30 nt were mapped using bwa mem (local alignment) (Li and Durbin, 2009) to the database of *Y. pestis*, *Y. pseudotuberculosis*, *Y. similis*, and *Y. enterocolitica* mentioned above. Reads with a match to any of the sequences in this database were aligned separately to three different reference genomes: *Yersinia pestis* CO92 genome including the associated plasmids pCD1, pMT1, pPCP1 (Parkhill et al., 2001); *Yersinia pseudotuberculosis* IP32953 including the associated plasmids (Chain et al., 2004); *Yersinia pestis biovar Microtus* 91001 and associated plasmids (Zhou et al., 2004). This alignment was performed using bwa aln (Li and Durbin, 2009) with the seed option disabled for better sensitivity for ancient data, enforcing global alignment of the read to the reference genome. Each sequencing run was merged to library level and duplicates removed using Picard-1.124 (http://broadinstitute.github.io/picard/), followed by merging to per sample alignment files. These files were filtered for a mapping quality of 30 to only retain high quality alignments and the base qualities were re-scaled for DNA damage using MapDamage 2.0 (Jónsson et al., 2013). We defined *Y. pestis* as present in a sample if the mapped depth of the CO92 reference sequences were higher or equal to 0.1X and if the reads covered at least 10% of the chromosome and each of the plasmids. The assembly of Justinian, Black Death, and the modern samples were performed similarly and is described in detail in the Supplemental Experimental Procedures.

### Coverage, Depth and Mappability Analyses

We calculated the coverage of the individual sample alignments versus the *Y. pestis* CO92 reference genome using Bedtools (Quinlan and Hall, 2010) and plotted this using Circos (Krzywinski et al., 2009). For the chromosome, the coverage was calculated in 1 kbp windows and for the plasmids in 100 bp windows. Mappability was calculated using GEM-mappability library using a k-mer size of 50, which is similar to the average length of the trimmed and mapped *Y. pestis* reads (average length 43–65 bp). Statistics of the coverage and depth are given in Tables S3 and S4.

### DNA Decay Rates

We investigated the molecular degradation signals obtained from the sequencing data. Based on the negative exponential relationship between frequency and sequence length, we estimated for each sample the DNA damage fraction (λ, per bond), the average fragment length (1/ λ), the DNA decay rate (k, per bond per year), and the molecular half-lives of 100 bp fragments (Allentoft et al., 2012). We compared these DNA decay estimates for *Y. pestis* to the decay of endogenous human DNA from the host individuals. If the plague DNA is authentic and ancient, a correlation is expected between the rate of DNA decay in the human host and in *Y. pestis*, because the DNA has been exposed to similar environmental conditions for the same amount of time. See Supplemental Experimental Procedures for additional information.

### Comparison of Samples to *Y. pestis* and *Y*. *pseudotuberculosis* Reference Genomes

We used the alignments of several sets of reads (*Y. pestis*, *Y. pseudotuberculosis*, and *Y. similis*) to *Y. pestis* CO92 and the *Y. pseudotuberculosis* IP32953 genomes. Per sample we determined the distribution of edit-distances (mismatches) of the reads versus the particular reference genome. We used these distributions to build a Naive Bayesian classifier to classify whether reads were originating from *Y. pestis*, *Y. pseudotuberculosis*, or *Y. similis*. See Supplemental Experimental Procedures and Table S3.

### Expected versus Actual Coverage

We estimated the expected coverage of *Y. pestis* given a specific sequencing depth and correlated that with the actual coverage of a genome per sample. Expected coverage was calculated as

$$c=1-\prod_{i=1}^{N}{\left(1-\frac{l_{i}}{g}\right)}^{r_{i}}$$

where the reads have N different lengths, l_1_ to l_N_ with counts r_1_ to r_N_. To account for mappability we determined the mappable fraction for each reference sequence using kmers of length 40, 50, and 60, and then used the mappability value with the k-mer length closest to the actual average read length for each sample/reference combination. For more information see Supplemental Experimental Procedures.

### Genotyping For Phylogenetic Analyses

Alignments of all strains versus *Y. pseudotuberculosis* IP32953 was used as reference for genotyping the consensus sequences for all samples used in the phylogeny. The samples were genotyped individually using samtools-0.1.18 and bcftools-0.1.17 (Li et al., 2009) and hereafter filtered (Supplemental Experimental Procedures). Based on *Y. pseudotuberculosis* IP32953 gene annotations, the consensus sequences for each gene and sample were extracted. Because of the divergence between *Y. pestis* and *Y. pseudotuberculosis*, a number of gene sequences displayed high rates of missing bases and we removed genes where 20 or more modern *Y. pestis* samples had >10% missingness. This corresponded to a total of 985 genes, leaving data from 3,141 genes that were merged into a supermatrix. We created two different supermatrices, one with *Y. similis*, *Y. pseudotuberculosis*, and *Y. pestis* containing 173 taxa × 3,141 genes that was used for the initial phylogeny (Figure 4A). The second supermatrix consisted of all *Y. pestis* strains and the genomes from the two closest *Y. pseudotuberculosis* clades, which was used for the divergence time estimations.

### Phylogenetics

The alignments were partitioned by codon position and analyzed with jmodeltest-2.1.7 (Darriba et al., 2012) to test for the best fitting substitution model. All decision criteria (Akaike, Bayesian, and Decision theory) found the Generalized Time Reversible substitution model with gamma distributed rates, using four rate categories, and a proportion of invariable sites (GTR+G+I) to be the best fit for each of the three codon partitions. To test for recombination across the chromosome we estimated linkage disequilibrium (LD) using 141 *Y. pestis* strains. A total of 482 bi-allelic single nucleotide variations (SNVs), with a minor allele frequency of 5% or higher were extracted. For all pairs of the extracted SNVs, the LD *r*^2^ was calculated using PLINK 1.9 (Chang et al., 2015) and plotted against the physical distance between the pairs. We reconstructed the phylogeny from the codon-partitioned supermatrix using RAxML-8.1.15 (Stamatakis, 2014) with the GTR+G+I substitution model. Bootstraps were performed by generating 100 bootstrap replicates and their corresponding parsimony starting trees using RAxML. Hereafter, a standard Maximum Likelihood inference was run on each bootstrap replicate, and the resulting best trees were merged and drawn on the best ML tree. Initial phylogenies placed the *Y. pestis* Harbin strain with an unusual long branch inside the 1.ORI clade and it was excluded from further analysis. Additionally *Y. pseudotuberculosis* SP93422 (serotype O:15), *Y. pseudotuberculosis* WP-931201 (serotype O:15) and *Y. pseudotuberculosis* Y248 (serotype unknown) was in a clade with long branch lengths and were therefore also omitted (see Figure S4).

### Heterozygosity Estimates

We determined heterozygosity by down-sampling the *Y. pestis* bam-files to the same average depth as the corresponding RISE samples, genotyped each of the samples and extracted heterozygote calls with a depth equal to or higher than 10. All transitions were excluded. See Supplemental Experimental Procedures for detailed information.

### Divergence Estimations

To date the divergence time for *Y. pestis* and nodes within the *Y. pestis* clade we performed Bayesian Markov Chain Monte Carlo simulations using BEAST-2.3.0 (Bouckaert et al., 2014) and the BEAGLE library v2.1.2 (Ayres et al., 2012). We used the codon-partitioned supermatrix that included the two closest *Y. pseudotuberculosis* clades, with unlinked substitution models, GTR+G+I with eight gamma rate categories and unlinked clock models. Dates were set as years ago with the RISE509, RISE505, Justinian and Black Death samples set to 4,761, 3,701, 1,474, and 667 years ago, respectively. All unknown dates were set to 0 years ago. We followed previous work (Cui et al., 2013, Wagner et al., 2014) and applied a lognormal relaxed clock, assuming a constant population size. We re-rooted the ML tree from RAxML so that the root was placed between the two *Y. pseudotuberculosis* clades (IP32953, 260, IH111554) and (IP32921, IP32881, IP32463) and used this as the starting tree. Based on the ML tree we defined the closets *Y. pseudotuberculosis* clade (IP32921, IP32881, IP32463) and the *Y. pestis* clade as a monophyletic group and defined a uniform prior with 1,000 and 100,000 years as minimum and maximum bounds. We ran 20 independent parallel BEAST chains sampling every 2,000 states for between 52 and 64 million states using a total of 240,000 core hours. The chains were combined using LogCombiner discarding the initial 10 million states as burn-in. The combined post burn-in data represented 961 million states and the effective sample sizes (ESS) for the posterior was 398, for the TreeHeight 238 and for the MRCA for *Y. pseudotuberculosis* and *Y. pestis* 216. All other parameters had ESS > 125. We then sampled 1/5 of the trees from each chain and combined them for a total of 192,406 trees that were summarized using TreeAnnotator producing a maximum clade credibility tree of median heights. We additionally ran BEAST2 sampling the priors only (and disregarding sequence information) and found the posterior distribution no different than the priors used. It suggests that the posterior distributions recovered when considering full sequence alignments are driven by the sequence information and are not mere by-products of the sampling structure in our dataset (Figure S5).

### Analysis of Virulence Associated Genes

To assess the potential virulence of the ancient *Y. pestis* strains, we identified 55 genes previously reported to be associated with virulence of *Y. pestis* (Supplemental Experimental Procedures and Table S6 for details). Based on the alignments to *Y. pestis* CO92 reference genome we determined the fraction of the each gene sequence that was covered by at least one read for each *Y. pestis* sample. Additionally, because the different region 4 (DFR4) (Radnedge et al., 2002) has been associated with virulence, but is not present in the CO92 genome, we used the alignments to *Y. pestis microtus* 91001 to determine the presence of this region (Supplemental Experimental Procedures). We note that the absence of KIM pPCP1 is due to it being missing from the reference genome, but that it has been reported to be present in KIM strains (Hu et al., 1998). The genotypes were generated as described above and the variant call format (VCF) files from these analyses are available at http://www.cbs.dtu.dk/suppl/plague/. For detailed information on genotyping of *pde2*, *pde3*, *rscA*, *pla*, and *flhD* see Supplemental Experimental Procedures.

### Identification of the Missing *ymt* Region on pMT1

Most of the regions that were unmapped could be associated with low mappability. However, we identified a region from 59–78 kb on pMT1 that could not be explained by low mappability. From the depth of coverage this region was absent in all of our ancient plague genomes, except for RISE397 (Figure 5). We tested for the significance of this by comparing the distribution of gene depths within and outside of the missing region using the Wilcoxon rank-sum test (Table S7). For all samples except RISE397 the region had a median depth of 0X and the gene depth distributions were significantly different compared to the remaining pMT1 plasmid genes (p values < 1E-9). For the RISE397 sample, the regions had 0.43X and 0.42X median depths and there was no significant difference in the depth of the genes in the two regions (p value 0.77).

## Author Contributions

Conceptualization, K-G.S., R.N., K.K. and E.W.; methodology, S.R., M.E.A., A.G.P. and H.B.N.; software, S.R., K.N., M. Sikora, M. Schubert, and A.V.D.; Formal Analysis, S.R., M.E.A., K.N., M. Sikora, A.G.P., A.V.D. and M. Schubert.; Investigation, M.E.A. and K-G.S.; Resources, S.B., P.A., M.V.K., A.E., A. Gnuni, A.K., I.L., M.M., V.M., A. Gromov, D.P., L.S., L.V., L.Y. and T.S-P.; Writing – Original Draft, S.R., M.E.A., K.N., L.O., K-G.S., A.G.P., R.A.F., M.M.L., R.N., K.K. and E.W.; Writing Review & Editing, S.R., M.E.A., K.N., L.O., M. Sikora, K-G.S., A.G.P., A.V.D., C.M.O., R.A.F., M.M.L., R.N., K.K. and E.W.; Visualization, S.R. M.E.A., K-G.S. and A.G.P.; Supervision, L.O., T.S-P., R.N., K.K. and E.W.; Funding Acquisition, K.K. and E.W.

## Figures and Tables

**Table 1:** Overview of the *Y. pestis* Containing Samples

Sample	Country	Site	Culture	Date (cal BC)	CO92	pMT1	pPCP1	pCD1
RISE00	Estonia	Sope	Corded Ware	2575–2349	0.39	0.36	1.40	0.66
RISE139	Poland	Chociwel	Unetice	2135–1923	0.14	0.24	0.76	0.28
RISE386	Russia	Bulanovo	Sintashta	2280–2047	0.82	0.96	1.12	1.60
RISE397	Armenia	Kapan	EIA	1048–885	0.25	0.40	6.88	0.50
RISE505	Russia	Kytmanovo	Andronovo	1746–1626	8.73	9.15	34.09	17.46
RISE509	Russia	Afanasievo Gora	Afanasievo	2887–2677	29.45	16.96	31.22	50.32
RISE511	Russia	Afanasievo Gora	Afanasievo	2909–2679	0.20	0.24	1.19	0.60

The dating is direct AMS dating of bones and teeth and is given as 95% confidence interval calendar BC years (details are given in Table S1). The columns CO92, pMT1, pPCP1 and pCD1 correspond to sequencing depth. Additional information on the archaeological sites and mapping statistics can be found in the Supplemental Experimental Procedures and Table S1, S2, and S3. EIA: Early Iron Age, AMS: Accelerator Mass Spectrometry.

## Accession Numbers

The accession number for the reads for the seven *Y. pestis* samples reported in this paper is ENA: PRJEB10885.

## Supplemental Information

Document S1. Supplemental Experimental Procedures

Document S2. Tables S1–S8

Document S3. Article plus Supplemental Information
