# Supplementary material for: Early Divergent Strains of Yersinia pestis in Eurasia 5,000 Years Ago
Source: Cell. 2015 Oct 22;163(3):571–82. doi: 10.1016/j.cell.2015.10.009 (PMC4644222; doi:10.1016/j.cell.2015.10.009)
Supplement: Document S1. Supplemental Experimental Procedures [file mmc1.pdf]

Cell
Supplemental Information

Early Divergent Strains of *Yersinia pestis* in Eurasia 5,000 Years Ago

Simon Rasmussen, Morten Erik Allentoft, Kasper Nielsen, Ludovic Orlando, Martin Sikora, Karl-Göran Sjögren, Anders Gorm Pedersen, Mikkel Schubert, Alex Van Dam, Christian Moliin Outzen Kapel, Henrik Bjørn Nielsen, Søren Brunak, Pavel Avetisyan, Andrey Epimakhov, Mikhail Viktorovich Khalyapin, Artak Gnuni, Aivar Kriiska, Irena Lasak, Mait Metspalu, Vyacheslav Moiseyev, Andrei Gromov, Dalia Pokutta, Lehti Saag, Liivi Varul, Levon Yepiskoposyan, Thomas Sicheritz-Pontén, Robert Foley, Marta Mirazón Lahr, Rasmus Nielsen, Kristian Kristiansen, and Eske Willerslev

Supplemental Experimental Procedures

Samples and archeological sites

Short summaries of archaeological context and previous analyses performed on the sites where *Y. pesti*s was detected are provided here. Dating and stable isotope analyses on collagen are summarized in Table S1.

Sope, Estonia, Corded Ware Culture

The cemetery at Sope is situated in a coastal area in the north-eastern part of Estonia, about 1.8 km inland from the present Baltic Sea shoreline. Altogether around 10 individuals have been found. Seven individuals were unearthed during farming at the beginning of the 20th century and were later reburied (Lõugas et al., 2007). Archaeological excavations carried out in 1926 (Moora, 1932) and in 1933 (Indreko, 1935) each recovered one female skeleton – Sope I and Sope II respectively (Aul, 1935).

The deceased in Sope had been inhumed in a crouched position, which is characteristic for the Corded Ware Culture throughout the Eastern Baltic. According to osteological analysis the stature of Sope I had been 155.12 ± 3.72 cm (maximum length of the left femur 40.9 cm) and she died at the age of 25–35. The height of the second female (Sope II) was 160.56 ± 3.72 cm (maximum length of the left femur 43.1 cm) and her age at death was 22–24 years. An additional right femur was found co-mingled with Sope I belonging to an adult male whose height had been 167.72 ± 3.72 cm (maximum length of the bone 46 cm).

Only one individual was sampled for DNA from this site. The sample, RISE00, was an upper left M2, taken from the Sope I female. The female sex was confirmed through DNA sexing (Allentoft et al., 2015). She appeared to be buried in a crouched position while lying on her back with her skull towards south-east. The length of her remains was about a meter. However the skeleton was not articulated. For example the left femur was on the right side of the "body" and vice-versa, the proximal end of the left tibia was facing away from the skull and the right tibia was next to the left femur being almost parallel to it. As the skeleton was disarticulated and no metacarpals or metatarsals were found (Aul, 1935) it has been suggested that the initial decomposition of the body happened elsewhere and the skeletonized remains were gathered and wrapped inside something and then buried in the cemetery at Sope (Jonuks, 2009).

The individual was recovered with few items: an awl and a worked bone made from sheep/goat were next to the remains, and a fragment of an unidentified artifact from cattle was near the mandible, underneath which was a pig tooth (Kriiska et al., 2007).

Both females have been AMS (Accelerator Mass Spectrometry) dated (Lasak, 1996) (Table S1). δ13C on Sope I indicates protein mainly from terrestrial sources, despite the location close to the coast. δ15N is quite low, suggesting a substantial input of protein from vegetable sources.

Chociwel, Poland, Unetice culture

Chociwel is situated just a few kilometers north of Strzelin, at the foreground of the Sudetes Mountains. The site was discovered in 1993. In 1995, during construction works, part of the Únětice necropolis was excavated. In 2010, a new group of burials including three females, a young male and a child were excavated (Pokutta, 2013).

Chociwel is a multi-period site with Funnel Beaker and Globular Amphorae settlements (Cholewa, 1998), along with an Early Bronze Age (EBA) cemetery and features dated to the Migration Period and later medieval times. The EBA graves were arranged in north-south rows. The deceased were oriented E-W and only one burial resembles the north-south body orientation usual for Unetice burials.

The burials in Chociwel display a moderate number of artifacts, primarily consisting of between 2 and 5 ceramic vessels.

Four individuals were sampled for DNA from this site. The sample in which plague was detected was RISE139, from grave 20. This grave contained a skeleton osteologically determined as a female of mature age, but DNA analysis showed it to be a male (Allentoft et al., 2015).

Nine individuals have been dated (Pokutta, 2013) (Table S1). The chronometric dating is consistent with the archaeological assignment to the Unetice period, and the individual from which RISE139 was taken seems to be one of the first buried at the site. Stable isotopes have also been measured, but it is difficult to link these with the individuals sampled for DNA (Pokutta, 2013). C/N was not measured at the Uppsala lab, but in Stockholm. A high C/N value from RISE139 indicates measurement from this individual should not be trusted. The high δ15N value from grave 21/2011 is from a milk tooth and probably due to lactation

effects. Other stable isotope values from the site are within the usual range for European Neolithic/EBA populations.

Bulanovo, Russia, Sintashta culture

Cemetery, excavated by Khalyapin 2001-2002 (Khalyapin, 2001).

The dead were buried in shallow pits without barrows (Figure 1). The skeletons were laid in elongated position on their back. The inventory was not numerous (triangular stone arrowheads and bronze shape with bone-handled saws). Burial features (no mound, shallow graves, position of the dead, and lack of pottery and animal sacrifices) and the appearance of grave goods have analogies among Seima-Turbino sites. The Bulanovo cemetery can be seen as the result of interaction between the Sintashta population and the bearers of Seima-Turbino traditions.

The sample RISE386 in which plague was detected is from burial 6, individual 1. This contained bones from an adult male, 30-40 years old. DNA confirms male sex (Allentoft et al., 2015).

Three individuals were sampled from this site, two of which had good DNA preservation (Allentoft et al., 2015). All three were dated, see Table S1. The dates are in relatively good agreement, but it should be noted that RISE386 and 387 have elevated δ15N values, suggesting an intake of protein from freshwater fish, and a freshwater reservoir effect on the dates. These two dates should therefore be reduced somewhat. This is less pronounced in the third individual.

Kapan-Shahumyan, Armenia, Early Iron Age

The excavation was conducted in 2012 by Dr. Artak Gnuni near the village Shahumyan (Syunik region of Armenia). The site is located in the hills adjacent to the left bank of the river Voghji, 5 km north-east of the city of Kapan. The survey revealed the presence of a multilayer settlement and a burial ground dated to Early Iron Age. In total eleven complete burials were excavated.

Four individuals were sampled, two of which had good DNA preservation, both from burial No. 6. The tomb was a small stone chamber built of medium size stones, oriented from north to south. The upper horizon of the burial was disturbed and the stones of the ceiling were absent. The degree of the walls sloping inward implied the presence of a false vault.

Stones and clay of brown color filled the grave, with a small impurity of sand. The filling contained small fragments of ceramics, including fire exposed items and drop-shaped beads. From the north-eastern part of the burial, a fragment of a phalange was discovered. In the central part of the southern wall a carnelian bead was found. A badly burned bone fragment was also found in the central part of the tomb. The filling contained distinct patches of ash.

The burial had two skeletons. The first one (sample RISE396a) was in a crouched position on the right side and oriented from the north-east to south-west, head turned to the north. It was located on the western part of the tomb under the south wall. This individual had a massive bracelet on one hand, two more on the other, as well as a ring on the skull. There were also several ceramic items under the skeleton, two pitchers (under the skull and the pelvis), and a bowl (under the shoulder-blade). This individual had been determined as an adult female, 20-25 years old. This was confirmed by DNA sexing (Allentoft et al., 2015).

To the east of the skeleton, near the southern wall, was a stone that separated the two skeletons.

The skull of the second individual (sample RISE397a) was found in the northeast corner with a ring on the skull, similar to the first individual. The bones were in a very decayed condition, with only those of the extremities well preserved. The bones were osteologically determined as a juvenile female, 15-18 years old, but DNA analysis indicated that they actually are from a male (Allentoft et al., 2015).

A specific feature of the burials is the presence of tiny pitchers. These vessels, evidently the objects of worship, are broadly encountered artifacts in the early Iron Age monuments in Armenia. Analysis of the inventory allows the dating of the burial to the X-VIII cent. BC.

The dating of the two individuals supports this chronology (Table S1). Skeleton 1 may be slightly older, although contemporaneity is not excluded. Low $\delta 15N$ values suggest an unusually high proportion of vegetable protein, possibly including C4 plants since $\delta 13C$ is also somewhat elevated.

Kytmanovo, Russia, Andronovo culture

The Kytmanovo burial ground was excavated by A. P. Umanski in 1961-1963. Most burials were attributed to the Andronovo culture (Umanski et al., 2007). Altogether 37 graves were excavated.

The individual with plague is sample RISE505, collection number 6652-42, burial 20. It is located in the center of the Kytmanovo burial ground.

According to description and visual data there were three individuals in the grave – one adult and two children. One of the children is an infant, less than one year old. The baby was laid in front of the adult. Although the majority of the Andronovo people were buried on their left side in crouched position some small infants were buried on their right side as if they are looking at adults. This is exactly what we see in this case. The adult is probably female, 30-35 years old. Female sex was confirmed by DNA sexing (Allentoft et al., 2015). The second child according to Umanski et al., (2007) is about 4-6 years old. Regrettably we cannot check this proposition because no child bones survived.

Archeological data suggest all burials in this grave were simultaneous. This indirectly supports plague as a cause of death of these people since this is the only case in the Kytmanovo (all other burials are single or double).

The objects found in the grave are usual for Andronovo people. In this case these are three pots, one for each individual (note that the size of pots corresponds with the age of the buried). Several bronze plaques were associated with the adult. Two of them were located in the os temporalis area and one on the right shoulder. Notably, no gold artefacts were found in the grave. Taking in account the rather poor bronze kit found in the grave we can suggest that individuals from the grave did not belong to the high strata of Andronovo society. Altogether 7 burials from the 37 graves have golden objects.

Altogether five individuals from the site were sampled, all of which had good DNA preservation (Allentoft et al., 2015). All five were dated, but one person turned out to be medieval, while another dating failed, see Table S1. The samples have rather high $\delta 13C$ and also somewhat elevated $\delta 15N$ values which suggests protein input from C4 plants, possibly also from freshwater fish.

Afanasieva Gora (Bateni), Russia, Afanasievo culture

The Afanasieva Gora site is sometimes also called Bateni. It was excavated during the turbulent period of Russia directly after the Revolution and Civil War in 1923 by SA Tephloukhov. Although this is really a focal site on which the Afanasievo culture was recognized, no photos or regular drawings were made during excavation. A short description of the graves is given in Vadetskaya et al. 2014 (pages 124-125 and 301) (Vadetskaya et al.,

2014), based on the diary of Teploukhov. Later, graves were excavated in the 1960s by Gryaznov and these are well documented (Vadetskaya et al., 2014).

The samples RISE509 and RISE511 are both from grave 15. This is a mass grave where bones of 7 individuals were found. The skeletons were from a male (20-35), three females (two of them are 25-30 years old, one possibly older than 40) and three children (one 10-12 years, another 5-8 years old. Bones of the third were lost and no information about age exists). Osteological sexing has been confirmed by DNA (Allentoft et al., 2015).

Because single bones of roe deer, fox and chipmunk were found in the grave, Teploukhov suggested that these bones were mixed occasionally with children bones when they were reburied in the grave from some other place. Also there is one strange observation; Teploukhov remarked that incisors in the male mandible were replaced with premolars tightly placed into the alveolus. At present time most teeth have been lost.

As to artifacts the only ones were fragments of typical Afanasievo egg-shaped pots.

Collective burials are quite unusual for Afanasievo people. Most Afanasievo burials are single ones. Double burials with two adults are just 1-5% of all burials; graves with more individuals are very rare. However, in Afanasieva Gora there is one case with 4 individuals in one grave (grave 24) and and one with 7 individuals (grave 41). While this is a collective burial, in this case the archeologists believe that the burials were made successively.

Three individuals were sampled from this site (RISE509-511), two of which were from burial 15. All three are adult females, one of which is aged 20-25 (RISE509) and two 25-30 years old (RISE510 and RISE511). All three were dated, giving consistent dates, see Table S1. The interpretation of grave 15 as a mass grave is supported by the dates. Δ13C values are relatively high, which could indicate protein sources including C4 plants and/or freshwater fish.

Creation of a database for identification of *Y. pestis* reads

The database for identification of *Y. pestis* reads contained all previously sequenced *Y. pestis* strains (n=140), *Y. pseudotuberculosis* strains (n=30), *Y. similis* strains (n=5) and a selection of *Y. enterocolitica* strains (n=4) (Batzilla et al., 2011; Bos et al., 2011; Chain et al., 2006; Cui et al., 2013; Deng et al., 2002; Eppinger et al., 2007, 2009, 2010; Parkhill et al., 2001; Reuter et al., 2014; Shen et al., 2010; Song et al., 2004; Thomson et al., 2006; Wagner et al., 2014; Wang et al., 2011; Zhang et al., 2009). See Table S2 for details.

Assembly of *Y. pestis* from Justinian and Black Death samples

The Black Death plague data from samples Bos8124, Bos11972 and Bos8291 were downloaded from SRP008060 split into pairs and processed similarly to our ancient samples except that only merged sequences were used (Bos et al., 2011). Finally, the three samples were merged to one representative sample. Data from the Justinian Plague sample A120 was downloaded from SRP033879 and processed similarly to our ancient samples except that only merged and unmerged pair1 reads were used for the downstream analyses (Wagner et al., 2014).

Assembly of *Y. pestis* from modern samples

Data from modern *Y. pestis* samples were obtained by downloading reads from SRA010790 (Cui et al., 2013) and the complete genomes available at NCBI (Table S2). *Y. pseudotuberculosis* data were downloaded as reads from ERP000171 (Reuter et al., 2014). To achieve maximum comparability of data between the samples, we simulated reads from the complete genomes that were downloaded from NCBI. Using ART (Huang et al., 2012) 100 nt paired end error-free reads with an average insert size of 300 nt and depth of 50X were generated. The modern genomes were processed as the ancient samples except that they were not re-scaled for DNA-damage.

Molecular degradation patterns in *Y. pestis* and the human host

The DNA sequence length distribution obtained from shotgun sequencing data carries detailed information about the state of molecular preservation in an ancient sample (Allentoft et al., 2012). In an ancient DNA extract there should be a negative exponential correlation between the number of DNA molecules and their length. This is an effect of random fragmentation of the DNA strands, leaving few long DNA fragments and many short ones (Allentoft et al., 2012; Deagle et al., 2006). In order to validate the authenticity of the sequenced *Y. pestis* DNA we therefore examined the length distribution for all eight samples. Following previous studies (Allentoft et al., 2012; Olalde et al., 2014; Orlando et al., 2013), we investigated only the declining part of the distributions, thereby excluding biases caused by poor recovery of short DNA fragments and a fixed maximum sequencing length. The fragment length distributions for all seven *Y. pestis* datasets conformed well to an exponential decay model (R^2 = 0.94-0.99) (Figure 3 and Figure S1) as expected for ancient DNA.

Deagle et al. (Deagle et al., 2006) showed that the decay constant (λ) in the exponential relationship represents the DNA damage fraction. We estimated λ in the seven *Y. pestis*

datasets to between 0.044 and 0.139 (Figure 3, Table S3 and Figure S1), implying that only 4.4% of the phosphodiester bonds in the DNA backbone are broken in RISE392, whereas 13.9% are broken in RISE509 - the most degraded sample. Moreover, $1/\lambda$ is equivalent to the expected average DNA fragment length (Deagle et al., 2006) and this ranged from 6.6 bp to 22.7 bp in the seven samples (Table S3). These numbers show that the *Y. pestis* DNA is highly degraded as would be expected given the age of the skeletons. We note that the average expected fragment length ($1/\lambda$) is not equivalent to the average sequence length, which is biased both experimentally and bioinformatically.

It has been shown that long-term post mortem DNA fragmentation can be described as a rate process, and that the damage fraction (λ, per bond) can be converted to a decay rate (k, per bond per year), when the age of the sample is known (Allentoft et al., 2012). Using median calibrated radiocarbon ages (Table S3) we get rates of decay from 1.41E-5 to 3.17E-5 strand breaks per site per year, corresponding to molecular half-lives (for 100 bp fragments) of 492 years and 219 years respectively. After this period of time, 50% of all 100 bp stretches in the genome will be lost due to one or more strand breaks (Allentoft et al., 2012).

We also investigated the data for a correlation between DNA degradation patterns in the *Y. pestis* and that of the human host individual. In general the DNA decay proved slower for ancient human DNA than for *Y. pestis* - on average 1.6 times slower (Table S3). This is perhaps not unexpected given that *post mortem* DNA preservation conditions is likely more favorable inside human cells embedded in solid tooth cementum or dentine than they are in bacteria. Importantly, however, there was a correlation between the estimated decay rate of the human host DNA and the *Y. pestis* DNA that was co-extracted from the same individual ($R^2 = 0.55$, $P = 0.055$) (Figure 3). A fast decay rate in the human DNA is accompanied by a fast decay rate in the *Y. pestis* DNA. This apparent link constitutes another argument that the *Y. pestis* is indeed associated with the human remains rather than representing some secondary microbial invasion.

In summary, the fragmentation patterns of the DNA we have identified as *Y. pestis* carry strong signatures of authentic and highly degraded ancient DNA, which would not be expected if the DNA was derived from, for example, modern soil bacteria. Finally, it is worth noting that some of the human DNA sequence distributions display a 10 bp periodicity (Figure 3 and Figure S1). This phenomenon has been described previously in genomic data and is likely reflecting the 10 bp turn of the DNA helix combined with preferential strand cleavage of the DNA backbone facing away from nucleosome protection (Pedersen et al., 2014).

Comparison of samples to *Y. pestis* and *Y. pseudotuberculosis* reference genomes

The sequence of *Y. pestis* is very similar to that of its ancestor, *Y. pseudotuberculosis.* It was therefore important to investigate which of these species our unknown samples more closely resembled. We did this by mapping reads from the eight potential *Y. pestis* samples against both reference genomes (*Y. pestis* CO92 and *Y. pseudotuberculosis* IP32953). For each set of reads we then compared the number of reads mapping with different number of mismatches (different "edit distances") to these two references.

We first mapped several sets of reads from known *Y. pestis* and *Y. pseudotuberculosis* genomes against the two references. For comparison we also included sequences from *Y. similis,* which is an outgroup to both *Y. pestis* and *Y. pseudotuberculosis*. Typical examples of the results of mapping known sequences to the two reference genomes are shown in Figure S2. It is clearly seen that *Y. pestis* samples are slightly closer to the *Y. pestis* genome than to the Y. *pseudotuberculosis* genome: *Y. pestis* samples have more reads matching perfectly to *Y. pestis* than to *Y. pseudotuberculosis* (i.e., more reads mapping with edit distance=0; ratio > 1). The inverse is the case for *Y. pseudotuberculosis* samples, which have fewer perfect matches to *Y. pestis* than to *Y. pseudotuberculosis* (ratio < 1). Samples from *Y. similis* map about equally well to both reference genomes (ratio ~ 1), and have far fewer perfectly matching reads than the other two species (Figure S2).

Figure S2 summarizes the results of mapping several sets of reads from known species to the two reference genomes. For each edit distance, and each of the three investigated species, the distribution of frequencies obtained when mapping to the two references is shown in the form of a boxplot. The phenomena described above can bee seen to hold across many different samples, but with some spread in the actual values. Another way of investigating the closeness of sample reads to the two references, is by computing the ratio of reads mapping to *Y. pestis* vs reads mapping to *Y. pseudotuberculosis.* This is shown in Figure S2, note that the ratio is larger than 1 for perfect matches when a *Y. pestis* sample is used, and less than 1 for the other species.

Figure 3 and Figure S2 show the results of mapping the eight selected RISE samples of unknown origin against the two reference genomes. All samples, except RISE392, were found to be more similar to *Y. pestis* than to *Y. pseudotuberculosis*, and to have the majority of their reads mapping perfectly to *Y. pestis* (edit distance=0). For RISE392 reads mapped about equally well to both *Y. pestis* and *Y. pseudotuberculosis* reference genomes, and there

were fewer reads mapping perfectly (edit distance > 0) than imperfectly, indicating that RISE392 is neither *Y. pestis* nor *Y. pseudotuberculosis*, but a more distantly related species.

Bayesian classification of species assignment for unknown samples

To further quantify the qualitative assessment of read similarities described above, we constructed a naïve Bayesian classifier capable of predicting the species of an unknown sample based on the distribution of read counts mapping at different edit distances to the *Y. pestis* and *Y. pseudotuberculosis* reference genomes. Specifically, our method uses the following 10 values as input ("feature vector"): the *ratio* between reads mapped to *Y. pestis* and reads mapped to *Y. pseudotuberculosis* for edit distance 0 to 4 (these are the first 5 features), and the *frequency* of reads mapping to *Y. pestis* at edit distance 0 to 4 (the last 5 features). The output is the posterior probabilities that the sample is from *Y. pestis*, *Y. pseudotuberculosis*, or *Y. similis*. The method was trained on the data obtained from mapping reads of known origin to the two reference genomes. Details about the classifier are given below.

When the classifier was used to assess the eight unknown RISE samples, it very clearly classified all samples, except RISE392, as *Y. pestis*, with posterior probabilities of 100% (Table S3). RISE392 was found to have 0% posterior probability of being *Y. pestis*, and was instead classified as *Y. similis* (posterior probability = 100%). It should be noted that our method is only capable of classifying unknown samples as one of the three species mentioned above, and that especially samples classified as *Y. similis*, may generally correspond to any non-pestis, non-pseudotuberculosis, more distantly related species.

We also used the method to classify the remaining unknown RISE samples. The majority of these were classified as *Y. similis* (88 of 102 samples), while 13 (including the 7 investigated above) were classified as *Y. pestis* (data not shown). However, most of these samples have very few reads mapping to our *Yersinia* reference genomes, and classifications are therefore very uncertain. Among samples with more than 500 reads mapping to the reference genome, there were 20 classified as *Y. similis*, and 9 classified as *Y. pestis* (again including the 7 samples mentioned above). Table S3 shows the results also for the additional two putative *Y. pestis* samples. Among these, RISE510 was found in the same mass grave as RISE509 and RISE511 (which we are very certain are *Y. pestis*), but due to low number of reads has relatively low posterior probability of being *Y. pestis* (P = 52%).

Naïve Bayesian classifier: technical details

Naïve Bayesian classifiers use a set of input values (the feature vector) as the basis for computing the probability that an unknown data point belongs to one of a number of classes. In the present case the possible classes were the three species *Y. pestis*, *Y. pseudotuberculosis*, and *Y. similis*, and the feature vector consisted of 10 values: 5 ratios (the number of reads mapping to *Y. pestis* vs the number of reads mapping to *Y. pseudotuberculosis*, for edit distance 0 to 4), and 5 frequencies (the fraction of reads mapping to pestis at edit distance 0 to 4).

Naïve Bayesian classification is based on two main ideas: First, it is assumed that the individual features are independent, conditional on the class, even though this is often incorrect (hence "naïve"). It has been shown that despite this overly simplified assumption, naïve Bayesian classification often has very good performance in classification (Hand and Yu, 2001; Zhang, 2004). The assumption of independence means that it is possible to compute the joint probability of observing any set of feature values, given the class, simply by multiplying the probabilities of observing the individual features, given that class:

$$P(F_1, F_2, \dots, F_{10}|C_1) = P(F_1|C_1)P(F_2|C_1) \dots P(F_{10}|C_1) = \prod_{i=1}^{10} P(F_i|C_1)$$

This quantity (the probability of the observed feature values, given the class) is referred to as the "likelihood". How the individual probabilities are computed depends on the hypotheses about the investigated system. In our case we assume that each of the 10 features has a typical range of values specific to each class (for instance, the ratio for edit=0 is > 1 for pestis, and <1 for the other two species). Specifically, we assume that any given feature value is drawn from a normal distribution with mean and standard deviation depending on the class. The probability density for a given feature value for a given class is therefore found as the normal probability density using the mean and standard deviation for that feature and class. As an example, the probability density of observing the read mapping ratio 1.3 for edit distance = 0 for the class *Y. pestis*, is the following in our model:

$$P(F_1 = 1.3|pestis) = f_{normal}(x = 1.3|\mu = 1.25, \sigma = 0.057) = 4.76$$

The means and standard deviations are parameters in our model, and can be estimated simply by computing means and standard deviations from known examples ("training data" – in our case the data used also in Figure S2B-C these are maximum likelihood estimates of the parameters). Note that the independence assumption also means that it is possible to estimate parameters in the model from much smaller data sets than if features were not taken to be

independent (one just needs sufficient training examples to estimate parameters for each feature individually, instead of examples from all possible combinations of all features).

The second main idea in naïve Bayesian classification is to use Bayes theorem to compute the posterior probability of the possible classes, given the observed feature vector. As an example, the posterior probability for class 1 is computed as follows:

$$P(C_1|\boldsymbol{F}) = \frac{P(\boldsymbol{F}|C_1)P(C_1)}{P(\boldsymbol{F})}$$

Here, $\boldsymbol{F}$ is the entire feature vector (containing 10 values in our case) and the likelihood $P(\boldsymbol{F}|C_1)$ is calculated assuming independence of features as shown above. $P(C_1)$ is known as the prior probability of the class. In the present case we simply used a flat prior distribution, with the same prior probability for all three classes. $P(C_1|\boldsymbol{F})$ is the posterior probability of the class, and quantifies our degree of belief in this class after seeing the data. Finally, $P(\boldsymbol{F})$ is known as the "evidence" and can be seen as a normalizing factor, ensuring that the posterior class probabilities will sum to one. $P(\boldsymbol{F})$ is computed as the sum of the probabilities for the three possible ways of getting the observed features:

$$P(\boldsymbol{F}) = P(\boldsymbol{F}|C_1)P(C_1) + P(\boldsymbol{F}|C_2)P(C_2) + P(\boldsymbol{F}|C_3)P(C_3)$$

As mentioned, we estimated means and standard deviations for each of the 10 features, for each of the 3 classes, from a set of known samples mapped against the *Y. pestis* and *Y. pseudotuberculosis* reference genomes. It turned out that the data available to estimate parameters for *Y. similis* displayed what we judged to be unrealistically little diversity, and we therefore estimated the standard deviations for this class by taking the average of the corresponding standard deviations estimated for *Y. pestis* and *Y. pseudotuberculosis*. (This approach, where parameter values from other groups are used to help regularize the estimate for a group with limited data, is known as shrinkage).

Analysis of sequencing depth, expected coverage, and actual coverage

Sequencing reads are not distributed evenly across a sequenced genome - some positions are covered by more than the average number of reads and others by less. Consequently, coverage (the fraction of positions covered by at least one read) is not necessarily 100% even when the sequencing depth (the average number of reads covering any given position) is well above 1. It is possible to compute the expected coverage based on the distribution of read lengths, under the assumption that read locations have been drawn randomly from the entire

genome (see below). We here use the comparison of actual and expected coverage computed in this manner, as yet another way to assess the authenticity of the analyzed reads. The idea is that if mapped reads do in fact originate from *Y. pestis*, then their locations will be close to randomly distributed across the reference genome, and expected coverage should therefore match actual coverage well. If, on the other hand, the reads do not belong to *Y. pestis*, then their mapped locations on the reference genome are more likely to be biased, for instance with over-representation in regions of low complexity, or perhaps in regions that have been more highly conserved through evolution. In that case, the match between actual and expected coverage should be worse.

Assuming that all reads have exactly the same length the expected coverage can be computed using the following expression: $c = 1 - \left(1 - \frac{l}{g}\right)^r$, where l=read length, g=genome length, and r=number of reads. The rationale is as follows: The probability that any given position in the reference genome will be covered by a read is $\frac{l}{g}$. The probability a position will *not* be covered by a single read is therefore $1 - \frac{l}{g}$. The probability that any given position will *not* be covered after r reads have been placed randomly and independently is therefore $(1 - \frac{l}{g})^r$. The probability that a given read *is* in fact covered after placing r reads, is 1 minus the probability that it is not covered, i.e., $1 - \left(1 - \frac{l}{g}\right)^r$. Since the expected fraction of covered sites, is the same as the probability that any given site is covered, this will also be the expected coverage, c.

Based on the expression above, it is fairly simple to compute the expected coverage also in the event that all reads do not have the same length. If, for instance there are r_l reads of length l_1, and r_2 reads of length l_2, then the expected coverage is simply: $c = 1 - \left(1 - \frac{l_1}{g}\right)^{r_1} \left(1 - \frac{l_2}{g}\right)^{r_2}$. More generally, if the reads have N different lengths, l_l to l_N, with counts r_l to r_N, then the expected coverage is:

$$c = 1 - \prod_{i=1}^{N} \left(1 - \frac{l_i}{g}\right)^{r_i}$$

Even if the location of reads are in fact randomly sampled from the reference genome, there are still two major reasons why an expected coverage, computed according to this equation, may not correspond to the actual coverage. First, if the reference genome contains repeats with a length longer than the read length, then it will not be possible to uniquely map reads corresponding to these repeats. The expected coverage will therefore only refer to the

mappable part of the reference sequence. For each reference sequence (the *Y. pestis* genome and the three associated plasmids), we, for each sample, determined the mappable fraction using k-mer lengths similar to the average read lengths in that sample. Specifically, we determined the mappable fraction for each reference sequence using kmers of length 40, 50, and 60, and then used the mappability value with the k-mer length closest to the actual average read length for each sample/reference combination. The expected coverage, accounting for mappability, is then computed by multiplying the expected coverage by the fraction of the reference sequence that is mappable: $c_{map} = f_{map}c$. The second reason why expected coverage may differ from actual coverage, is if the reference sequence contains regions that are not present in the sequenced sample. We found this to be the case for the pMT1 plasmid, which, for 6 of the investigated samples compared to the reference sequence, was found to lack a 19 kb region harboring the *ymt* gene important for pathogenicity. Again, this can be accounted for by multiplying the expected coverage by the fraction of the reference sequence that is present: $c_{map,del} = f_{del}f_{map}c$. In the case of pMT1, samples lacking this 19 kb region were clearly seen in plots of expected vs actual coverage as being placed well below the line corresponding to perfect correlation.

Figure 3 shows plots of actual vs. expected coverage computed for all samples for the chromosome and the plasmid sequences, using the equations above (and thus accouting for mappability and for the lacking region in some pMT1). It can be seen that expected coverage computed for the reads corresponding to assumed *Y. pestis* fit very well to the actually observed values. The majority of reads not assumed to be *Y. pestis* have very low read counts mapping to the reference sequences, and are seen as a cloud of points in the lower left corners of the plots. A few samples can be seen to have a high count of reads mapped to the *Y. pestis* reference chromosome, and therefore also high expected coverage, but much lower actual coverage, and are therefore most likely not *Y. pestis*. Included among these is the sample RISE392 (shown as red dots in the plots), which was also deemed not to be *Y. pestis* based on the distribution of edit distances.

Genotyping for phylogenetic analyses

The calls were generated from alignments versus *Y. pseudotuberculosis* IP32953 using samtools-0.1.18 and bcftools-0.1.17 (Li et al., 2009). The genotype calls were filtered by removing heterozygote variants, indels and variants that clustered within 10bp of each other, as well as variants within 10 bp of a gap. Additionally genotype calls in modern samples were required to have at least 10 high quality base calls (given by DP4) and ancient samples to have at least 4 high quality base calls per site. To create full-length consensus sequences for

each sample the missing sites in the VCF files were then filled with N basecalls and converted to fasta.

Heterozygosity estimates

To estimate if the RISE505 or RISE509 strains represented an infection with two different *Y. pestis* strains we determined the number of heterozygote sites in the genomes of RISE505, RISE509, the Black Death strain (Bos et al., 2011) and the strains from Cui et al. (Cui et al., 2013). The rationale for this is that heterozygote genotype calls for haploid organisms are normally caused by mapping errors, but in the case of a mixed infection will be caused by divergence between the strains. To allow for comparison between the samples we sampled all the bam-files to the same average depth as RISE505 (8.7X) and RISE509 (29.4X) using samtools (Li et al., 2009). We excluded the Justinian strain (Wagner et al., 2014) from the analysis due to the low average depth across the chromosome (4.3X). Hereafter, we genotyped each of the individuals based on the *Y. pestis* CO92 chromosome and extracted heterozygote genotype calls with a depth equal to or larger than 10 (base quality >= 13). We removed all transitions, as these are typically patterns of DNA damage, and only kept transversions.

Analysis of virulence associated genes

The 55 genes (Black et al., 2000; Blaylock et al., 2010; Burghout et al., 2004; Bzymek et al., 2012; Cheng and Schneewind, 2000; Cornelis, 2002; Day and Plano, 2000; Day et al., 2000; Diepold et al., 2011; Du et al., 2002; Felek et al., 2010; Fields et al., 1999; Fowler et al., 2009; Haddix and Straley, 1992; Håkansson et al., 1996; Hinnebusch et al., 1996, 2002; Huang and Lindler, 2004; Iriarte and Cornelis, 1999; Juris et al., 2000; Kerschen et al., 2004; Li et al., 2014; Lindler et al., 1990; Mukherjee et al., 2006; Payne and Straley, 1998; Perry and Fetherston, 1997; Plano et al., 1991; Ramamurthi and Schneewind, 2003; Rosqvist et al., 1994; Rouvroit et al., 1992; Silva-Herzog et al., 2008; Sodeinde et al., 1992; Stainier et al., 2000; Williams and Straley, 1998; Woestyn et al., 1994) that we identified as associated with virulence of *Y. pestis* are shown in Figure 5 as well as listed in Table S6. For identification of the DFR4 region we used the location of 1,041kb to 1,063kb in the *Y. pestis microtus* 91001 genome. The mappability of the DFR4 region was calculated using GEM-mappability library (Derrien et al., 2012) with a k-mer of 50 using the entire genome as input.

Genotyping of *pde2*, *pde3* and *rcsA* involved in survival in flea gut

We investigated the loss of function mutations in three genes (*pde2*, *pde3* and *rcsA*) which lead to an *Y. pestis* phenotype that causes blockage of the flea gut and thereby increased

probability of transmission (Sun et al., 2014). The loss of function mutations for the genes are a frameshift mutation (6As -> 7As) in the *pde2* gene, a C->T mutation in the promoter and a nonsense point mutation in the *pde3* gene, and a 30bp internal duplication in the *rcsA* gene.

For *pde2* we used the genotypes of RISE509 that were called using the *Y. pseudotuberculosis* IP32953 genome and we did not find any evidence for an insertion which is in concordance with the 6A genotype (position 1,560,134). Likewise when investigating the genotypes based on the *Y. pestis* CO92 genome we find a deletion corresponding to the 6A genotype (position 1,434,043). For the RISE505 sample the *pde2* positions had low coverage (1-2 reads only) and we were unable to determine the genotype.

For *pde3* we investigated both the promoter mutation (IP32953: C -> T at 3,944,166) and the nonsense mutation (IP32953: G -> A at 3,944,534) in RISE509. Although the promoter mutation is a C-T mutation and therefore likely to be confounded by DNA damage, we found 6 non-damaged (not rescaled by MapDamage2) high quality bases confirming the mutation. Likewise, the G-A nonsense mutation is also likely to be masked by DNA damage, but we identified 62 reads in support of G versus only one read in support of A. Likewise as for *pde2*, the RISE505 sample had low read support but still supported the same genotypes as identified in RISE509.

Because *rcsA* is a 30bp internal duplication we performed *de novo* assembly of the RISE509 data using SPAdes as described above. We identified the contig spanning the region and performed multiple alignment using ClustalX (Larkin et al., 2007) (Figure S6). The *de novo* assembled contig did not have the internal duplication and RISE509 therefore has the ancestral form of *rcsA*.

Genotyping *pla* mutations

We identified a novel non-synonymous C to G mutation in amino acid 31 (amino acid 51 in the CO92 reference sequence, position 6,815 on pPCP1) replacing an isoleucine with a valine. We found the mutation to be supported by 55 reads in RISE505 (1746-1626 cal BC) and 46 reads in RISE509 (2815-2677 cal BC), respectively. All other *Y. pestis* genomes, including RISE397 (1048-885 cal BC) carried the derived isoleucine allele (supported by 7 reads).

We additionally investigated the isoleucine 259 to threonine mutation (279 in the CO92 reference sequence, position 7,500 on pPCP1) (Zimbler et al., 2015). However, because the genotype of CO92 at this position is a C and the ancestral state is a T, the genotyping can be confounded by ancient DNA damage. For each of the RISE397, RISE505 and RISE509

samples, the non-damaged bases (not rescaled by MapDamage2) at this site were all supporting the ancestral allele (T) with 3, 2 and 2 reads respectively. We additionally called genotypes for RISE397, RISE505 and RISE509 based on the *Y. pestis microtus* 91001 *pla* gene which contains the ancestral T allele. Here the ancestral allele was supported by 11, 19 and 6 reads, respectively.

Genotyping the *flhD* gene

All *Y. pestis* strains sequenced prior to this study have an insertion of a T in the *flhD* gene (CO92 position: 1,892,659) that is a regulatory gene involved in flagella synthesis (Minnich and Rohde, 2007). When investigating RISE505 and RISE509 for this insertion, we found them to harbor the ancestral and functional *flhD* allele supported by 16 and 29 high quality bases, respectively. The downstream deleted base (in the CO92 genome) was not supported by any high quality reads in any of the two samples.

Supplemental References

Aul, J. (1935). Étude anthropologique des ossements humains néolithiques de Sope. Õpetatud Eesti Seltsi Aastaraam. 1933 224–282.

Batzilla, J., Höper, D., Antonenka, U., Heesemann, J., and Rakin, A. (2011). Complete genome sequence of Yersinia enterocolitica subsp. palearctica serogroup O:3. J. Bacteriol. *193*, 2067.

Black, D.S., Marie-Cardine, A., Schraven, B., and Bliska, J.B. (2000). The Yersinia tyrosine phosphatase YopH targets a novel adhesion-regulated signalling complex in macrophages. Cell. Microbiol. *2*, 401–414.

Blaylock, B., Berube, B.J., and Schneewind, O. (2010). YopR impacts type III needle polymerization in Yersinia species. Mol. Microbiol. *75*, 221–229.

Burghout, P., Beckers, F., de Wit, E., van Boxtel, R., Cornelis, G.R., Tommassen, J., and Koster, M. (2004). Role of the pilot protein YscW in the biogenesis of the YscC secretin in Yersinia enterocolitica. J. Bacteriol. *186*, 5366–5375.

Bzymek, K.P., Hamaoka, B.Y., and Ghosh, P. (2012). Two translation products of Yersinia yscQ assemble to form a complex essential to type III secretion. Biochemistry *51*, 1669–1677.

Chain, P.S.G., Hu, P., Malfatti, S.A., Radnedge, L., Larimer, F., Vergez, L.M., Worsham, P., Chu, M.C., and Andersen, G.L. (2006). Complete genome sequence of Yersinia pestis strains Antiqua and Nepal516: evidence of gene reduction in an emerging pathogen. J. Bacteriol. *188*, 4453–4463.

Cheng, L.W., and Schneewind, O. (2000). Yersinia enterocolitica TyeA, an intracellular regulator of the type III machinery, is required for specific targeting of YopE, YopH, YopM, and YopN into the cytosol of eukaryotic cells. J. Bacteriol. *182*, 3183–3190.

Cholewa, P. (1998). Osady neolityczne na stanowisku nr 1 w Chociwelu, gm: Strzelin. Neolithic settlements in site 1 in Chociwel, near Strzelin. Wrocław Wydaw. Uniw. Wrocławskiego. *30*, 81–168.

Cornelis, G.R. (2002). The Yersinia Ysc-Yop “type III” weaponry. Nat. Rev. Mol. Cell Biol. *3*, 742–752.

Day, J.B., and Plano, G. V (2000). The Yersinia pestis YscY protein directly binds YscX, a secreted component of the type III secretion machinery. J. Bacteriol. *182*, 1834–1843.

Day, J.B., Guller, I., and Plano, G. V (2000). Yersinia pestis YscG protein is a Syc-like chaperone that directly binds yscE. Infect. Immun. *68*, 6466–6471.

Deagle, B.E., Eveson, J.P., and Jarman, S.N. (2006). Quantification of damage in DNA recovered from highly degraded samples--a case study on DNA in faeces. Front. Zool. *3*, 11.

Deng, W., Burland, V., Plunkett, G., Boutin, A., Mayhew, G.F., Liss, P., Perna, N.T., Rose, D.J., Mau, B., Zhou, S., et al. (2002). Genome Sequence of Yersinia pestis KIM. J. Bacteriol. *184*, 4601–4611.

Derrien, T., Estellé, J., Marco Sola, S., Knowles, D.G., Raineri, E., Guigó, R., and Ribeca, P. (2012). Fast computation and applications of genome mappability. PLoS One *7*, e30377.

Diepold, A., Wiesand, U., and Cornelis, G.R. (2011). The assembly of the export apparatus (YscR,S,T,U,V) of the Yersinia type III secretion apparatus occurs independently of other structural components and involves the formation of an YscV oligomer. Mol. Microbiol. *82*, 502–514.

Du, Y., Rosqvist, R., and Forsberg, A. (2002). Role of fraction 1 antigen of Yersinia pestis in inhibition of phagocytosis. Infect. Immun. *70*, 1453–1460.

Eppinger, M., Rosovitz, M.J., Fricke, W.F., Rasko, D.A., Kokorina, G., Fayolle, C., Lindler, L.E., Carniel, E., and Ravel, J. (2007). The complete genome sequence of Yersinia pseudotuberculosis IP31758, the causative agent of Far East scarlet-like fever. PLoS Genet. *3*, e142.

Eppinger, M., Guo, Z., Sebastian, Y., Song, Y., Lindler, L.E., Yang, R., and Ravel, J. (2009). Draft genome sequences of Yersinia pestis isolates from natural foci of endemic plague in China. J. Bacteriol. *191*, 7628–7629.

Eppinger, M., Worsham, P.L., Nikolich, M.P., Riley, D.R., Sebastian, Y., Mou, S., Achtman, M., Lindler, L.E., and Ravel, J. (2010). Genome sequence of the deep-rooted Yersinia pestis strain Angola reveals new insights into the evolution and pangenome of the plague bacterium. J. Bacteriol. *192*, 1685–1699.

Felek, S., Muszyński, A., Carlson, R.W., Tsang, T.M., Hinnebusch, B.J., and Krukonis, E.S. (2010). Phosphoglucomutase of Yersinia pestis is required for autoaggregation and polymyxin B resistance. Infect. Immun. *78*, 1163–1175.

Fields, K.A., Nilles, M.L., Cowan, C., and Straley, S.C. (1999). Virulence Role of V Antigen of Yersinia pestis at the Bacterial Surface. Infect. Immun. *67*, 5395–5408.

Fowler, J.M., Wulff, C.R., Straley, S.C., and Brubaker, R.R. (2009). Growth of calcium-blind mutants of Yersinia pestis at 37 degrees C in permissive Ca2+-deficient environments. Microbiology *155*, 2509–2521.

Haddix, P.L., and Straley, S.C. (1992). Structure and regulation of the Yersinia pestis yscBCDEF operon. J. Bacteriol. *174*, 4820–4828.

Håkansson, S., Schesser, K., Persson, C., Galyov, E.E., Rosqvist, R., Homblé, F., and Wolf-Watz, H. (1996). The YopB protein of Yersinia pseudotuberculosis is essential for the translocation of Yop effector proteins across the target cell plasma membrane and displays a contact-dependent membrane disrupting activity. EMBO J. *15*, 5812–5823.

Hand, D.J., and Yu, K. (2001). Idiot's Bayes? Not So Stupid After All? Int. Stat. Rev. *69*, 385–398.

Hinnebusch, B.J., Perry, R.D., and Schwan, T.G. (1996). Role of the Yersinia pestis hemin storage (hms) locus in the transmission of plague by fleas. Science *273*, 367–370.

Huang, X.-Z., and Lindler, L.E. (2004). The pH 6 antigen is an antiphagocytic factor produced by Yersinia pestis independent of Yersinia outer proteins and capsule antigen. Infect. Immun. *72*, 7212–7219.

Huang, W., Li, L., Myers, J.R., and Marth, G.T. (2012). ART: a next-generation sequencing read simulator. Bioinformatics *28*, 593–594.

Indreko, R. (1935). Sépultures néolithiques en Estonie. Õpetatud Eesti Seltsi Aastaraam. 1933 202–223.

Iriarte, M., and Cornelis, G.R. (1999). Identification of SycN, YscX, and YscY, three new elements of the Yersinia yop virulon. J. Bacteriol. *181*, 675–680.

Jonuks, T. (2009). Eesti muinasusund. Dissertationes archaeologiae universitatis Tartuensis 2. Tartu.

Juris, S.J., Rudolph, A.E., Huddler, D., Orth, K., and Dixon, J.E. (2000). A distinctive role for the Yersinia protein kinase: actin binding, kinase activation, and cytoskeleton disruption. Proc. Natl. Acad. Sci. U. S. A. *97*, 9431–9436.

Kerschen, E.J., Cohen, D.A., Kaplan, A.M., and Straley, S.C. (2004). The plague virulence protein YopM targets the innate immune response by causing a global depletion of NK cells. Infect. Immun. *72*, 4589–4602.

Khalyapin, M. V. (2001). The first cemetery of the Sintashta Culture. In The Bronze Age in Eastern Europe: Characteristics of Cultures, the Chronology and Periodization, Y.I. Kolev, ed. (Samara: NTTZ), pp. 417–425.

Kriiska, A., Lõugas, L., Lõhmus, M., Mannermaa, K., and Johanson, K. (2007). New AMS dates from Estonian Stone Age burial sites. Est. J. Archaeol. *11*, 83–121.

Larkin, M.A., Blackshields, G., Brown, N.P., Chenna, R., McGettigan, P.A., McWilliam, H., Valentin, F., Wallace, I.M., Wilm, A., Lopez, R., et al. (2007). Clustal W and Clustal X version 2.0. Bioinformatics *23*, 2947–2948.

Lasak, I. (1996). Obiekty kultury unietyckiej z Chociwela, woj. wrocławskie. Unětice culture objects from Chociwel, province of Wrocław. Archeologiczne *37*.

Li, Y., Li, L., Huang, L., Francis, M.S., Hu, Y., and Chen, S. (2014). Yersinia Ysc-Yop type III secretion feedback inhibition is relieved through YscV-dependent recognition and secretion of LcrQ. Mol. Microbiol. *91*, 494–507.

Lindler, L.E., Klempner, M.S., and Straley, S.C. (1990). Yersinia pestis pH 6 antigen: genetic, biochemical, and virulence characterization of a protein involved in the pathogenesis of bubonic plague. Infect. Immun. *58*, 2569–2577.

Lõugas, L., Kriiska, A., and Maldre, L. (2007). New dates for the Late Neolithic Corded Ware Culture burials and early husbandry in the East Baltic region. Archaeofauna *16*, 21–31.

Moora, H. (1932). Die Vorzeit Estlands (Tartu).

Mukherjee, S., Keitany, G., Li, Y., Wang, Y., Ball, H.L., Goldsmith, E.J., and Orth, K. (2006). Yersinia YopJ acetylates and inhibits kinase activation by blocking phosphorylation. Science *312*, 1211–1214.

Olalde, I., Allentoft, M.E., Sánchez-Quinto, F., Santpere, G., Chiang, C.W.K., DeGiorgio, M., Prado-Martinez, J., Rodríguez, J.A., Rasmussen, S., Quilez, J., et al. (2014). Derived immune

and ancestral pigmentation alleles in a 7,000-year-old Mesolithic European. Nature *507*, 225–228.

Orlando, L., Ginolhac, A., Zhang, G., Froese, D., Albrechtsen, A., Stiller, M., Schubert, M., Cappellini, E., Petersen, B., Moltke, I., et al. (2013). Recalibrating Equus evolution using the genome sequence of an early Middle Pleistocene horse. Nature *499*, 74–78.

Payne, P.L., and Straley, S.C. (1998). YscO of Yersinia pestis is a mobile core component of the Yop secretion system. J. Bacteriol. *180*, 3882–3890.

Pedersen, J.S., Valen, E., Velazquez, A.M.V., Parker, B.J., Rasmussen, M., Lindgreen, S., Lilje, B., Tobin, D.J., Kelly, T.K., Vang, S., et al. (2014). Genome-wide nucleosome map and cytosine methylation levels of an ancient human genome. Genome Res. *24*, 454–466.

Plano, G. V, Barve, S.S., and Straley, S.C. (1991). LcrD, a membrane-bound regulator of the Yersinia pestis low-calcium response. J. Bacteriol. *173*, 7293–7303.

Pokutta, D.A. (2013). Population Dynamics, Diet and Migrations of the Unetice Culture in Poland. University of Gothenburg.

Ramamurthi, K.S., and Schneewind, O. (2003). Yersinia yopQ mRNA encodes a bipartite type III secretion signal in the first 15 codons. Mol. Microbiol. *50*, 1189–1198.

Reuter, S., Connor, T.R., Barquist, L., Walker, D., Feltwell, T., Harris, S.R., Fookes, M., Hall, M.E., Petty, N.K., Fuchs, T.M., et al. (2014). Parallel independent evolution of pathogenicity within the genus Yersinia. Proc. Natl. Acad. Sci. U. S. A. *111*, 6768–6773.

Rosqvist, R., Magnusson, K.E., and Wolf-Watz, H. (1994). Target cell contact triggers expression and polarized transfer of Yersinia YopE cytotoxin into mammalian cells. EMBO J. *13*, 964–972.

Rouvroit, C., Sluiters, C., and Cornelis, G. (1992). Role of the transcriptional activator, VirF, and temperature in the expression of the pYV plasmid genes of Yersinia enterocolitica. Mol. Microbiol. *6*, 395–409.

Shen, X., Wang, Q., Xia, L., Zhu, X., Zhang, Z., Liang, Y., Cai, H., Zhang, E., Wei, J., Chen, C., et al. (2010). Complete genome sequences of Yersinia pestis from natural foci in China. J. Bacteriol. *192*, 3551–3552.

Silva-Herzog, E., Ferracci, F., Jackson, M.W., Joseph, S.S., and Plano, G. V (2008). Membrane localization and topology of the Yersinia pestis YscJ lipoprotein. Microbiology *154*, 593–607.

Song, Y., Tong, Z., Wang, J., Wang, L., Guo, Z., Han, Y., Zhang, J., Pei, D., Zhou, D., Qin, H., et al. (2004). Complete genome sequence of Yersinia pestis strain 91001, an isolate avirulent to humans. DNA Res. *11*, 179–197.

Stainier, I., Bleves, S., Josenhans, C., Karmani, L., Kerbourch, C., Lambermont, I., Tötemeyer, S., Boyd, A., and Cornelis, G.R. (2000). YscP, a Yersinia protein required for Yop secretion that is surface exposed, and released in low Ca2+. Mol. Microbiol. *37*, 1005–1018.

Thomson, N.R., Howard, S., Wren, B.W., Holden, M.T.G., Crossman, L., Challis, G.L., Churcher, C., Mungall, K., Brooks, K., Chillingworth, T., et al. (2006). The Complete Genome Sequence and Comparative Genome Analysis of the High Pathogenicity Yersinia enterocolitica Strain 8081. PLoS Genet. *2*, e206.

Umanski, A., Kiryushin, Y., and Grushin, S. (2007). The burial traditions of the Andronovo people of Chumysh area (based on Kytmanovo data) (Barnaul: Altai University Press).

Vadetskaya, E., Polyakov, A., and Stepanova, N. (2014). The set sites of the Afanasievo culture (Barnaul: Azbuka).

Wang, X., Li, Y., Jing, H., Ren, Y., Zhou, Z., Wang, S., Kan, B., Xu, J., and Wang, L. (2011). Complete genome sequence of a Yersinia enterocolitica "Old World" (3/O:9) strain and comparison with the "New World" (1B/O:8) strain. J. Clin. Microbiol. *49*, 1251–1259.

Williams, A.W., and Straley, S.C. (1998). YopD of Yersinia pestis Plays a Role in Negative Regulation of the Low-Calcium Response in Addition to Its Role in Translocation of Yops. J. Bacteriol. *180*, 350–358.

Woestyn, S., Allaoui, A., Wattiau, P., and Cornelis, G.R. (1994). YscN, the putative energizer of the Yersinia Yop secretion machinery. J. Bacteriol. *176*, 1561–1569.

Zhang, H. (2004). The optimality of naive Bayes. AA *1*, 3.

Zhang, Z., Hai, R., Song, Z., Xia, L., Liang, Y., Cai, H., Shen, X., Zhang, E., Xu, J., Yu, D., et al. (2009). Spatial Variation of Yersinia pestis from Yunnan Province of China. Am. J. Trop. Med. Hyg. *81*, 714–717.
