# Supplementary material for: Early Divergent Strains of Yersinia pestis in Eurasia 5,000 Years Ago
Source: Cell. 2015 Oct 22;163(3):571–82. doi: 10.1016/j.cell.2015.10.009 (PMC4644222; doi:10.1016/j.cell.2015.10.009)
Supplement: Document S3. Article plus Supplemental Information [file mmc3.pdf]

Cell

Early Divergent Strains of *Yersinia pestis* in Eurasia 5,000 Years Ago

Graphical Abstract

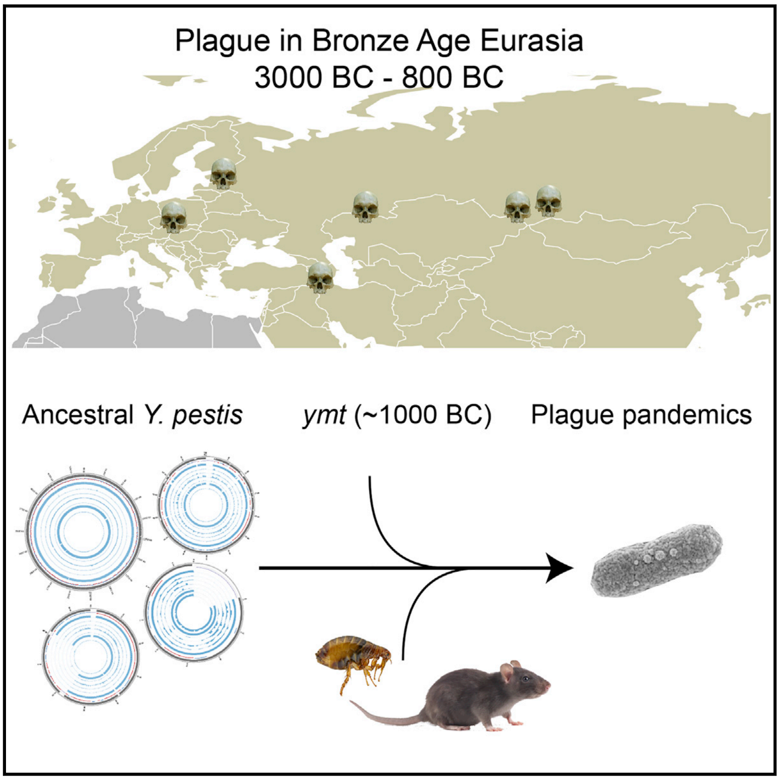

Authors

Simon Rasmussen, Morten Erik Allentoft, Kasper Nielsen, ..., Rasmus Nielsen, Kristian Kristiansen, Eske Willerslev

Correspondence

ewillerslev@snm.ku.dk

In Brief

The plague-causing bacteria *Yersinia pestis* infected humans in Bronze Age Eurasia, three millennia earlier than any historical records of plague, but only acquired the genetic changes making it a highly virulent, flea-borne bubonic strain ~3,000 years ago.

Highlights

- *Yersinia pestis* was common across Eurasia in the Bronze Age
- The most recent common ancestor of all *Y. pestis* was 5,783 years ago
- The *ymt* gene was acquired before 951 cal BC, giving rise to transmission via fleas
- Bronze Age *Y. pestis* was not capable of causing bubonic plague

Rasmussen et al., 2015, Cell *163*, 571–582
October 22, 2015 ©2015 The Authors
http://dx.doi.org/10.1016/j.cell.2015.10.009

CellPress

Early Divergent Strains of *Yersinia pestis* in Eurasia 5,000 Years Ago

Simon Rasmussen,[1,18] Morten Erik Allentoft,[2,18] Kasper Nielsen,[1] Ludovic Orlando,[2] Martin Sikora,[2] Karl-Göran Sjögren,[3] Anders Gorm Pedersen,[1] Mikkel Schubert,[2] Alex Van Dam,[1] Christian Moliin Outzen Kapel,[4] Henrik Bjørn Nielsen,[1] Søren Brunak,[1,5] Pavel Avetisyan,[6] Andrey Epimakhov,[7] Mikhail Viktorovich Khalyapin,[8] Artak Gnuni,[9] Aivar Kriiska,[10] Irena Lasak,[11] Mait Metspalu,[12] Vyacheslav Moiseyev,[13] Andrei Gromov,[13] Dalia Pokutta,[3] Lehti Saag,[12] Liivi Varul,[10] Levon Yepiskoposyan,[14] Thomas Sicheritz-Pontén,[1] Robert A. Foley,[15] Marta Mirazón Lahr,[15] Rasmus Nielsen,[16] Kristian Kristiansen,[3] and Eske Willerslev[2,17,*]
[1]Center for Biological Sequence Analysis, Department of Systems Biology, Technical University of Denmark, Kemitorvet, Building 208, 2800 Kongens Lyngby, Denmark
[2]Centre for GeoGenetics, Natural History Museum of Denmark, University of Copenhagen, Øster Voldgade 5–7, 1350 Copenhagen, Denmark
[3]Department of Historical Studies, University of Gothenburg, 405 30 Gothenburg, Sweden
[4]Section for Organismal Biology, Department of Plant and Environmental Sciences, University of Copenhagen, Thorvaldsensvej 40, 1871 Frederiksberg C, Denmark
[5]Novo Nordisk Foundation Center for Protein Research, University of Copenhagen, 2200 Copenhagen, Denmark
[6]Division of Armenology and Social Sciences, Institute of Archaeology and Ethnography, National Academy of Sciences, 0025 Yerevan, Republic of Armenia
[7]Institute of History and Archaeology RAS (South Ural Department), South Ural State University, 454080 Chelyabinsk, Russia
[8]Orenburg Museum of Fine Arts, 460000 Orenburg, Russia
[9]Department of Archaeology and Ethnography, Yerevan State University, 0025 Yerevan, Republic of Armenia
[10]Department of Archaeology, University of Tartu, 51003 Tartu, Estonia
[11]Institute of Archaeology, University of Wrocław, 50-139 Wrocław, Poland
[12]Department of Evolutionary Biology, Estonian Biocentre and University of Tartu, 51010 Tartu, Estonia
[13]Peter the Great Museum of Anthropology and Ethnography (Kunstkamera) RAS, 199034 St. Petersburg, Russia
[14]Laboratory of Ethnogenomics, Institute of Molecular Biology, National Academy of Sciences, 0014 Yerevan, Armenia
[15]Leverhulme Centre for Human Evolutionary Studies, Department of Archaeology and Anthropology, University of Cambridge, Cambridge CB2 1QH, UK
[16]Center for Theoretical Evolutionary Genetics, University of California, Berkeley, California 94720-3140, USA
[17]Department of Zoology, University of Cambridge, Downing Street, Cambridge CB2 3EJ, UK
[18]Co-first author
*Correspondence: ewillerslev@snm.ku.dk
http://dx.doi.org/10.1016/j.cell.2015.10.009
This is an open access article under the CC BY license (http://creativecommons.org/licenses/by/4.0/).

SUMMARY

The bacteria *Yersinia pestis* is the etiological agent of plague and has caused human pandemics with millions of deaths in historic times. How and when it originated remains contentious. Here, we report the oldest direct evidence of *Yersinia pestis* identified by ancient DNA in human teeth from Asia and Europe dating from 2,800 to 5,000 years ago. By sequencing the genomes, we find that these ancient plague strains are basal to all known *Yersinia pestis*. We find the origins of the *Yersinia pestis* lineage to be at least two times older than previous estimates. We also identify a temporal sequence of genetic changes that lead to increased virulence and the emergence of the bubonic plague. Our results show that plague infection was endemic in the human populations of Eurasia at least 3,000 years before any historical recordings of pandemics.

INTRODUCTION

Plague is caused by the bacteria *Yersinia pestis* and is being directly transmitted through human-to-human contact (pneumonic plague) or via fleas as a common vector (bubonic or septicemic plague) (Treille and Yersin, 1894). Three historic human plague pandemics have been documented: (1) the First Pandemic, which started with the Plague of Justinian (541–544 AD), but continued intermittently until ~750 AD; (2) the Second Pandemic, which began with the Black Death in Europe (1347–1351 AD) and included successive waves, such as the Great Plague (1665–1666 AD), until the 18th century; (3) the Third Pandemic, which emerged in China in the 1850s and erupted there in a major epidemic in 1894 before spreading across the world as a series of epidemics until the middle of the 20th century (Bos et al., 2011; Cui et al., 2013; Drancourt et al., 1998; Harbeck et al., 2013; Parkhill et al., 2001; Perry and Fetherston, 1997; Wagner et al., 2014). Earlier outbreaks such as the Plague of Athens (430–427 BC) and the Antonine Plague (165–180 AD) may also have occurred, but there is no direct evidence that allows confident attribution to *Y. pestis* (Drancourt and Raoult, 2002; McNeill, 1976).

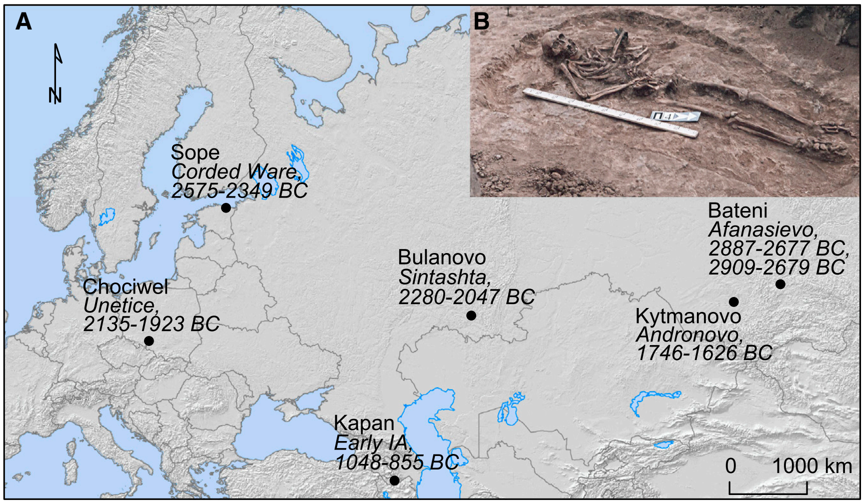

Figure 1. Archaeological Sites of Bronze Age *Yersinia pestis*

(A) Map of Eurasia indicating the position, radiocarbon dated ages and associated cultures of the samples in which *Y. pestis* were identified. Dates are given as 95% confidence interval calendar BC years. IA: Iron Age.

(B) Burial four from Bulanovo site. Picture by Mikhail V. Khalyapin. See also Table S1.

The consequences of the plague pandemics have been well-documented and the demographic impacts were dramatic (Little et al., 2007). The Black Death alone is estimated to have killed 30%–50% of the European population. Economic and political collapses have also been in part attributed to the devastating effects of the plague. The Plague of Justinian is thought to have played a major role in weakening the Byzantine Empire, and the earlier putative plagues have been associated with the decline of Classical Greece and likely undermined the strength of the Roman army.

Molecular clock estimates have suggested that *Y. pestis* diversified from the more prevalent and environmental stress-tolerant, but less pathogenic, enteric bacterium *Y. pseudotuberculosis* between 2,600 and 28,000 years ago (Achtman et al., 1999, 2004; Cui et al., 2013; Wagner et al., 2014). However, humans may potentially have been exposed to *Y. pestis* for much longer than the historical record suggests, though direct molecular evidence for *Y. pestis* has not been obtained from skeletal material older than 1,500 years (Bos et al., 2011; Wagner et al., 2014). The most basal strains of *Y. pestis* (0.PE7 clade) recorded to date were isolated from the Qinghai-Tibet Plateau in China in 1961–1962 (Cui et al., 2013).

We investigated the origin of *Y. pestis* by sequencing ancient bacterial genomes from the teeth of Bronze Age humans across Europe and Asia. Our findings suggest that the virulent, flea-borne *Y. pestis* strain that caused the historic bubonic plague pandemics evolved from a less pathogenic *Y. pestis* lineage infecting human populations long before recorded evidence of plague outbreaks.

RESULTS

Identification of *Yersinia pestis* in Bronze Age Eurasian Individuals

We screened c. 89 billion raw DNA sequence reads obtained from teeth of 101 Bronze Age individuals from Europe and Asia (Allentoft et al., 2015) and found that seven individuals carried sequences resembling *Y. pestis* (Figure 1, Table S1, Supplemental Experimental Procedures). Further sequencing allowed us to assemble the *Y. pestis* genomes to an average depth of 0.14–29.5X, with 12%–95% of the positions in the genome covered at least once (Table 1, Table S2, S3, and S4). We also recovered the sequences of the three plasmids pCD1, pMT1, and pPCP1 (0.12 to 50.3X in average depth) the latter two of which are crucial for distinguishing *Y. pestis* from its highly similar ancestor *Y. pseudotuberculosis* (Table 1, Figure 2, Table S3) (Bercovier et al., 1980; Chain et al., 2004; Parkhill et al., 2001). The host individuals from which *Y. pestis* was recovered belong to Eurasian Late Neolithic and Bronze Age cultures (Allentoft et al., 2015), represented by the Afanasievo culture in Altai, Siberia (2782 cal BC, 2794 cal BC, n = 2), the Corded Ware culture in Estonia (2462 cal BC, n = 1), the Sintashta culture in Russia (2163 cal BC, n = 1), the Unetice culture in Poland (2029 cal BC, n = 1), the Andronovo culture in Altai, Siberia (1686 cal BC, n = 1), and an early Iron Age individual from Armenia (951 cal BC, n = 1) (Table S1).

Authentication of *Yersinia pestis* Ancient DNA

Besides applying standard precautions for working with ancient DNA (Willerslev and Cooper, 2005), the authenticity of our findings are supported by the following observations: (1) The *Y. pestis* sequences were identified in significant amounts in shotgun data from eight of 101 samples, showing that this finding is not due to a ubiquitous contaminant in our lab or in the reagents. Indeed, further analysis showed that one of these eight was most likely not *Y. pestis*. We also sequenced all negative DNA extraction controls and found no signs of *Y. pestis* DNA in these (Table S3). (2) Consistent with an ancient origin, the *Y. pestis* reads were highly fragmented, with average read lengths of 43–65 bp (Table S3) and also displayed clear signs of C-T deamination damage at the 5′ termini typical of ancient DNA (Figure 3, Figure S1). Because the plasmids are central for discriminating between *Y. pestis* and *Y. pseudotuberculosis*, we tested separately for DNA damage patterns for the chromosome and for each of the plasmids. For the seven samples, we observe similar patterns of DNA damage for chromosome and plasmid sequences (Figure 3, Figure S1). (3) We observe correlated DNA degradation patterns when comparing DNA degradation in the *Y. pestis* sequences and the human sequences from the host individual. Given that DNA decay can be described as a rate process (Allentoft et al., 2012), this suggests that the DNA molecules of the pathogen and the human host have a similar age (Figure 3, Figure S1, Table S3 and Supplemental

Table 1. Overview of the *Y. pestis* Containing Samples

Sample	Country	Site	Culture	Date (cal BC)	CO92	pMT1	pPCP1	pCD1
RISE00	Estonia	Sope	Corded Ware	2575–2349	0.39	0.36	1.40	0.66
RISE139	Poland	Chociwel	Unetice	2135–1923	0.14	0.24	0.76	0.28
RISE386	Russia	Bulanovo	Sintashta	2280–2047	0.82	0.96	1.12	1.60
RISE397	Armenia	Kapan	EIA	1048–885	0.25	0.40	6.88	0.50
RISE505	Russia	Kytmanovo	Andronovo	1746–1626	8.73	9.15	34.09	17.46
RISE509	Russia	Afanasievo Gora	Afanasievo	2887–2677	29.45	16.96	31.22	50.32
RISE511	Russia	Afanasievo Gora	Afanasievo	2909–2679	0.20	0.24	1.19	0.60

The dating is direct AMS dating of bones and teeth and is given as 95% confidence interval calendar BC years (details are given in Table S1). The columns CO92, pMT1, pPCP1 and pCD1 correspond to sequencing depth. Additional information on the archaeological sites and mapping statistics can be found in the Supplemental Experimental Procedures and Table S1, S2, and S3. EIA: Early Iron Age, AMS: Accelerator Mass Spectrometry.

Experimental Procedures). (4) Because of the high sequence similarity between *Y. pestis* and *Y. pseudotuberculosis*, we mapped all reads both to the *Y. pestis* CO92 and to the *Y. pseudotuberculosis* IP32953 reference genomes (Chain et al., 2004). Consistent with being *Y. pestis*, the seven investigated samples displayed more reads matching perfectly (edit distance = 0) toward *Y. pestis* (Figure 3, Figure S2). One sample (RISE392) was most likely not *Y. pestis* based on this criterion. (5) A naive Bayesian classifier trained on known genomes predicts the seven samples to be *Y. pestis* with 100% posterior probability, while RISE392 is predicted to have 0% probability of being *Y. pestis* (Figure S2, Table S3). (6) If the DNA was from other organisms than *Y. pestis*, we would expect the reads to be more frequently associated with either highly conserved or low-complexity regions. However, we find the reads to be distributed across the entire genome (Figure S2), and comparison of actual coverage versus the coverage that would be expected from read length distributions and mappability of the reference sequences are also in agreement for the seven samples (Figure 3). (7) In a maximum likelihood phylogeny, the recovered *Y. pestis* genomic sequences of RISE505 and RISE509 are clearly within the *Y. pestis* clade and basal to all contemporary *Y. pestis* strains (Figure 4) (see below).

The Phylogenetic Position of the Bronze Age *Yersinia pestis* Strains

To determine the phylogenetic positions of the two high coverage ancient *Y. pestis* strains, RISE505 (Andronovo culture 1686 cal BC, 8.7X) and RISE509 (Afanasievo culture, 2746 cal BC, 29.7X), we mapped the reads, together with reads from strains of *Yersinia similis* (n = 5), *Y. pseudotuberculosis* (n = 25), and *Y. pestis* (n = 139), to the *Y. pseudotuberculosis* reference genome (IP32953). Only high confidence positions were extracted. To assess whether the individuals were infected with multiple strains of *Y. pestis* we investigated the genotype heterozygosity levels of the ancient genomes and found no indications of mixed infection (Figure S3). There was no decay in Linkage Disequilibrium (LD) across the chromosome (Figure S3), indicating no detectable recombination among strains. We therefore used RAxML (Stamatakis, 2014) to construct a Maximum Likelihood phylogeny from a supermatrix concatenated from 3,141 genes and a total of 3.14 Mbp (Figure 4). This contrasts with earlier phylogenies (Bos et al., 2011; Cui et al., 2013; Morelli et al., 2010; Wagner et al., 2014), which were based on less than 2,300 nucleotides that were ascertained to be variable in *Y. pestis*, likely leading to lower statistical accuracy than with whole-genome analyses. Furthermore, the use of SNPs ascertained to be variable in *Y. pestis* would downwardly bias estimates of branch lengths in *Y. pseudotuberculosis* and lead to underestimates of the *Y. pestis* versus *Y pseudotuberculosis* divergence time, as seen in the branch length of the *Y. pestis* clade to *Y. pseudotuberculosis* (Figure S3). The topology of our whole genome tree shows *Y. pestis* as a monophyletic group within *Y. pseudotuberculosis* with RISE505 and RISE509 (Figure 4A, black arrow, Figure S4) clustered together within the *Y. pestis* clade. The *Y. pestis* sub-tree topology (Figure 4B, Figure S4) is similar to that reported previously (Bos et al., 2011; Cui et al., 2013; Morelli et al., 2010; Wagner et al., 2014), but with the two ancient strains (RISE505 and RISE509) falling basal to all other known strains of *Y. pestis* (100% bootstrap support).

Determination of *Yersinia pestis* Divergence Dates

To determine the dates for the most recent common ancestor (MRCA) of *Y. pestis* and *Y. pseudotuberculosis,* and for all known *Y. pestis* strains, we used a Bayesian Markov Chain Monte Carlo approach implemented in BEAST2 (Bouckaert et al., 2014) on a subset of the supermatrix. We estimated the MRCA of *Y. pestis* and *Y. pseudotuberculosis* to be 54,735 years ago (95% HPD [highest posterior density] interval: 34,659–78,803 years ago) (Figure 4C, Figure S5, Table S5), which is about twice as old compared to previous estimates of 2,600–28,000 years ago (Achtman et al., 1999, 2004; Cui et al., 2013; Wagner et al., 2014). Additionally, we estimated the age of the MRCA of all known *Y. pestis* to 5,783 years ago (95% HPD interval: 5,021–7,022 years ago). This is also significantly older and with a much narrower confidence interval than previous findings of 3,337 years ago (1,505–6,409 years ago) (Cui et al., 2013).

Bronze Age *Yersinia pestis* Strains Lacking Yersinia Murine Toxin

For the high-depth ancient *Y. pestis* genomes, we investigated the presence of 55 genes that have been associated with the virulence of *Y. pestis* (Figure 5A, Table S6). We found all virulence genes to be present, except the Yersinia murine toxin (*ymt*) gene that is located at 74.4–76.2 kb on the pMT1 plasmid (Figure 2C, arrow 1). The *ymt* gene encodes a phospholipase D that protects

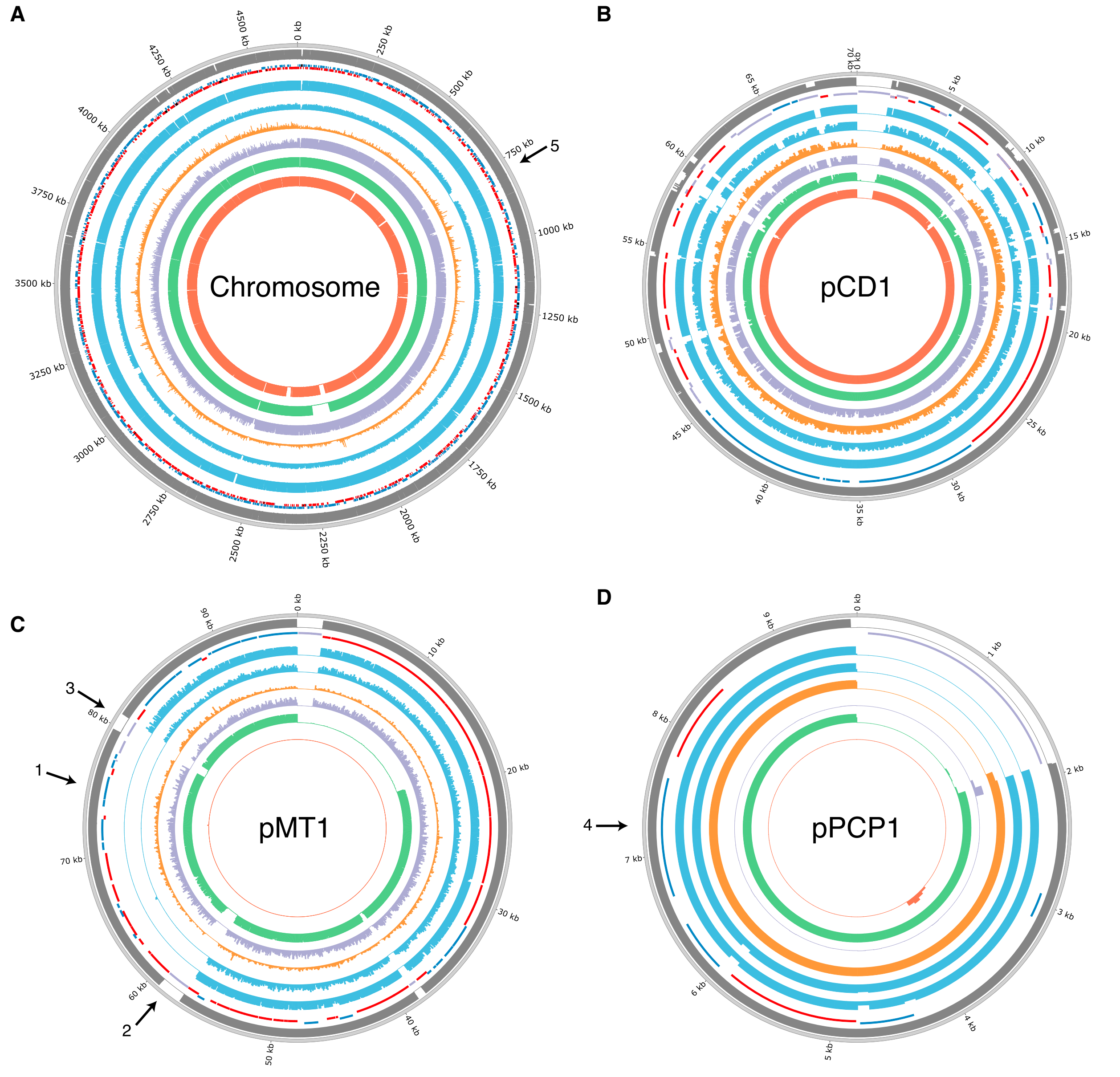

Figure 2. *Y. pestis* Depth of Coverage Plots

(A–D) Depth of coverage plots for (A) CO92 chromosome, (B) pCD1, (C) pMT1, (D) pPCP1. Outer ring: Mappability (gray), genes (RNA: black, transposon: purple, positive strand: blue, negative strand: red), RISE505 (blue), RISE509 (blue), Justinian plague (orange), Black Death plague (purple), modern *Y. pestis* D1982001 (green), *Y. pseudotuberculosis* IP32881 (red) sample. The modern *Y. pestis* and *Y. pseudotuberculosis* samples are included for reference. The histograms show sequence depth in 1 kb windows for the chromosome and 100 bp windows for the plasmids with a max of 20X depth for each ring. Arrow 1: *ymt* gene, arrow 2: transposon at start of missing region on pMT1, arrow 3: transposon at end of missing region on pMT1, arrow 4: *pla* gene, arrow 5: missing flagellin region on chromosome. The plots were generated using Circos (Krzywinski et al., 2009). See also Tables S2, S3 and S8.

Y. pestis inside the flea gut, thus enabling this enteric bacteria to use an arthropod as vector; it further allows for higher titers of *Y. pestis* and higher transmission rates (Hinnebusch, 2005; Hinnebusch et al., 2002). When investigating all seven samples for the presence of *ymt*, we identified a 19 kb region (59–78 kb, Figure 2C arrow 2–3, Figure 5B) to be missing except in the youngest sample (RISE397, 951 cal BC) (Figure 5B, Table S7). We find this region to be present in all other published *Y. pestis* strains (modern and ancient), except three strains (5761, 945, and CA88) that are lacking the pMT1 plasmid completely.

Although larger sample sizes are needed for confirmation, our data indicate that the *ymt* gene was not present in *Y. pestis* before 1686 cal BC (n = 6), while after 951 cal BC, it is found in 97.8% of the strains (n = 140), suggesting a late and very rapid spread of *ymt*. This contrasts with previous studies arguing that the *ymt* gene was acquired early in *Y. pestis* evolution due

to its importance in its life cycle (Carniel, 2003; Hinnebusch, 2005; Hinnebusch et al., 2002; Sun et al., 2014). Interestingly, we identified two transposase elements flanking the missing 19 kb region, confirming that the *ymt* gene was acquired through horizontal gene transfer, as previously suggested (Lindler et al., 1998). Moreover, it has recently been shown that the transmission of *Y. pestis* by fleas is also dependent on loss of function mutations in the *pde2*, *pde3*, and *rcsA* genes (Sun et al., 2014). The RISE509 sample carries the promoter mutation of *pde3* and the functional *pde2* and *rcsA* alleles (Figure S6). In combination with the absence of *ymt*, these results strongly suggest that the ancestral *Y. pestis* bacteria in these early Bronze Age individuals were not transmitted by fleas.

Native Plasminogen Activator Gene Present in Bronze Age *Yersinia pestis*

Another hallmark gene of *Y. pestis* pathogenicity is the plasminogen activator gene *pla* (omptin protein family), located on the pPCP1 plasmid (6.6–7.6 kb). The gene facilitates deep tissue invasion and is essential for development of both bubonic and pneumonic plague (Sebbane et al., 2006; Sodeinde et al., 1992; Zimbler et al., 2015). We identify the gene in six of the seven genomes, but not in RISE139, the sample with the lowest overall depth of coverage (0.75X on pPCP1) (Figure 2D, arrow 4, Table S6). Recently, it has been proposed that pPCP1 was acquired after the branching of the 0.PE2 clade (Zimbler et al., 2015); however, we identified pPCP1 in our samples, including in the 0.PE7 clade (strains 620024 and CMCC05009), which diverged prior to the common ancestor of the 0.PE2 lineage (Figure 4B, Figure 5A). This shows that pPCP1 and *pla* likely were present in the most basal *Y. pestis* (RISE509), suggesting that the 0.PE2 strains lost the pPCP1 plasmid. Interestingly, three 2.ANT3 strains (5761, CMCC64001, and 735) are also missing the *pla* gene, indicating that the loss of pPCP1 occurred more than once in the evolutionary history of *Y. pestis*.

Additionally, we investigated whether RISE397, RISE505, and RISE509 had the isoleucine to threonine mutation at amino acid 259 in the Pla protein. This mutation has been shown to be essential for developing bubonic, but not pneumonic, plague (Zimbler et al., 2015). We found that these samples, in agreement with their basal phylogenetic position, carry the ancestral isoleucine residue. However, we also identified a valine to isoleucine mutation at residue 31 for RISE505 (1686 cal BC) and RISE509 (2746 cal BC). This mutation was not found in any of the other 140 *Y. pestis* strains, but was present in other omptin proteins, such as *Escherichia coli* and *Citrobacter koseri*, and very likely represents the ancestral *Y. pestis* state. The youngest of the samples, RISE397 (951 cal BC) carries the derived isoleucine residue, showing that this mutation, similar to the acquisition of *ymt*, was only observed after 1686 cal BC.

An alternative explanation to the acquisition of *ymt* and the *pla* I259T mutation, given the disparate geographical locations of our samples, could be that the Armenian strain (RISE397, 951 cal BC) containing *ymt* and the isoleucine residue in *pla* had a longer history in the Middle East and experienced an expansion during the 1st millennium BC. This would have led to its export to Eurasia and presumably the extinction of the other more ancestral and less virulent *Y. pestis* strains.

Different Region 4 Present in the Ancestral *Yersinia pestis*

Besides the 55 pathogenicity genes, we also investigated the presence of different region 4 (DFR4) that contains several genes with potential role in *Y. pestis* virulence (Radnedge et al., 2002). This region was reported as present in the Plague of Justinian and Black Death strains, having been lost in the CO92 reference genome (from the Third Pandemic) (Chain et al., 2004; Wagner et al., 2014). Consistent with the ancestral position of our samples, we find evidence that the region is present in all of our seven samples (Figure S6).

Yersinia pestis flagellar Frameshift Mutation Absent in Bronze Age Strains

Another important feature of *Y. pestis* is the ability to evade the mammalian immune system. Flagellin is a potent initiator of the mammalian innate immune system (Hayashi et al., 2001). *Y. pseudotuberculosis* is known to downregulate expression of flagellar systems in a temperature-dependent manner, and none of the known *Y. pestis* strains express flagellin due to a frameshift mutation in the *flhD* regulatory gene (Minnich and Rohde, 2007). However, we do not find this mutation in either RISE505 or RISE509, suggesting that they have fully functional *flhD* genes and that the loss of function occurred after 2746 cal BC. Interestingly, the youngest of these two *Y. pestis* genomes (RISE505, 1686 cal BC) shows partial loss of one of the two flagella systems (758–806 kb), with 39 of 49 genes deleted (Figure 2A, arrow 5, Table S8). This deletion was not found in any of the other *Y. pestis* samples (n = 147). This may point to selective pressure on ancestral *Y. pestis* when emerging as a mammalian pathogen, yielding variably adaptive strains.

DISCUSSION

Our calibrated molecular clock pushes the divergence dates for the early branching of *Y. pestis* back to 5,783 years ago, an additional 2,000 years compared to previous findings (Table S5, Figure S5) (Cui et al., 2013; Morelli et al., 2010). Furthermore, using the temporally stamped ancient DNA data, we are able to derive a time series for the molecular acquisition of the pathogenicity elements and immune avoidance systems that facilitated the evolution from a less virulent bacteria with zoonotic potential, such as *Y. pseudotuberculosis*, to one of the most deadly bacteria ever encountered by humans (Figure 6).

From our findings, we conclude that the ancestor of extant *Y. pestis* strains was present by the end of the 4th millennium BC and was widely spread across Eurasia from at least the early 3rd millennium BC. The occurrence of plague in the Bronze Age Eurasian individuals we sampled (7 of 101) indicates that plague infections were common at least 3,000 years earlier than recorded historically. However, based on the absence of crucial virulence genes, unlike the later *Y. pestis* strains that were responsible for the first to third pandemics, these ancient ancestral *Y. pestis* strains likely did not have the ability to cause bubonic plague, only pneumonic and septicemic plague. These early plagues may have been responsible for the suggested population declines in the late 4th millennium BC and the early 3rd millennium BC (Hinz et al., 2012; Shennan et al., 2013).

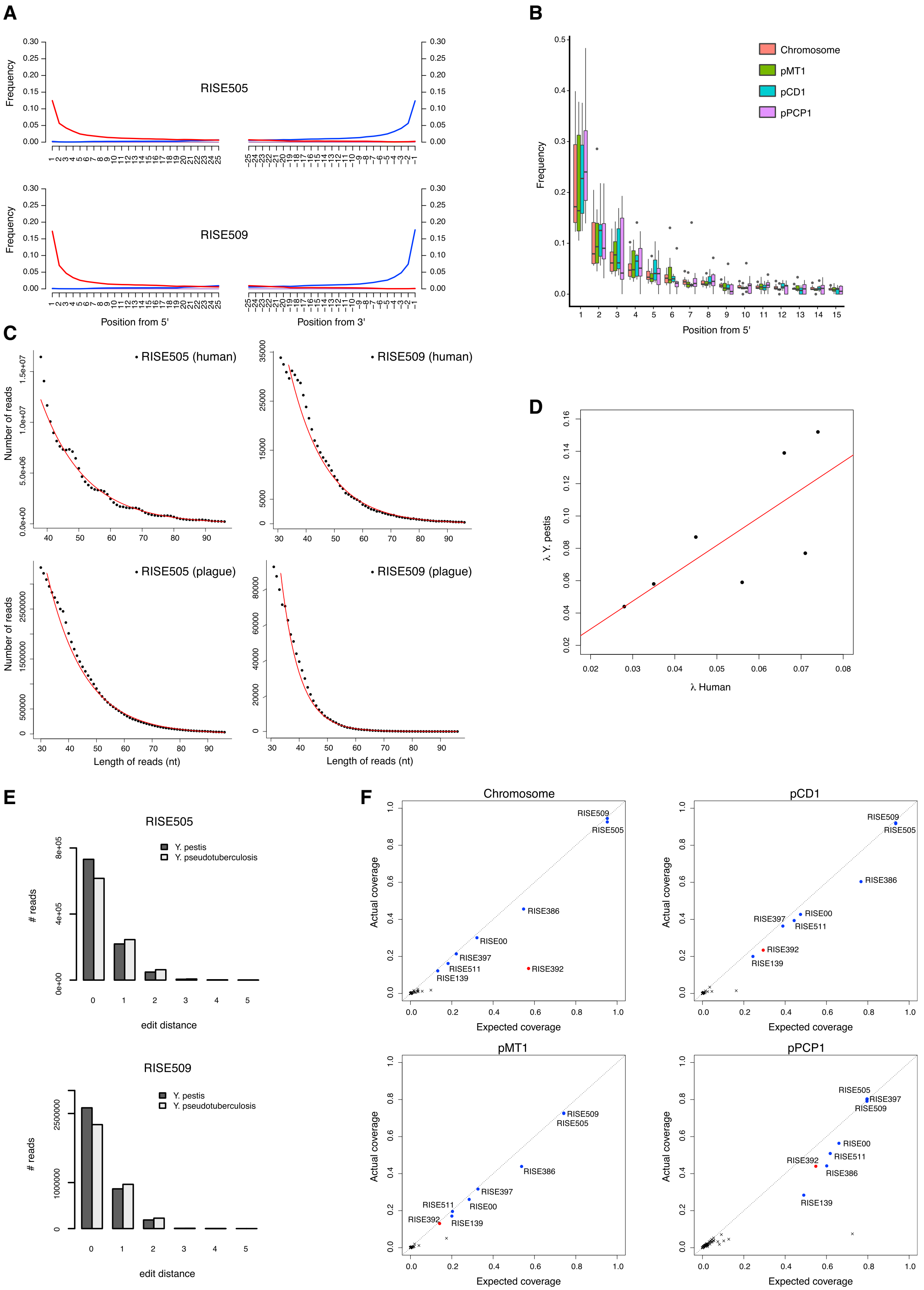

(legend on next page)

It has recently been demonstrated by ancient genomics that the Bronze Age in Europe and Asia was characterized by large-scale population movements, admixture, and replacements (Allentoft et al., 2015; Haak et al., 2015), which accompanied profound and archaeologically well-described social and economic changes (Anthony, 2007; Kristiansen and Larsson, 2005). In light of our findings, it is plausible that plague outbreaks could have facilitated—or have been facilitated by—these highly dynamic demographic events. However, our data suggest that *Y. pestis* did not fully adapt as a flea-borne mammalian pathogen until the beginning of the 1st millennium BC, which precipitated the historically recorded plagues.

EXPERIMENTAL PROCEDURES

Samples and Archaeological Sites

We initially re-analyzed the data from Allentoft et al. (Allentoft et al., 2015) and identified *Y. pestis* DNA sequences in 7 of the 101 individuals. Descriptions of the archaeological sites are given in Supplemental Experimental Procedures and Table S1.

Generation of Additional Sequence Data

In order to increase the depth of coverage on the *Y. pestis* genomes we sequenced more on these seven DNA extracts. Library construction was conducted as in (Allentoft et al., 2015). Briefly, double stranded and blunt-ended DNA libraries were prepared using the NEBNext DNA Sample Prep Master Mix Set 2 (E6070) and Illumina-specific adapters (Meyer and Kircher, 2010). The libraries were "shot-gun" sequenced in two pools on Illumina HiSeq2500 platforms using 100-bp single-read chemistry. We sequenced 32 lanes generating a total of 11.2 billion new DNA sequences for this study. Reads for the seven *Y. pestis* samples are available from ENA: PRJEB10885. Individual sample accessions numbers are available in Table S2.

Creation of Database for Identification of *Y. pestis* Reads

To identify *Y. pestis* reads in the Bronze Age dataset (Allentoft et al., 2015) we first created a database of all previously sequenced *Y. pestis* strains (n = 140), *Y. pseudotuberculosis* strains (n = 30), *Y. similis* strains (n = 5), and a selection of *Y. enterocolitica* strains (n = 4) (Supplemental Experimental Procedures and Table S2). The genomes were either downloaded from NCBI or downloaded as reads and de novo assembled using SPAdes-3.5.0 (Bankevich et al., 2012) with the–careful and–cov-cutoff auto options.

Identification and Assembly of *Y. pestis* From Ancient Samples

Raw reads were trimmed for adaptor sequences using AdapterRemoval-1.5.4 (Lindgreen, 2012). Additionally leading and trailing Ns were removed as well as bases with quality 2 or less. Hereafter, the trimmed reads with a length of at least 30 nt were mapped using bwa mem (local alignment) (Li and Durbin, 2009) to the database of *Y. pestis*, *Y. pseudotuberculosis*, *Y. similis*, and *Y. enterocolitica* mentioned above. Reads with a match to any of the sequences in this database were aligned separately to three different reference genomes: *Yersinia pestis* CO92 genome including the associated plasmids pCD1, pMT1, pPCP1 (Parkhill et al., 2001); *Yersinia pseudotuberculosis* IP32953 including the associated plasmids (Chain et al., 2004); *Yersinia pestis biovar Microtus* 91001 and associated plasmids (Zhou et al., 2004). This alignment was performed using bwa aln (Li and Durbin, 2009) with the seed option disabled for better sensitivity for ancient data, enforcing global alignment of the read to the reference genome. Each sequencing run was merged to library level and duplicates removed using Picard-1.124 (http://broadinstitute.github.io/picard/), followed by merging to per sample alignment files. These files were filtered for a mapping quality of 30 to only retain high quality alignments and the base qualities were re-scaled for DNA damage using MapDamage 2.0 (Jónsson et al., 2013). We defined *Y. pestis* as present in a sample if the mapped depth of the CO92 reference sequences were higher or equal to 0.1X and if the reads covered at least 10% of the chromosome and each of the plasmids. The assembly of Justinian, Black Death, and the modern samples were performed similarly and is described in detail in the Supplemental Experimental Procedures.

Coverage, Depth and Mappability Analyses

We calculated the coverage of the individual sample alignments versus the *Y. pestis* CO92 reference genome using Bedtools (Quinlan and Hall, 2010) and plotted this using Circos (Krzywinski et al., 2009). For the chromosome, the coverage was calculated in 1 kbp windows and for the plasmids in 100 bp windows. Mappability was calculated using GEM-mappability library using a k-mer size of 50, which is similar to the average length of the trimmed and mapped *Y. pestis* reads (average length 43–65 bp). Statistics of the coverage and depth are given in Tables S3 and S4.

DNA Decay Rates

We investigated the molecular degradation signals obtained from the sequencing data. Based on the negative exponential relationship between frequency and sequence length, we estimated for each sample the DNA damage fraction (λ, per bond), the average fragment length (1/ λ), the DNA decay rate (k, per bond per year), and the molecular half-lives of 100 bp fragments (Allentoft et al., 2012). We compared these DNA decay estimates for *Y. pestis* to the decay of endogenous human DNA from the host individuals. If the plague DNA is authentic and ancient, a correlation is expected between the rate of DNA decay in the human host and in *Y. pestis*, because the DNA has been exposed to similar environmental conditions for the same amount of time. See Supplemental Experimental Procedures for additional information.

Figure 3. Authenticity of *Y. pestis* DNA

(A) DNA damage patterns for RISE505 and RISE509. The frequencies of all possible mismatches observed between the *Y. pestis* CO92 chromosome and the reads are reported in gray as a function of distance from 5′ (left panel, first 25 nucleotides sequenced) and distance to 3′ (right panel, last 25 nucleotides). The typical DNA damage mutations C>T (5′) and G>A (3′) are reported in red and blue, respectively.

(B) Ancient DNA damage patterns (n = 7) of the reads aligned to the CO92 chromosome and the *Y. pestis* associated plasmids pMT1, pCD1 and pPCP1. The boxplots show the distribution of C-T damage in the 5′ of the reads. The lower and upper hinges of the boxes correspond to the 25th and 75th percentiles, the whiskers represent the 1.5 inter-quartile range (IQR) extending from the hinges, and the dots represent outliers from these.

(C) DNA fragment length distributions from RISE505 and RISE509 samples representing both the *Y. pestis* DNA and the DNA of the human host. The declining part of the distributions is fitted to an exponential model (red).

(D) Linear correlation (red) between the decay constant in the DNA of the human host and the associated *Y. pestis* DNA extracted from the same individual ($R^2 = 0.55$, $p = 0.055$). The decay constant (λ) describes the damage fraction (i.e., the fraction of broken bonds on the DNA strand).

(E) Distribution of edit distance of high quality reads from RISE505 and RISE509 samples mapped to either *Y. pestis* (dark gray) or *Y. pseudotuberculosis* (light gray) reference genomes. The reads have a higher affinity to *Y. pestis* than to *Y. pseudotuberculosis*.

(F) Plots of actual coverage versus expected coverage for the 101 screened samples. Expected coverage was computed taking into account read length distributions, mappable fractions of reference sequences, and the deletions in pMT1 for some of the samples. Samples assumed to contain *Y. pestis* are shown in blue and RISE392 that is classified as not *Y. pestis* appears is shown in red. See also Figure S1 and S2, Table S3.

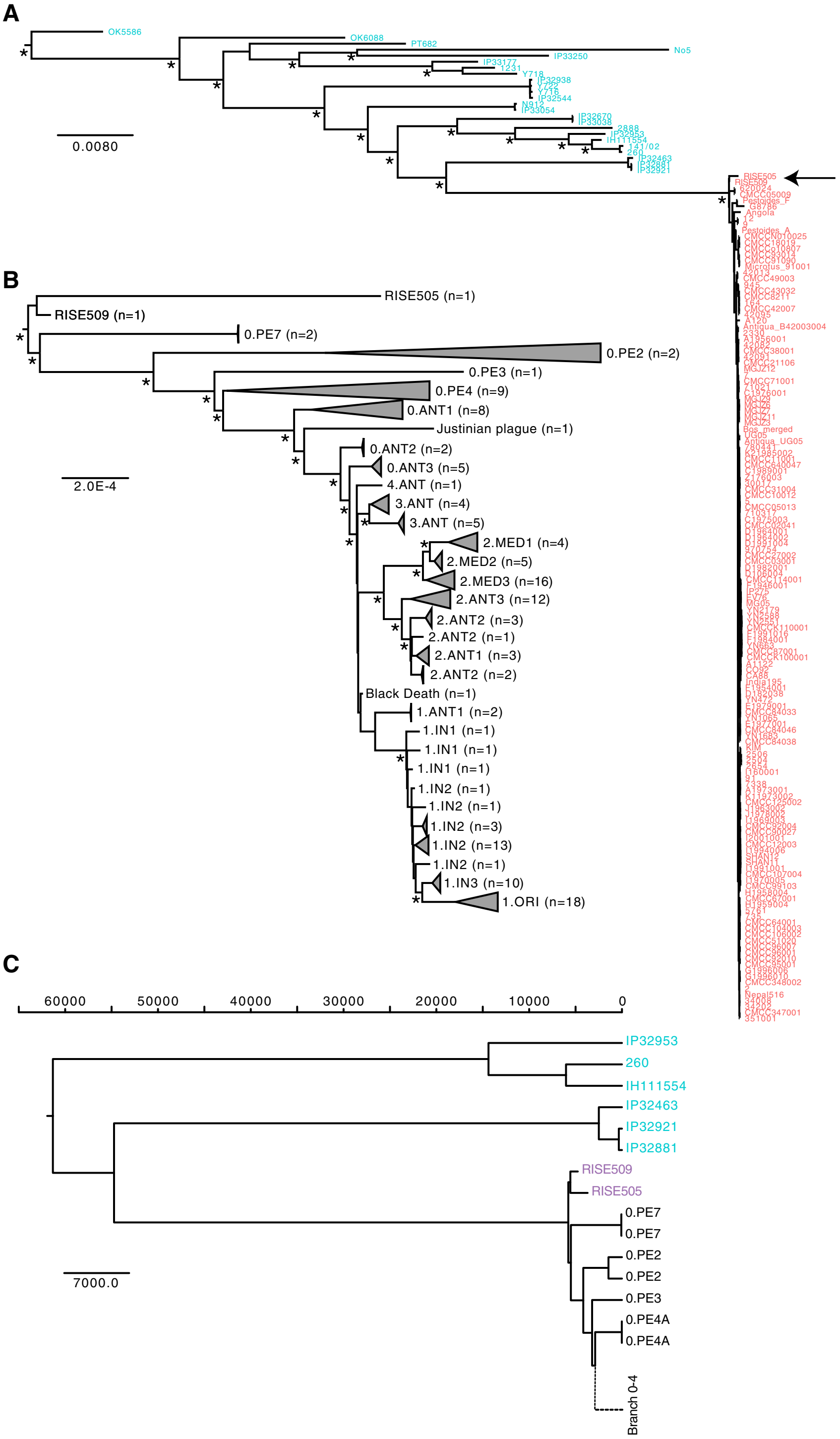

Figure 4. Phylogenetic Reconstructions

(A) Maximum Likelihood reconstruction of the phylogeny of *Y. pseudotuberculosis* (blue) and *Y. pestis* (red). The tree is rooted using *Y. similis* (not shown). The full tree including three additional *Y. pseudotuberculosis* strains (O:15 serovar) can be seen in Figure S4. Major branching nodes within *Y. pseudotuberculosis* with > 95% bootstrap support are indicated with an asterisk and branch lengths are given as substitutions per site.

(B) Maximum Likelihood reconstruction of the phylogeny in (A) showing only the *Y. pestis* clade. The clades are collapsed by population according to branches and serovars, as given in (Achtman et al., 1999, 2004; Cui et al., 2013). See Figure S4 for an uncollapsed tree and Table S2 for details on populations. Nodes with more than 95% bootstrap support are indicated with an asterisk and branch lengths are given as substitutions per site.

(C) BEAST2 maximum clade credibility tree showing median divergence dates. Branch lengths are given as years before the present (see Divergence estimations in Experimental Procedures). Only the *Y. pseudotuberculosis* (blue), the ancient *Y. pestis* samples (magenta) and the most basal branch 0 strains (black) are shown. For a full tree including all *Y. pestis* see Figure S5. See also Figure S3, S4, and S5 and Table S5.

Comparison of Samples to *Y. pestis* and *Y. pseudotuberculosis* Reference Genomes

We used the alignments of several sets of reads (*Y. pestis*, *Y. pseudotuberculosis*, and *Y. similis*) to *Y. pestis* CO92 and the *Y. pseudotuberculosis* IP32953 genomes. Per sample we determined the distribution of edit-distances (mismatches) of the reads versus the particular reference genome. We used these distributions to build a Naive Bayesian classifier to classify whether reads were originating from *Y. pestis*, *Y. pseudotuberculosis*, or *Y. similis*. See Supplemental Experimental Procedures and Table S3.

Expected versus Actual Coverage

We estimated the expected coverage of *Y. pestis* given a specific sequencing depth and correlated that with the actual coverage of a genome per sample. Expected coverage was calculated as

$$c = 1 - \prod_{i=1}^{N} \left(1 - \frac{l_i}{g}\right)^{r_i}$$

where the reads have N different lengths, l_1 to l_N with counts r_1 to r_N. To account for mappability we determined the mappable fraction for each reference sequence using kmers of length 40, 50, and 60, and then used the mappability value with the k-mer length closest to the actual average read length for each sample/reference combination. For more information see Supplemental Experimental Procedures.

Genotyping For Phylogenetic Analyses

Alignments of all strains versus *Y. pseudotuberculosis* IP32953 was used as reference for genotyping the consensus sequences for all samples used in the phylogeny. The samples were genotyped individually using samtools-0.1.18 and bcftools-0.1.17 (Li et al., 2009) and hereafter filtered (Supplemental Experimental Procedures). Based on *Y. pseudotuberculosis* IP32953 gene annotations, the consensus sequences for each gene and sample were extracted. Because of the divergence between *Y. pestis* and *Y. pseudotuberculosis*, a number of gene sequences displayed high rates of missing bases and we removed genes where 20 or more modern *Y. pestis* samples had >10% missingness. This corresponded to a total of 985 genes, leaving data from 3,141 genes that were merged into

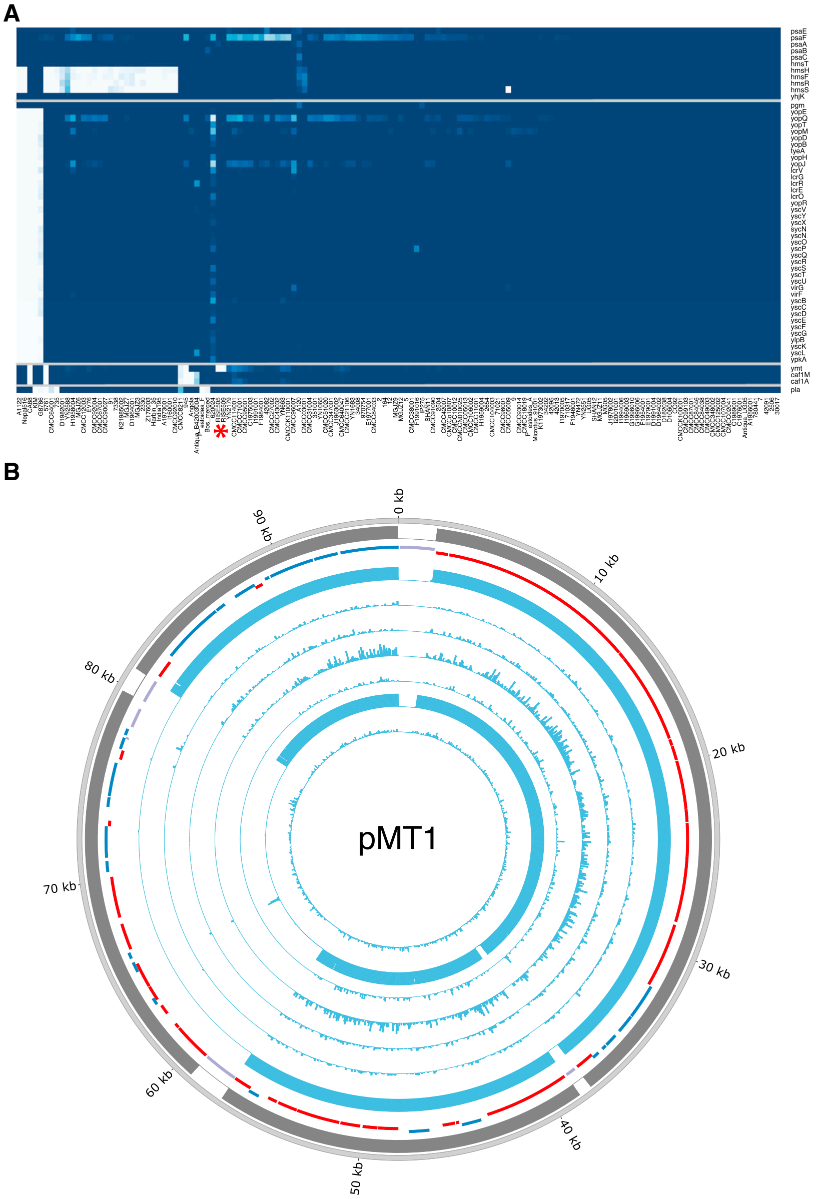

Figure 5. Identification of Virulence Genes

(A) Gene coverage heatmap of 55 virulence genes (rows) in 140 *Y. pestis* strains (columns). Sample ordering is based on hierarchical clustering (not shown) of the gene coverage distributions. RISE505 and RISE509 are marked with a red asterisk. Coloring goes from 0% gene coverage (white) to 100% gene coverage (blue).

(B) Depth of coverage of high quality reads mapping across pMT1. Outer ring is mappability (gray), genes (RNA: black, transposon: purple, positive strand: blue, negative strand: red) and then the RISE samples ordered after direct AMS dating. Sample ordering are RISE509, RISE511, RISE00, RISE386, RISE139, RISE505 and RISE397. See also Figure S6, Tables S2, S6, and S7. AMS: Accelerator Mass Spectrometry.

a supermatrix. We created two different supermatrices, one with *Y. similis*, *Y. pseudotuberculosis*, and *Y. pestis* containing 173 taxa × 3,141 genes that was used for the initial phylogeny (Figure 4A). The second supermatrix consisted of all *Y. pestis* strains and the genomes from the two closest *Y. pseudotuberculosis* clades, which was used for the divergence time estimations.

Phylogenetics

The alignments were partitioned by codon position and analyzed with jmodeltest-2.1.7 (Darriba et al., 2012) to test for the best fitting substitution model. All decision criteria (Akaike, Bayesian, and Decision theory) found the Generalized Time Reversible substitution model with gamma distributed rates, using four rate categories, and a proportion of invariable sites (GTR+G+I) to be the best fit for each of the three codon partitions. To test for recombination across the chromosome we estimated linkage disequilibrium (LD) using 141 *Y. pestis* strains. A total of 482 bi-allelic single nucleotide variations (SNVs), with a minor allele frequency of 5% or higher were extracted. For all pairs of the extracted SNVs, the LD r^2 was calculated using PLINK 1.9 (Chang et al., 2015) and plotted against the physical distance between the pairs. We reconstructed the phylogeny from the codon-partitioned supermatrix using RAxML-8.1.15 (Stamatakis, 2014) with the GTR+G+I substitution model. Bootstraps were performed by generating 100 bootstrap replicates and their corresponding parsimony starting trees using RAxML. Hereafter, a standard Maximum Likelihood inference was run on each bootstrap replicate, and the resulting best trees were merged and drawn on the best ML tree. Initial phylogenies placed the *Y. pestis* Harbin strain with an unusual long branch inside the 1.ORI clade and it was excluded from further analysis. Additionally *Y. pseudotuberculosis* SP93422 (serotype O:15), *Y. pseudotuberculosis* WP-931201 (serotype O:15) and *Y. pseudotuberculosis* Y248 (serotype unknown) was in a clade with long branch lengths and were therefore also omitted (see Figure S4).

Heterozygosity Estimates

We determined heterozygosity by down-sampling the *Y. pestis* bam-files to the same average depth as the corresponding RISE samples, genotyped each of the samples and extracted heterozygote calls with a depth equal to or higher than 10. All transitions were excluded. See Supplemental Experimental Procedures for detailed information.

Divergence Estimations

To date the divergence time for *Y. pestis* and nodes within the *Y. pestis* clade we performed Bayesian Markov Chain Monte Carlo simulations using BEAST-2.3.0 (Bouckaert et al., 2014) and the BEAGLE library v2.1.2 (Ayres et al., 2012). We used the codon-partitioned supermatrix that included the two closest *Y. pseudotuberculosis* clades, with unlinked substitution models, GTR+G+I with eight gamma rate categories and unlinked clock models. Dates were set as years ago with the RISE509, RISE505, Justinian and Black Death samples set to 4,761, 3,701, 1,474, and 667 years ago, respectively. All unknown dates were set to 0 years ago. We followed previous work (Cui et al., 2013; Wagner et al., 2014) and applied a lognormal relaxed clock, assuming a constant population size. We re-rooted the ML tree from RAxML so that the root was placed between the two *Y. pseudotuberculosis* clades (IP32953, 260, IH111554) and (IP32921, IP32881, IP32463) and used this as the starting tree. Based on the ML tree we defined the closets *Y. pseudotuberculosis* clade (IP32921, IP32881, IP32463) and the *Y. pestis* clade as a monophyletic group and defined a uniform prior with 1,000 and 100,000 years as minimum and maximum bounds. We ran 20 independent parallel BEAST chains sampling every 2,000 states for between 52 and 64 million states using a total of 240,000 core hours. The chains were combined using LogCombiner discarding the initial 10 million states as burn-in. The combined post burn-in data represented 961 million states and

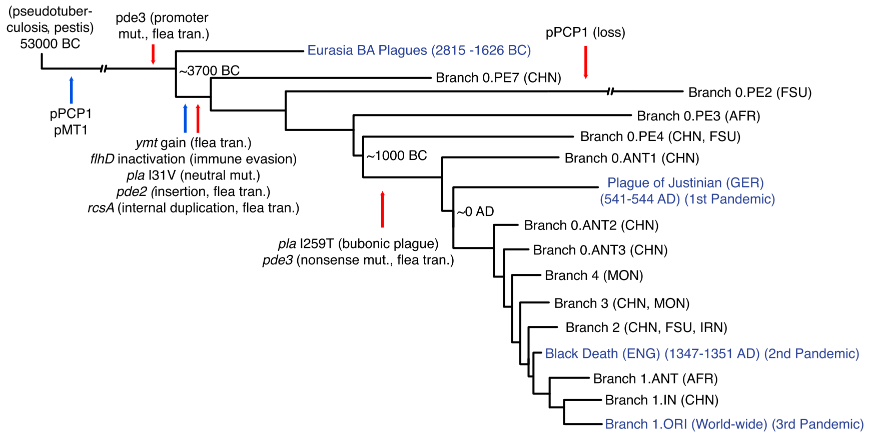

Figure 6. Schematic of *Y. pestis* Evolution

Representation of *Y. pestis* phylogeny and important evolutionary events since divergence from *Y. pseudotuberculosis*. Genetic gains (blue) and genetic loss or loss of function mutations (red) are indicated by arrows. Historical recorded pandemics are indicated in blue text. The calendric years indicates the primary outbreak of the Pandemic. Node dates are median divergence times from the BEAST analysis. The events are based on information from this study and Sun et al., 2014. We used the VCFs generated from all *Y. pestis* samples (n = 142) (Table S2) to verify on which branches the genetic events occurred. The figure is based on current knowledge and is subject to change with addition of new samples. See also Figure S5 and Table S5. BA: Bronze Age, CHN: China, FSU: Former Soviet Union, AFR: Africa, GER: Germany, MON: Mongolia, IRN: Iran, ENG: England, flea tran: flea transmission, mut.: mutation.

the effective sample sizes (ESS) for the posterior was 398, for the TreeHeight 238 and for the MRCA for *Y. pseudotuberculosis* and *Y. pestis* 216. All other parameters had ESS > 125. We then sampled 1/5 of the trees from each chain and combined them for a total of 192,406 trees that were summarized using TreeAnnotator producing a maximum clade credibility tree of median heights. We additionally ran BEAST2 sampling the priors only (and disregarding sequence information) and found the posterior distribution no different than the priors used. It suggests that the posterior distributions recovered when considering full sequence alignments are driven by the sequence information and are not mere by-products of the sampling structure in our dataset (Figure S5).

Analysis of Virulence Associated Genes

To assess the potential virulence of the ancient *Y. pestis* strains, we identified 55 genes previously reported to be associated with virulence of *Y. pestis* (Supplemental Experimental Procedures and Table S6 for details). Based on the alignments to *Y. pestis* CO92 reference genome we determined the fraction of the each gene sequence that was covered by at least one read for each *Y. pestis* sample. Additionally, because the different region 4 (DFR4) (Radnedge et al., 2002) has been associated with virulence, but is not present in the CO92 genome, we used the alignments to *Y. pestis microtus* 91001 to determine the presence of this region (Supplemental Experimental Procedures). We note that the absence of KIM pPCP1 is due to it being missing from the reference genome, but that it has been reported to be present in KIM strains (Hu et al., 1998). The genotypes were generated as described above and the variant call format (VCF) files from these analyses are available at http://www.cbs.dtu.dk/suppl/plague/. For detailed information on genotyping of *pde2*, *pde3*, *rscA*, *pla*, and *flhD* see Supplemental Experimental Procedures.

Identification of the Missing *ymt* Region on pMT1

Most of the regions that were unmapped could be associated with low mappability. However, we identified a region from 59–78 kb on pMT1 that could not be explained by low mappability. From the depth of coverage this region was absent in all of our ancient plague genomes, except for RISE397 (Figure 5). We tested for the significance of this by comparing the distribution of gene depths within and outside of the missing region using the Wilcoxon rank-sum test (Table S7). For all samples except RISE397 the region had a median depth of 0X and the gene depth distributions were significantly different compared to the remaining pMT1 plasmid genes (p values < 1E-9). For the RISE397 sample, the regions had 0.43X and 0.42X median depths and there was no significant difference in the depth of the genes in the two regions (p value 0.77).

ACCESSION NUMBERS

The accession number for the reads for the seven *Y. pestis* samples reported in this paper is ENA: PRJEB10885.

SUPPLEMENTAL INFORMATION

Supplemental Information includes Supplemental Experimental Procedures, six figures, and eight tables and can be found with this article online at http://dx.doi.org/10.1016/j.cell.2015.10.009.

AUTHOR CONTRIBUTIONS

Conceptualization, K-G.S., R.N., K.K. and E.W.; methodology, S.R., M.E.A., A.G.P. and H.B.N.; software, S.R., K.N., M. Sikora, M. Schubert, and A.V.D.; Formal Analysis, S.R., M.E.A., K.N., M. Sikora, A.G.P., A.V.D. and M. Schubert.; Investigation, M.E.A. and K-G.S.; Resources, S.B., P.A., M.V.K., A.E., A. Gnuni, A.K., I.L., M.M., V.M., A. Gromov, D.P., L.S., L.V., L.Y. and T.S-P.; Writing – Original Draft, S.R., M.E.A., K.N., L.O., K-G.S., A.G.P., R.A.F., M.M.L., R.N., K.K. and E.W.; Writing Review & Editing, S.R., M.E.A., K.N., L.O., M. Sikora, K-G.S., A.G.P., A.V.D., C.M.O., R.A.F., M.M.L., R.N., K.K. and E.W.; Visualization, S.R. M.E.A., K-G.S. and A.G.P.; Supervision, L.O., T.S-P., R.N., K.K. and E.W.; Funding Acquisition, K.K. and E.W.

ACKNOWLEDGMENTS

The project was funded by The European Research Council (FP/2007-2013, grant 269442, The Rise), Marie Curie Actions of the European Union (FP7/2007-2013, grant 300554), The Villum Foundation (Young Investigator Programme, grant 10120), University of Copenhagen (KU2016 Programme), The Danish National Research Foundation, and The Lundbeck Foundation. A.V.D. was supported by the National Science Foundation Postdoctoral Research Fellowship in Biology under grant 1306489. S.B. was supported financially by the Novo Nordisk Foundation Grant agreement NNF14CC0001. We thank Jesper Stenderup for technical assistance and want to acknowledge the Danish national supercomputer – Computerome (computerome.cbs.dtu.dk) for the computational resources to perform the BEAST divergence estimations.

Received: August 6, 2015
Revised: September 30, 2015
Accepted: October 2, 2015
Published: October 22, 2015

REFERENCES

Achtman, M., Zurth, K., Morelli, G., Torrea, G., Guiyoule, A., and Carniel, E. (1999). Yersinia pestis, the cause of plague, is a recently emerged clone of Yersinia pseudotuberculosis. Proc. Natl. Acad. Sci. USA *96*, 14043–14048.

Achtman, M., Morelli, G., Zhu, P., Wirth, T., Diehl, I., Kusecek, B., Vogler, A.J., Wagner, D.M., Allender, C.J., Easterday, W.R., et al. (2004). Microevolution

and history of the plague bacillus, Yersinia pestis. Proc. Natl. Acad. Sci. USA *101*, 17837–17842.

Allentoft, M.E., Collins, M., Harker, D., Haile, J., Oskam, C.L., Hale, M.L., Campos, P.F., Samaniego, J.A., Gilbert, M.T.P., Willerslev, E., et al. (2012). The half-life of DNA in bone: measuring decay kinetics in 158 dated fossils. Proc. Biol. Sci. *279*, 4724–4733.

Allentoft, M.E., Sikora, M., Sjögren, K.-G., Rasmussen, S., Rasmussen, M., Stenderup, J., Damgaard, P.B., Schroeder, H., Ahlström, T., Vinner, L., et al. (2015). Population genomics of Bronze Age Eurasia. Nature *522*, 167–172.

Anthony, D. (2007). The Horse, The Wheel and Language. How Bronze-Age riders from the Eurasian Steppes Shaped the Modern World (Princeton: Princeton University Press).

Ayres, D.L., Darling, A., Zwickl, D.J., Beerli, P., Holder, M.T., Lewis, P.O., Huelsenbeck, J.P., Ronquist, F., Swofford, D.L., Cummings, M.P., et al. (2012). BEAGLE: an application programming interface and high-performance computing library for statistical phylogenetics. Syst. Biol. *61*, 170–173.

Bankevich, A., Nurk, S., Antipov, D., Gurevich, A.A., Dvorkin, M., Kulikov, A.S., Lesin, V.M., Nikolenko, S.I., Pham, S., Prjibelski, A.D., et al. (2012). SPAdes: a new genome assembly algorithm and its applications to single-cell sequencing. J. Comput. Biol. *19*, 455–477.

Bercovier, H., Mollaret, H.H., Alonso, J.M., Brault, J., Fanning, G.R., Steigerwalt, A.G., and Brenner, D.J. (1980). Intra- and interspecies relatedness of Yersinia pestis by DNA hybridization and its relationship to Yersinia pseudotuberculosis. Curr. Microbiol. *4*, 225–229.

Bos, K.I., Schuenemann, V.J., Golding, G.B., Burbano, H.A., Waglechner, N., Coombes, B.K., McPhee, J.B., DeWitte, S.N., Meyer, M., Schmedes, S., et al. (2011). A draft genome of Yersinia pestis from victims of the Black Death. Nature *478*, 506–510.

Bouckaert, R., Heled, J., Kühnert, D., Vaughan, T., Wu, C.-H., Xie, D., Suchard, M.A., Rambaut, A., and Drummond, A.J. (2014). BEAST 2: a software platform for Bayesian evolutionary analysis. PLoS Comput. Biol. *10*, e1003537.

Carniel, E. (2003). Evolution of pathogenic Yersinia, some lights in the dark. In The Genus Yersinia: Entering the Functional Genomic Era. In The Genus Yersinia, M. Skurnik, J.A. Bengoechea, and K. Granfors, eds. (Boston: Springer US), pp. 3–11.

Chain, P.S.G., Carniel, E., Larimer, F.W., Lamerdin, J., Stoutland, P.O., Regala, W.M., Georgescu, A.M., Vergez, L.M., Land, M.L., Motin, V.L., et al. (2004). Insights into the evolution of Yersinia pestis through whole-genome comparison with Yersinia pseudotuberculosis. Proc. Natl. Acad. Sci. USA *101*, 13826–13831.

Chang, C.C., Chow, C.C., Tellier, L.C., Vattikuti, S., Purcell, S.M., and Lee, J.J. (2015). Second-generation PLINK: rising to the challenge of larger and richer datasets. Gigascience *4*, 7.

Cui, Y., Yu, C., Yan, Y., Li, D., Li, Y., Jombart, T., Weinert, L.A., Wang, Z., Guo, Z., Xu, L., et al. (2013). Historical variations in mutation rate in an epidemic pathogen, Yersinia pestis. Proc. Natl. Acad. Sci. USA *110*, 577–582.

Darriba, D., Taboada, G.L., Doallo, R., and Posada, D. (2012). jModelTest 2: more models, new heuristics and parallel computing. Nat. Methods *9*, 772.

Drancourt, M., and Raoult, D. (2002). Molecular insights into the history of plague. Microbes Infect. *4*, 105–109.

Drancourt, M., Aboudharam, G., Signoli, M., Dutour, O., and Raoult, D. (1998). Detection of 400-year-old Yersinia pestis DNA in human dental pulp: an approach to the diagnosis of ancient septicemia. Proc. Natl. Acad. Sci. USA *95*, 12637–12640.

Haak, W., Lazaridis, I., Patterson, N., Rohland, N., Mallick, S., Llamas, B., Brandt, G., Nordenfelt, S., Harney, E., Stewardson, K., et al. (2015). Massive migration from the steppe was a source for Indo-European languages in Europe. Nature *522*, 207–211.

Harbeck, M., Seifert, L., Hänsch, S., Wagner, D.M., Birdsell, D., Parise, K.L., Wiechmann, I., Grupe, G., Thomas, A., Keim, P., et al. (2013). Yersinia pestis DNA from skeletal remains from the 6(th) century AD reveals insights into Justinianic Plague. PLoS Pathog. *9*, e1003349.

Hayashi, F., Smith, K.D., Ozinsky, A., Hawn, T.R., Yi, E.C., Goodlett, D.R., Eng, J.K., Akira, S., Underhill, D.M., and Aderem, A. (2001). The innate immune response to bacterial flagellin is mediated by Toll-like receptor 5. Nature *410*, 1099–1103.

Hinnebusch, B.J. (2005). The evolution of flea-borne transmission in Yersinia pestis. Curr. Issues Mol. Biol. *7*, 197–212.

Hinnebusch, B.J., Rudolph, A.E., Cherepanov, P., Dixon, J.E., Schwan, T.G., and Forsberg, A. (2002). Role of Yersinia murine toxin in survival of Yersinia pestis in the midgut of the flea vector. Science *296*, 733–735.

Hinz, M., Feeser, I., Sjögren, K.-G., and Müller, J. (2012). Demography and the intensity of cultural activities: an evaluation of Funnel Beaker Societies (4200–2800 cal BC). J. Archaeol. Sci. *39*, 3331–3340.

Hu, P., Elliott, J., McCready, P., Skowronski, E., Garnes, J., Kobayashi, A., Brubaker, R.R., and Garcia, E. (1998). Structural organization of virulence-associated plasmids of Yersinia pestis. J. Bacteriol. *180*, 5192–5202.

Jónsson, H., Ginolhac, A., Schubert, M., Johnson, P.L.F., and Orlando, L. (2013). mapDamage2.0: fast approximate Bayesian estimates of ancient DNA damage parameters. Bioinformatics *29*, 1682–1684.

Kristiansen, K., and Larsson, T.B. (2005). The Rise of Bronze Age Society. Travels, Transmissions and Transformations (New York: Cambridge University Press).

Krzywinski, M., Schein, J., Birol, I., Connors, J., Gascoyne, R., Horsman, D., Jones, S.J., and Marra, M.A. (2009). Circos: an information aesthetic for comparative genomics. Genome Res. *19*, 1639–1645.

Li, H., and Durbin, R. (2009). Fast and accurate short read alignment with Burrows-Wheeler transform. Bioinformatics *25*, 1754–1760.

Li, H., Handsaker, B., Wysoker, A., Fennell, T., Ruan, J., Homer, N., Marth, G., Abecasis, G., and Durbin, R.; 1000 Genome Project Data Processing Subgroup (2009). The Sequence Alignment/Map format and SAMtools. Bioinformatics *25*, 2078–2079.

Lindgreen, S. (2012). AdapterRemoval: easy cleaning of next-generation sequencing reads. BMC Res. Notes *5*, 337.

Lindler, L.E., Plano, G.V., Burland, V., Mayhew, G.F., and Blattner, F.R. (1998). Complete DNA sequence and detailed analysis of the Yersinia pestis KIM5 plasmid encoding murine toxin and capsular antigen. Infect. Immun. *66*, 5731–5742.

Little, L.K., Hays, J.N., Morony, M., Kennedy, H.N., Stathakopoulos, D., Sarris, P., Stoclet, A.J., Kulikowski, M., Maddicott, J., Dooley, A., et al. (2007). Plague and the end of antiquity: The pandemic of 541–750 (Cambridge University Press).

McNeill, W.H. (1976). Plagues and Peoples (New York: Anchor Books).

Meyer, M., and Kircher, M. (2010). Illumina sequencing library preparation for highly multiplexed target capture and sequencing. Cold Spring Harb. Protoc. *2010*, pdb.prot5448.

Minnich, S.A., and Rohde, H.N. (2007). A rationale for repression and/or loss of motility by pathogenic Yersinia in the mammalian host. Adv. Exp. Med. Biol. *603*, 298–310.

Morelli, G., Song, Y., Mazzoni, C.J., Eppinger, M., Roumagnac, P., Wagner, D.M., Feldkamp, M., Kusecek, B., Vogler, A.J., Li, Y., et al. (2010). Yersinia pestis genome sequencing identifies patterns of global phylogenetic diversity. Nat. Genet. *42*, 1140–1143.

Parkhill, J., Wren, B.W., Thomson, N.R., Titball, R.W., Holden, M.T., Prentice, M.B., Sebaihia, M., James, K.D., Churcher, C., Mungall, K.L., et al. (2001). Genome sequence of Yersinia pestis, the causative agent of plague. Nature *413*, 523–527.

Perry, R.D., and Fetherston, J.D. (1997). Yersinia pestis–etiologic agent of plague. Clin. Microbiol. Rev. *10*, 35–66.

Quinlan, A.R., and Hall, I.M. (2010). BEDTools: a flexible suite of utilities for comparing genomic features. Bioinformatics *26*, 841–842.

Radnedge, L., Agron, P.G., Worsham, P.L., and Andersen, G.L. (2002). Genome plasticity in Yersinia pestis. Microbiology *148*, 1687–1698.

Sebbane, F., Jarrett, C.O., Gardner, D., Long, D., and Hinnebusch, B.J. (2006). Role of the Yersinia pestis plasminogen activator in the incidence of distinct

septicemic and bubonic forms of flea-borne plague. Proc. Natl. Acad. Sci. USA *103*, 5526–5530.

Shennan, S., Downey, S.S., Timpson, A., Edinborough, K., Colledge, S., Kerig, T., Manning, K., and Thomas, M.G. (2013). Regional population collapse followed initial agriculture booms in mid-Holocene Europe. Nat. Commun. *4*, 2486.

Sodeinde, O.A., Subrahmanyam, Y.V., Stark, K., Quan, T., Bao, Y., and Goguen, J.D. (1992). A surface protease and the invasive character of plague. Science *258*, 1004–1007.

Stamatakis, A. (2014). RAxML version 8: a tool for phylogenetic analysis and post-analysis of large phylogenies. Bioinformatics *30*, 1312–1313.

Sun, Y.-C., Jarrett, C.O., Bosio, C.F., and Hinnebusch, B.J. (2014). Retracing the evolutionary path that led to flea-borne transmission of Yersinia pestis. Cell Host Microbe *15*, 578–586.

Treille, G., and Yersin, A. (1894). La peste bubonique à Hong Kong. VIIIe Congrès Int. D’hygiène Démographie.

Wagner, D.M., Klunk, J., Harbeck, M., Devault, A., Waglechner, N., Sahl, J.W., Enk, J., Birdsell, D.N., Kuch, M., Lumibao, C., et al. (2014). Yersinia pestis and the plague of Justinian 541–543 AD: a genomic analysis. Lancet Infect. Dis. *14*, 319–326.

Willerslev, E., and Cooper, A. (2005). Ancient DNA. Proc. Biol. Sci. *272*, 3–16.

Zhou, D., Tong, Z., Song, Y., Han, Y., Pei, D., Pang, X., Zhai, J., Li, M., Cui, B., Qi, Z., et al. (2004). Genetics of metabolic variations between Yersinia pestis biovars and the proposal of a new biovar, microtus. J. Bacteriol. *186*, 5147–5152.

Zimbler, D.L., Schroeder, J.A., Eddy, J.L., and Lathem, W.W. (2015). Early emergence of Yersinia pestis as a severe respiratory pathogen. Nat. Commun. *6*, 7487.

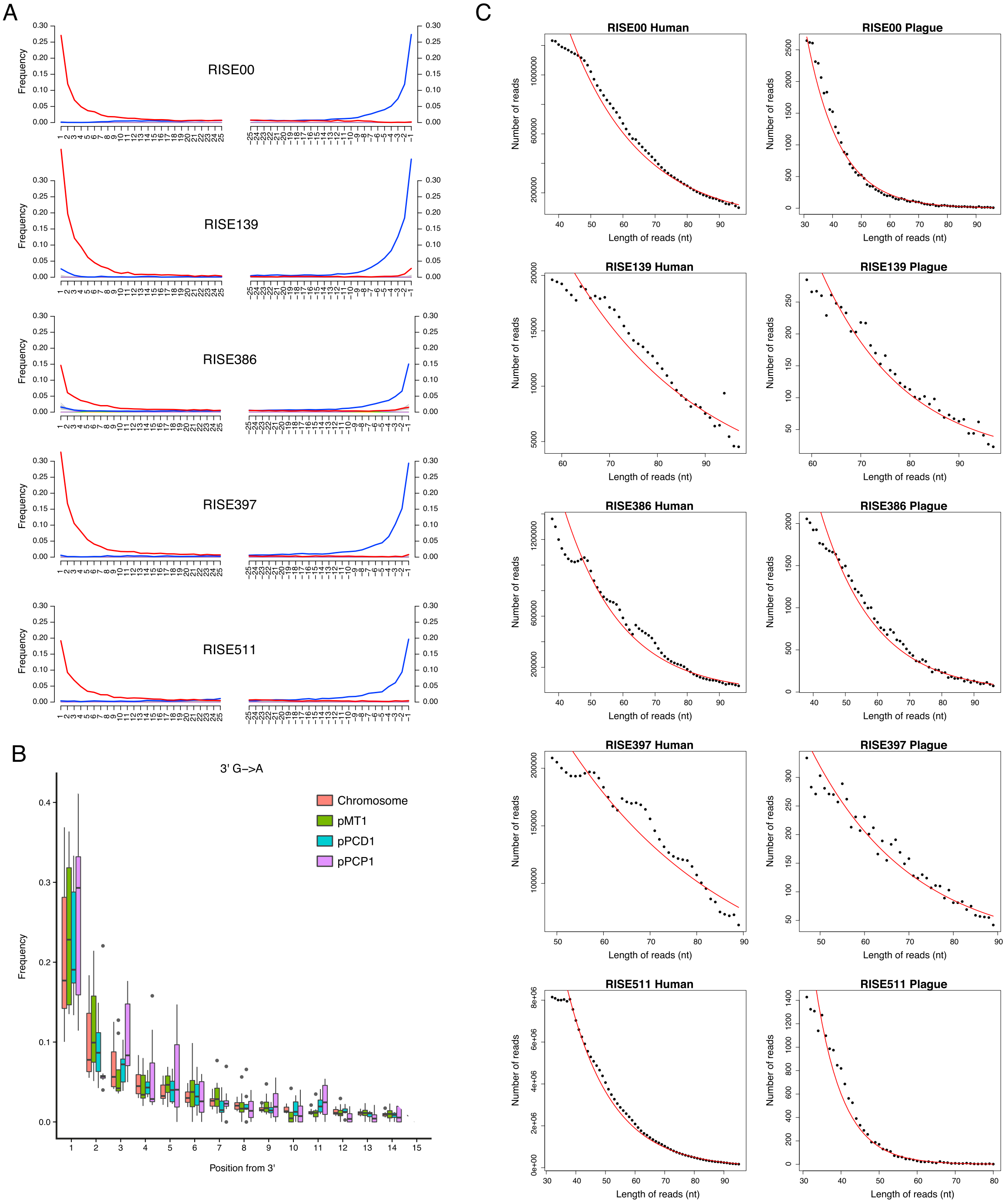

Figure S1. DNA Damage and Decay, Related to Figure 3

(A) DNA damage patterns for the five *Y. pestis* associated samples not shown in Figure 3. The frequencies of all possible mismatches observed between the *Y. pestis* CO92 chromosome and the reads are reported in gray as a function of distance from 5′ (left panel, first 25 nucleotides sequenced) and distance to 3′ (right panel, last 25 nucleotides). The typical DNA damage bases are C>T (5′) and G>A (3′) mutations are reported in red and blue, respectively.

(B) Ancient DNA damage patterns of the reads aligned to the CO92 chromosome and the *Y. pestis* associated plasmids pMT1, pCD1 and pPCP1. The boxplots show the distribution of G-A damage in the 3′ of the reads. The distributions are made from the seven *Y. pestis* samples. The lower and upper hinges of the boxes correspond to the 25th and 75th percentiles, the whiskers represent the 1.5 inter-quartile range (IQR) extending from the hinges, and the dots represent outliers from these.

(C) DNA fragment length distributions from five *Y. pestis* samples not shown in Figure 3 representing both the *Y. pestis* DNA and the DNA of the human host. The declining part of the distributions is fitted to an exponential model (red).

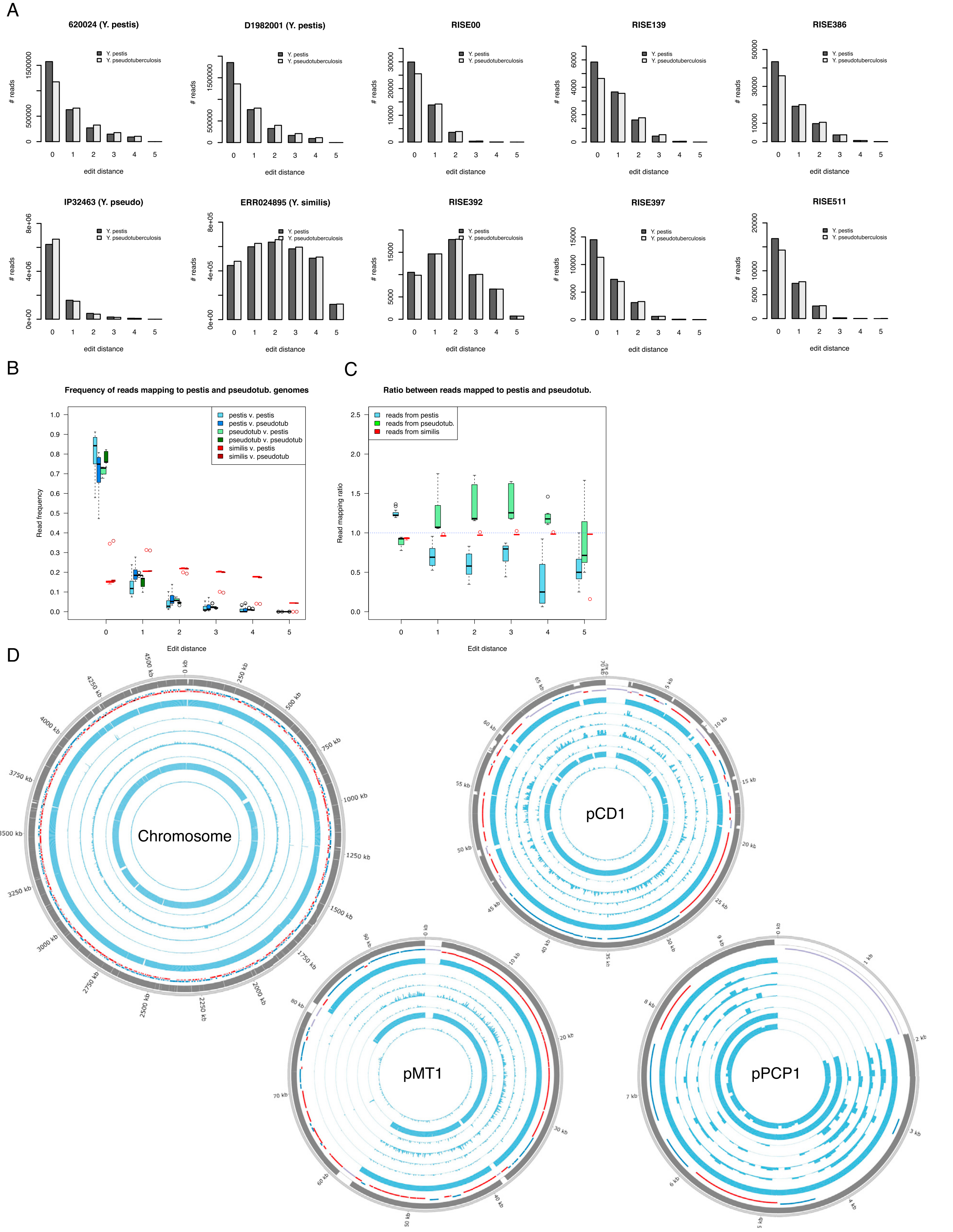

(legend on next page)

Figure S2. Mapping Affinity, Related to Figure 3

(A) Distribution of edit distance of high quality reads of known origin and the eight *Yersinia* associated samples. The investigated, known reads are from *Y. pestis* 620024 (0.PE7), *Y. pestis* D1982001 (1.IN2), *Y. pseudo* (IP32464) (from the clade closest to *Y. pestis*), and *Y. similis* (which is an outgroup to both *Y. pestis* and *Y. pseudotuberculosis*). For RISE00, RISE139, RISE386, RISE397, RISE505, RISE509 and RISE511 the reads are closer to *Y. pestis* than to *Y. pseudotuberculosis*, and there are far more hits at low edit distances (RISE505 and RISE509 are shown in Figure 3). This is consistent with these reads originating from *Y. pestis*. Reads from the RISE392 sample instead have more hits at higher edit distances and have similar distances to both the *Y. pestis* and *Y. pseudotuberculosis* reference genomes. This suggests that RISE392 is neither *Y. pestis* nor *Y. pseudotuberculosis*, but a more distantly related species.

(B) Distribution of the amount of reads mapping to the *Y. pestis* reference genome, at different edit distances. For each of the three investigated species (*Y. pestis* n = 10, *Y. pseudotuberculosis* n = 10, and *Y. similis* n = 5) several different sets of reads were mapped against the reference, and the number of reads matching at different edit distances was counted. For each edit distance the distribution of reads for each species is shown in the form of a boxplot. The lower and upper hinges of the boxes correspond to the 25th and 75th percentiles, the whiskers represent the 1.5 inter-quartile range (IQR) extending from the hinges, and the dots represent outliers from these.

(C) Ratio between the number of reads mapping to *Y. pestis* and the number of reads mapping to *Y. pseudotuberculosis*, for different edit distances, for three investigated species. Input data as in B. For each sample the ratio between the number of reads matching *Y. pestis*, and the number of reads matching *Y. pseudotuberculosis* was calculated, and the distribution of these ratios then shown in the form of a boxplot for each edit distance. These features were used to predict the taxonomy of unknown samples. The lower and upper hinges of the boxes correspond to the 25th and 75th percentiles, the whiskers represent the 1.5 inter-quartile range (IQR) extending from the hinges, and the dots represent outliers from these.

(D) Depth of coverage plots for the seven ancient *Y. pestis* samples mapped to the CO92 chromosome, pCD1, pMT1 and pPCP1. The RISE samples are ordered according to age where the oldest sample is the outermost histogram. Outer ring: Mappability (gray), genes (RNA: black, transposon: purple, positive strand: blue, negative strand: red), RISE509, RISE511, RISE00, RISE386, RISE139, RISE505 and RISE397 (blue). Depth histograms show sequence depth in 1 kb windows for the chromosome and 100 bp for the plasmids with a max of 5X depth for each ring. The plots were generated using Circos.

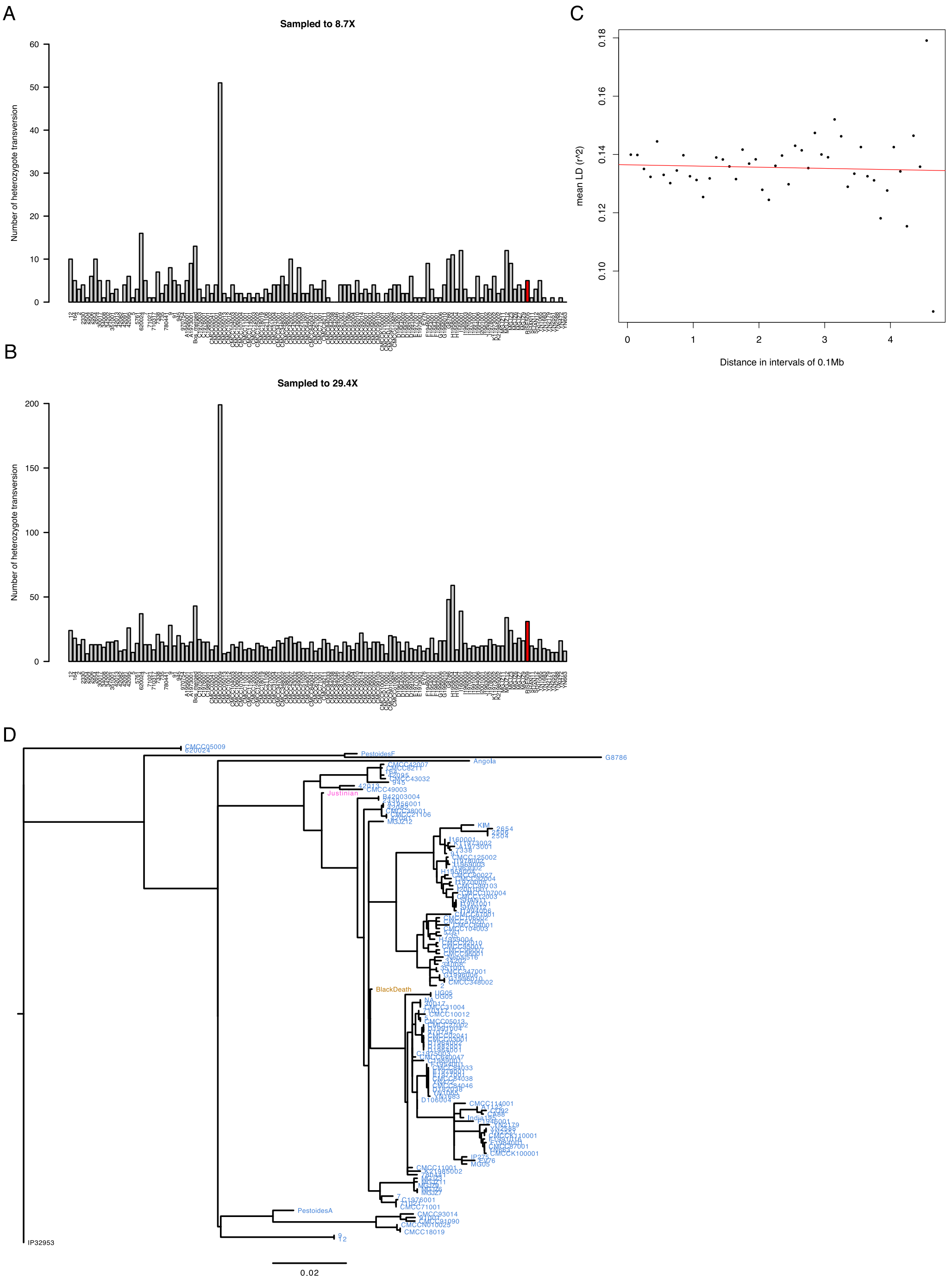

Figure S3. Phylogenetics, Related to Figure 4

(A and B) Heterozygosity estimates of RISE505 (A) and RISE509 (B), the respective ancient *Y. pestis* samples are shown in red. All samples were downsampled to the same depth as either RISE505 or RISE509 and the number of heterozygote transversions determined (y axis).

(C) Linkage Disequilibrium (LD) determined from 141 *Y. pestis* strains in 0.1Mb intervals across the *Y. pestis* CO92 chromosome. There is no decay in LD across the genome which means that there are no recombination and the phylogenetic tree can be averaged across the individual genes.

(D) Maximum Likelihood tree generated using RAxML and the 2,298 phylogenetic informative sites described by Morelli et al. (2010) and Cui et al. (2013). The strains are colored by species with *Y. pseudotuberculosis* IP32953 being black and *Y. pestis* blue. The Justinian plague sample and the Black Death samples are colored in magenta and brown, respectively. Branch lengths are substitutions per site.

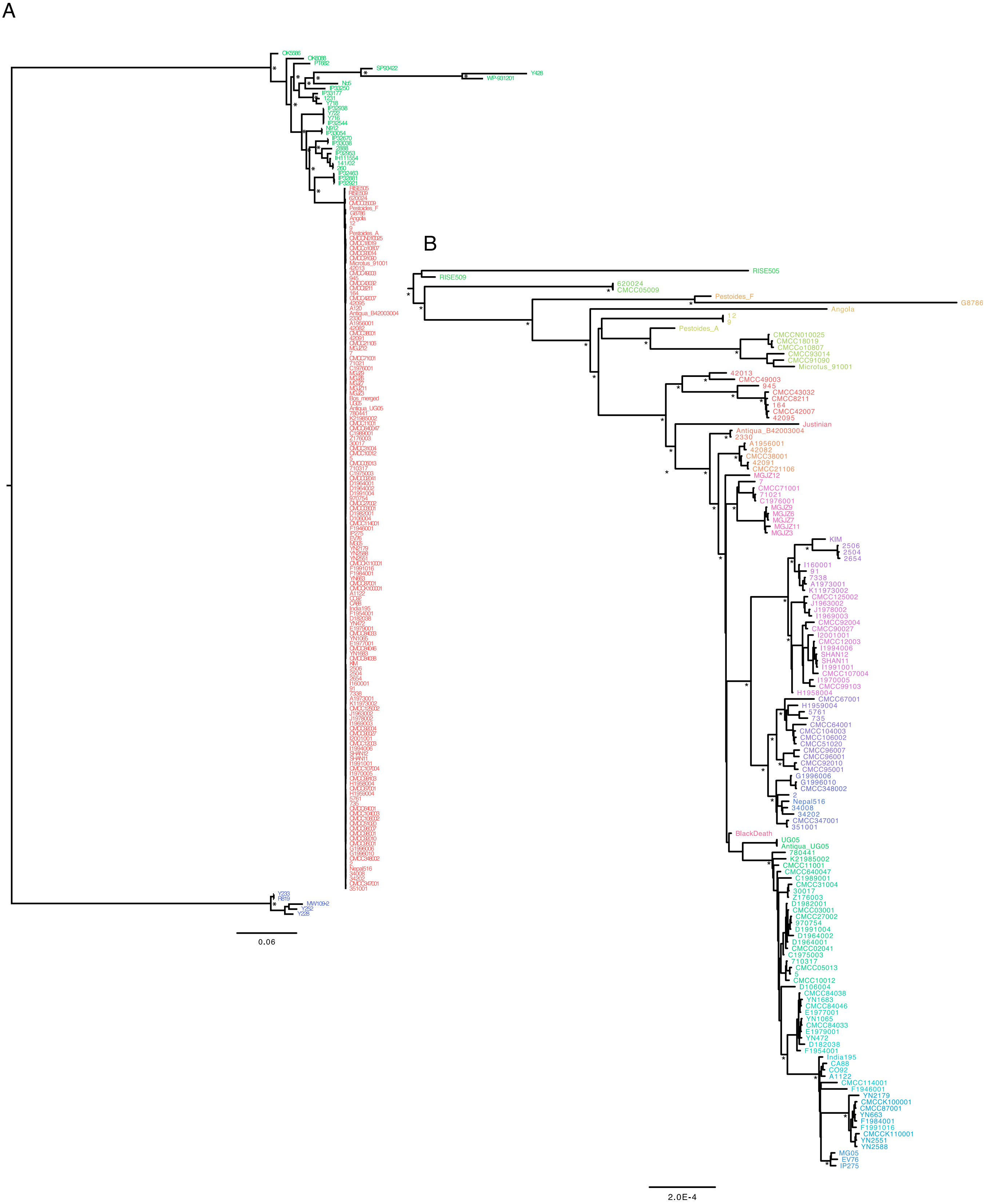

(legend on next page)

Figure S4. Phylogenetic Trees, Related to Figure 4

(A) Maximum Likelihood phylogeny of all strains used in the analysis. *Y. similis* (blue), *Y. pseudotuberculosis* (green) and *Y. pestis* (red). The strains that were excluded from the phylogeny in Figure 4A: SP93422, Y428 and WP-931201. Major branch nodes with bootstrap support > 95% are indicated with an asterisk. Branch lengths are substitutions per site.

(B) Maximum Likelihood tree of the *Y. pestis* clade only. The tree is the un-collapsed version of the tree shown in Figure 4B. Nodes marked with an asterisk have > 95% bootstrap support, not all internal nodes are marked with bootstrap values. Strain names are colored according to the population nomenclature in Table S2. Branch lengths are substitutions per site.

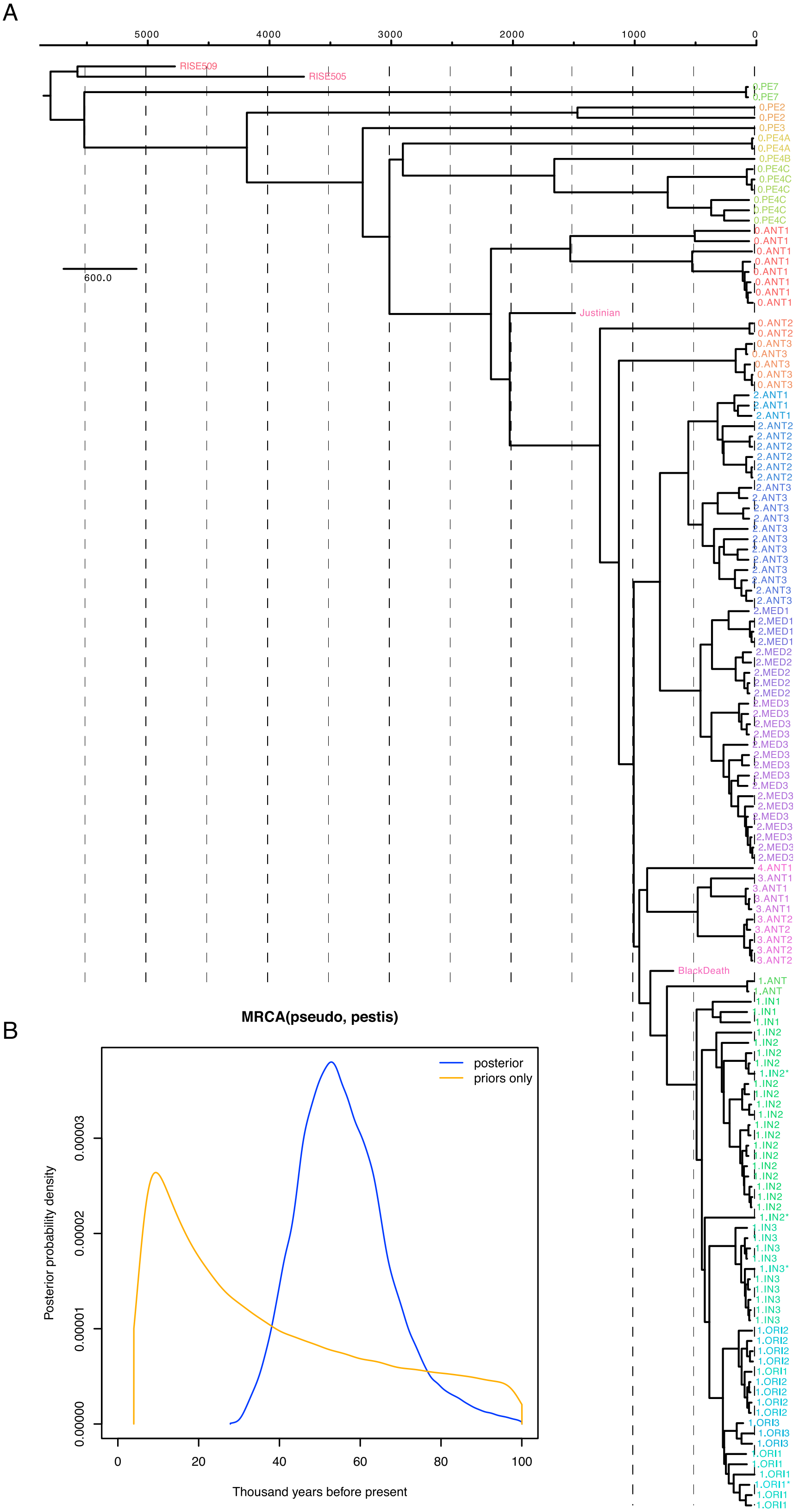

(legend on next page)

Figure S5. BEAST Divergence Dating, Related to Figures 4 and 6

(A) Maximum clade credibility tree of the *Y. pestis* clade. Strains are annotated based on their population (see Table S2) and colored according to population. Branch lengths are given as years before present. Taxa with asterisks in their name have not previously been assigned a population, but are named according to the clade they are placed in.

(B) Posterior probability density distribution for the chain where we sampled from the priors only (orange) and the chains including the alignment data (blue).

 ©2015 The Authors

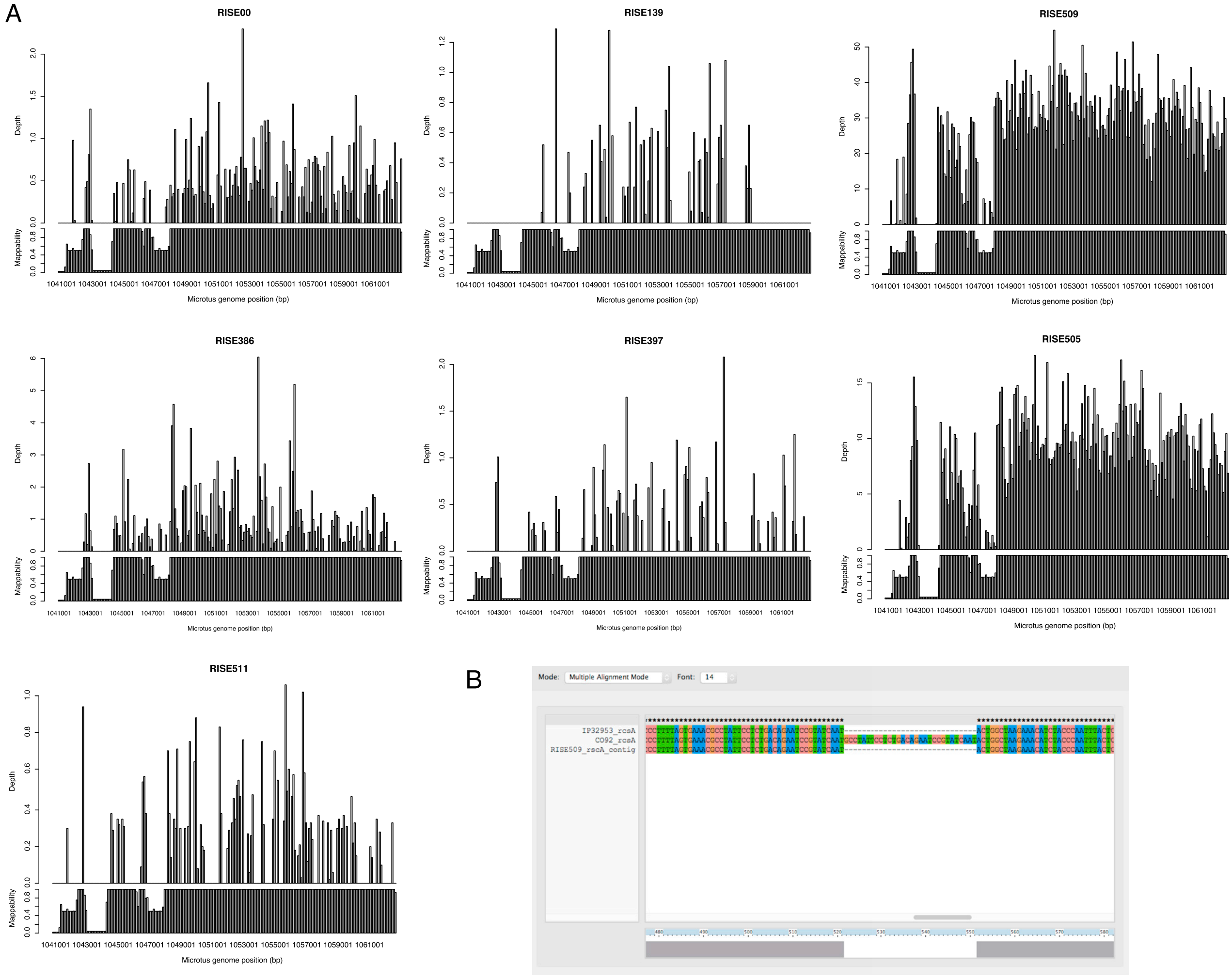

Figure S6. Investigation of Virulence Genes, Related to Figure 5

(A) Depth of coverage for the seven ancient *Y. pestis* samples in 100 bp bins across *Y. pestis Microtus* 91001 genome at 1,041 kb to 1,063 kb. For each sample the upper panel represents the depth of high quality reads in the 100 bp window. The lower panel represent mappability of the particular region calculated using GEM-mappability with a k-mer of 50.

(B) Multiple alignment of the *rcsA* gene in *Y. pseudotuberculosis* IP32953, *Y. pestis* CO92 and the contig matching the region from the RISE509 *de novo* assembly. The 30 bp internal duplication in CO92 is absent from the RISE509 sequence that therefore carries the ancestral IP32953 *rcsA* genotype.

Cell

Supplemental Information

Early Divergent Strains of *Yersinia pestis* in Eurasia 5,000 Years Ago

Simon Rasmussen, Morten Erik Allentoft, Kasper Nielsen, Ludovic Orlando, Martin Sikora, Karl-Göran Sjögren, Anders Gorm Pedersen, Mikkel Schubert, Alex Van Dam, Christian Moliin Outzen Kapel, Henrik Bjørn Nielsen, Søren Brunak, Pavel Avetisyan, Andrey Epimakhov, Mikhail Viktorovich Khalyapin, Artak Gnuni, Aivar Kriiska, Irena Lasak, Mait Metspalu, Vyacheslav Moiseyev, Andrei Gromov, Dalia Pokutta, Lehti Saag, Liivi Varul, Levon Yepiskoposyan, Thomas Sicheritz-Pontén, Robert Foley, Marta Mirazón Lahr, Rasmus Nielsen, Kristian Kristiansen, and Eske Willerslev

Supplemental Experimental Procedures

Samples and archeological sites

Short summaries of archaeological context and previous analyses performed on the sites where *Y. pesti*s was detected are provided here. Dating and stable isotope analyses on collagen are summarized in Table S1.

Sope, Estonia, Corded Ware Culture

The cemetery at Sope is situated in a coastal area in the north-eastern part of Estonia, about 1.8 km inland from the present Baltic Sea shoreline. Altogether around 10 individuals have been found. Seven individuals were unearthed during farming at the beginning of the 20th century and were later reburied (Lõugas et al., 2007). Archaeological excavations carried out in 1926 (Moora, 1932) and in 1933 (Indreko, 1935) each recovered one female skeleton – Sope I and Sope II respectively (Aul, 1935).

The deceased in Sope had been inhumed in a crouched position, which is characteristic for the Corded Ware Culture throughout the Eastern Baltic. According to osteological analysis the stature of Sope I had been 155.12 ± 3.72 cm (maximum length of the left femur 40.9 cm) and she died at the age of 25–35. The height of the second female (Sope II) was 160.56 ± 3.72 cm (maximum length of the left femur 43.1 cm) and her age at death was 22–24 years. An additional right femur was found co-mingled with Sope I belonging to an adult male whose height had been 167.72 ± 3.72 cm (maximum length of the bone 46 cm).

Only one individual was sampled for DNA from this site. The sample, RISE00, was an upper left M2, taken from the Sope I female. The female sex was confirmed through DNA sexing (Allentoft et al., 2015). She appeared to be buried in a crouched position while lying on her back with her skull towards south-east. The length of her remains was about a meter. However the skeleton was not articulated. For example the left femur was on the right side of the "body" and vice-versa, the proximal end of the left tibia was facing away from the skull and the right tibia was next to the left femur being almost parallel to it. As the skeleton was disarticulated and no metacarpals or metatarsals were found (Aul, 1935) it has been suggested that the initial decomposition of the body happened elsewhere and the skeletonized remains were gathered and wrapped inside something and then buried in the cemetery at Sope (Jonuks, 2009).

The individual was recovered with few items: an awl and a worked bone made from sheep/goat were next to the remains, and a fragment of an unidentified artifact from cattle was near the mandible, underneath which was a pig tooth (Kriiska et al., 2007).

Both females have been AMS (Accelerator Mass Spectrometry) dated (Lasak, 1996) (Table S1). δ13C on Sope I indicates protein mainly from terrestrial sources, despite the location close to the coast. δ15N is quite low, suggesting a substantial input of protein from vegetable sources.

Chociwel, Poland, Unetice culture

Chociwel is situated just a few kilometers north of Strzelin, at the foreground of the Sudetes Mountains. The site was discovered in 1993. In 1995, during construction works, part of the Únětice necropolis was excavated. In 2010, a new group of burials including three females, a young male and a child were excavated (Pokutta, 2013).

Chociwel is a multi-period site with Funnel Beaker and Globular Amphorae settlements (Cholewa, 1998), along with an Early Bronze Age (EBA) cemetery and features dated to the Migration Period and later medieval times. The EBA graves were arranged in north-south rows. The deceased were oriented E-W and only one burial resembles the north-south body orientation usual for Unetice burials.

The burials in Chociwel display a moderate number of artifacts, primarily consisting of between 2 and 5 ceramic vessels.

Four individuals were sampled for DNA from this site. The sample in which plague was detected was RISE139, from grave 20. This grave contained a skeleton osteologically determined as a female of mature age, but DNA analysis showed it to be a male (Allentoft et al., 2015).

Nine individuals have been dated (Pokutta, 2013) (Table S1). The chronometric dating is consistent with the archaeological assignment to the Unetice period, and the individual from which RISE139 was taken seems to be one of the first buried at the site. Stable isotopes have also been measured, but it is difficult to link these with the individuals sampled for DNA (Pokutta, 2013). C/N was not measured at the Uppsala lab, but in Stockholm. A high C/N value from RISE139 indicates measurement from this individual should not be trusted. The high δ15N value from grave 21/2011 is from a milk tooth and probably due to lactation

effects. Other stable isotope values from the site are within the usual range for European Neolithic/EBA populations.

Bulanovo, Russia, Sintashta culture

Cemetery, excavated by Khalyapin 2001-2002 (Khalyapin, 2001).

The dead were buried in shallow pits without barrows (Figure 1). The skeletons were laid in elongated position on their back. The inventory was not numerous (triangular stone arrowheads and bronze shape with bone-handled saws). Burial features (no mound, shallow graves, position of the dead, and lack of pottery and animal sacrifices) and the appearance of grave goods have analogies among Seima-Turbino sites. The Bulanovo cemetery can be seen as the result of interaction between the Sintashta population and the bearers of Seima-Turbino traditions.

The sample RISE386 in which plague was detected is from burial 6, individual 1. This contained bones from an adult male, 30-40 years old. DNA confirms male sex (Allentoft et al., 2015).

Three individuals were sampled from this site, two of which had good DNA preservation (Allentoft et al., 2015). All three were dated, see Table S1. The dates are in relatively good agreement, but it should be noted that RISE386 and 387 have elevated δ15N values, suggesting an intake of protein from freshwater fish, and a freshwater reservoir effect on the dates. These two dates should therefore be reduced somewhat. This is less pronounced in the third individual.

Kapan-Shahumyan, Armenia, Early Iron Age

The excavation was conducted in 2012 by Dr. Artak Gnuni near the village Shahumyan (Syunik region of Armenia). The site is located in the hills adjacent to the left bank of the river Voghji, 5 km north-east of the city of Kapan. The survey revealed the presence of a multilayer settlement and a burial ground dated to Early Iron Age. In total eleven complete burials were excavated.

Four individuals were sampled, two of which had good DNA preservation, both from burial No. 6. The tomb was a small stone chamber built of medium size stones, oriented from north to south. The upper horizon of the burial was disturbed and the stones of the ceiling were absent. The degree of the walls sloping inward implied the presence of a false vault.

Stones and clay of brown color filled the grave, with a small impurity of sand. The filling contained small fragments of ceramics, including fire exposed items and drop-shaped beads. From the north-eastern part of the burial, a fragment of a phalange was discovered. In the central part of the southern wall a carnelian bead was found. A badly burned bone fragment was also found in the central part of the tomb. The filling contained distinct patches of ash.

The burial had two skeletons. The first one (sample RISE396a) was in a crouched position on the right side and oriented from the north-east to south-west, head turned to the north. It was located on the western part of the tomb under the south wall. This individual had a massive bracelet on one hand, two more on the other, as well as a ring on the skull. There were also several ceramic items under the skeleton, two pitchers (under the skull and the pelvis), and a bowl (under the shoulder-blade). This individual had been determined as an adult female, 20-25 years old. This was confirmed by DNA sexing (Allentoft et al., 2015).

To the east of the skeleton, near the southern wall, was a stone that separated the two skeletons.

The skull of the second individual (sample RISE397a) was found in the northeast corner with a ring on the skull, similar to the first individual. The bones were in a very decayed condition, with only those of the extremities well preserved. The bones were osteologically determined as a juvenile female, 15-18 years old, but DNA analysis indicated that they actually are from a male (Allentoft et al., 2015).

A specific feature of the burials is the presence of tiny pitchers. These vessels, evidently the objects of worship, are broadly encountered artifacts in the early Iron Age monuments in Armenia. Analysis of the inventory allows the dating of the burial to the X-VIII cent. BC.

The dating of the two individuals supports this chronology (Table S1). Skeleton 1 may be slightly older, although contemporaneity is not excluded. Low $\delta 15N$ values suggest an unusually high proportion of vegetable protein, possibly including C4 plants since $\delta 13C$ is also somewhat elevated.

Kytmanovo, Russia, Andronovo culture

The Kytmanovo burial ground was excavated by A. P. Umanski in 1961-1963. Most burials were attributed to the Andronovo culture (Umanski et al., 2007). Altogether 37 graves were excavated.

The individual with plague is sample RISE505, collection number 6652-42, burial 20. It is located in the center of the Kytmanovo burial ground.

According to description and visual data there were three individuals in the grave – one adult and two children. One of the children is an infant, less than one year old. The baby was laid in front of the adult. Although the majority of the Andronovo people were buried on their left side in crouched position some small infants were buried on their right side as if they are looking at adults. This is exactly what we see in this case. The adult is probably female, 30-35 years old. Female sex was confirmed by DNA sexing (Allentoft et al., 2015). The second child according to Umanski et al., (2007) is about 4-6 years old. Regrettably we cannot check this proposition because no child bones survived.

Archeological data suggest all burials in this grave were simultaneous. This indirectly supports plague as a cause of death of these people since this is the only case in the Kytmanovo (all other burials are single or double).

The objects found in the grave are usual for Andronovo people. In this case these are three pots, one for each individual (note that the size of pots corresponds with the age of the buried). Several bronze plaques were associated with the adult. Two of them were located in the os temporalis area and one on the right shoulder. Notably, no gold artefacts were found in the grave. Taking in account the rather poor bronze kit found in the grave we can suggest that individuals from the grave did not belong to the high strata of Andronovo society. Altogether 7 burials from the 37 graves have golden objects.

Altogether five individuals from the site were sampled, all of which had good DNA preservation (Allentoft et al., 2015). All five were dated, but one person turned out to be medieval, while another dating failed, see Table S1. The samples have rather high δ13C and also somewhat elevated δ15N values which suggests protein input from C4 plants, possibly also from freshwater fish.

Afanasieva Gora (Bateni), Russia, Afanasievo culture

The Afanasieva Gora site is sometimes also called Bateni. It was excavated during the turbulent period of Russia directly after the Revolution and Civil War in 1923 by SA Tephloukhov. Although this is really a focal site on which the Afanasievo culture was recognized, no photos or regular drawings were made during excavation. A short description of the graves is given in Vadetskaya et al. 2014 (pages 124-125 and 301) (Vadetskaya et al.,

2014), based on the diary of Teploukhov. Later, graves were excavated in the 1960s by Gryaznov and these are well documented (Vadetskaya et al., 2014).

The samples RISE509 and RISE511 are both from grave 15. This is a mass grave where bones of 7 individuals were found. The skeletons were from a male (20-35), three females (two of them are 25-30 years old, one possibly older than 40) and three children (one 10-12 years, another 5-8 years old. Bones of the third were lost and no information about age exists). Osteological sexing has been confirmed by DNA (Allentoft et al., 2015).

Because single bones of roe deer, fox and chipmunk were found in the grave, Teploukhov suggested that these bones were mixed occasionally with children bones when they were reburied in the grave from some other place. Also there is one strange observation; Teploukhov remarked that incisors in the male mandible were replaced with premolars tightly placed into the alveolus. At present time most teeth have been lost.

As to artifacts the only ones were fragments of typical Afanasievo egg-shaped pots.

Collective burials are quite unusual for Afanasievo people. Most Afanasievo burials are single ones. Double burials with two adults are just 1-5% of all burials; graves with more individuals are very rare. However, in Afanasieva Gora there is one case with 4 individuals in one grave (grave 24) and and one with 7 individuals (grave 41). While this is a collective burial, in this case the archeologists believe that the burials were made successively.

Three individuals were sampled from this site (RISE509-511), two of which were from burial 15. All three are adult females, one of which is aged 20-25 (RISE509) and two 25-30 years old (RISE510 and RISE511). All three were dated, giving consistent dates, see Table S1. The interpretation of grave 15 as a mass grave is supported by the dates. Δ13C values are relatively high, which could indicate protein sources including C4 plants and/or freshwater fish.

Creation of a database for identification of *Y. pestis* reads

The database for identification of *Y. pestis* reads contained all previously sequenced *Y. pestis* strains (n=140), *Y. pseudotuberculosis* strains (n=30), *Y. similis* strains (n=5) and a selection of *Y. enterocolitica* strains (n=4) (Batzilla et al., 2011; Bos et al., 2011; Chain et al., 2006; Cui et al., 2013; Deng et al., 2002; Eppinger et al., 2007, 2009, 2010; Parkhill et al., 2001; Reuter et al., 2014; Shen et al., 2010; Song et al., 2004; Thomson et al., 2006; Wagner et al., 2014; Wang et al., 2011; Zhang et al., 2009). See Table S2 for details.

Assembly of *Y. pestis* from Justinian and Black Death samples

The Black Death plague data from samples Bos8124, Bos11972 and Bos8291 were downloaded from SRP008060 split into pairs and processed similarly to our ancient samples except that only merged sequences were used (Bos et al., 2011). Finally, the three samples were merged to one representative sample. Data from the Justinian Plague sample A120 was downloaded from SRP033879 and processed similarly to our ancient samples except that only merged and unmerged pair1 reads were used for the downstream analyses (Wagner et al., 2014).

Assembly of *Y. pestis* from modern samples

Data from modern *Y. pestis* samples were obtained by downloading reads from SRA010790 (Cui et al., 2013) and the complete genomes available at NCBI (Table S2). *Y. pseudotuberculosis* data were downloaded as reads from ERP000171 (Reuter et al., 2014). To achieve maximum comparability of data between the samples, we simulated reads from the complete genomes that were downloaded from NCBI. Using ART (Huang et al., 2012) 100 nt paired end error-free reads with an average insert size of 300 nt and depth of 50X were generated. The modern genomes were processed as the ancient samples except that they were not re-scaled for DNA-damage.

Molecular degradation patterns in *Y. pestis* and the human host

The DNA sequence length distribution obtained from shotgun sequencing data carries detailed information about the state of molecular preservation in an ancient sample (Allentoft et al., 2012). In an ancient DNA extract there should be a negative exponential correlation between the number of DNA molecules and their length. This is an effect of random fragmentation of the DNA strands, leaving few long DNA fragments and many short ones (Allentoft et al., 2012; Deagle et al., 2006). In order to validate the authenticity of the sequenced *Y. pestis* DNA we therefore examined the length distribution for all eight samples. Following previous studies (Allentoft et al., 2012; Olalde et al., 2014; Orlando et al., 2013), we investigated only the declining part of the distributions, thereby excluding biases caused by poor recovery of short DNA fragments and a fixed maximum sequencing length. The fragment length distributions for all seven *Y. pestis* datasets conformed well to an exponential decay model (R^2 = 0.94-0.99) (Figure 3 and Figure S1) as expected for ancient DNA.

Deagle et al. (Deagle et al., 2006) showed that the decay constant (λ) in the exponential relationship represents the DNA damage fraction. We estimated λ in the seven *Y. pestis*

datasets to between 0.044 and 0.139 (Figure 3, Table S3 and Figure S1), implying that only 4.4% of the phosphodiester bonds in the DNA backbone are broken in RISE392, whereas 13.9% are broken in RISE509 - the most degraded sample. Moreover, 1/ λ is equivalent to the expected average DNA fragment length (Deagle et al., 2006) and this ranged from 6.6 bp to 22.7 bp in the seven samples (Table S3). These numbers show that the *Y. pestis* DNA is highly degraded as would be expected given the age of the skeletons. We note that the average expected fragment length (1/ λ) is not equivalent to the average sequence length, which is biased both experimentally and bioinformatically.

It has been shown that long-term post mortem DNA fragmentation can be described as a rate process, and that the damage fraction (λ, per bond) can be converted to a decay rate (*k*, per bond per year), when the age of the sample is known (Allentoft et al., 2012). Using median calibrated radiocarbon ages (Table S3) we get rates of decay from 1.41E-5 to 3.17E-5 strand breaks per site per year, corresponding to molecular half-lives (for 100 bp fragments) of 492 years and 219 years respectively. After this period of time, 50% of all 100 bp stretches in the genome will be lost due to one or more strand breaks (Allentoft et al., 2012).

We also investigated the data for a correlation between DNA degradation patterns in the *Y. pestis* and that of the human host individual. In general the DNA decay proved slower for ancient human DNA than for *Y. pestis* - on average 1.6 times slower (Table S3). This is perhaps not unexpected given that *post mortem* DNA preservation conditions is likely more favorable inside human cells embedded in solid tooth cementum or dentine than they are in bacteria. Importantly, however, there was a correlation between the estimated decay rate of the human host DNA and the *Y. pestis* DNA that was co-extracted from the same individual ($R^2 = 0.55$, $P = 0.055$) (Figure 3). A fast decay rate in the human DNA is accompanied by a fast decay rate in the *Y. pestis* DNA. This apparent link constitutes another argument that the *Y. pestis* is indeed associated with the human remains rather than representing some secondary microbial invasion.

In summary, the fragmentation patterns of the DNA we have identified as *Y. pestis* carry strong signatures of authentic and highly degraded ancient DNA, which would not be expected if the DNA was derived from, for example, modern soil bacteria. Finally, it is worth noting that some of the human DNA sequence distributions display a 10 bp periodicity (Figure 3 and Figure S1). This phenomenon has been described previously in genomic data and is likely reflecting the 10 bp turn of the DNA helix combined with preferential strand cleavage of the DNA backbone facing away from nucleosome protection (Pedersen et al., 2014).

Comparison of samples to *Y. pestis* and *Y. pseudotuberculosis* reference genomes

The sequence of *Y. pestis* is very similar to that of its ancestor, *Y. pseudotuberculosis.* It was therefore important to investigate which of these species our unknown samples more closely resembled. We did this by mapping reads from the eight potential *Y. pestis* samples against both reference genomes (*Y. pestis* CO92 and *Y. pseudotuberculosis* IP32953). For each set of reads we then compared the number of reads mapping with different number of mismatches (different "edit distances") to these two references.

We first mapped several sets of reads from known *Y. pestis* and *Y. pseudotuberculosis* genomes against the two references. For comparison we also included sequences from *Y. similis,* which is an outgroup to both *Y. pestis* and *Y. pseudotuberculosis*. Typical examples of the results of mapping known sequences to the two reference genomes are shown in Figure S2. It is clearly seen that *Y. pestis* samples are slightly closer to the *Y. pestis* genome than to the Y. *pseudotuberculosis* genome: *Y. pestis* samples have more reads matching perfectly to *Y. pestis* than to *Y. pseudotuberculosis* (i.e., more reads mapping with edit distance=0; ratio > 1). The inverse is the case for *Y. pseudotuberculosis* samples, which have fewer perfect matches to *Y. pestis* than to *Y. pseudotuberculosis* (ratio < 1). Samples from *Y. similis* map about equally well to both reference genomes (ratio ~ 1), and have far fewer perfectly matching reads than the other two species (Figure S2).

Figure S2 summarizes the results of mapping several sets of reads from known species to the two reference genomes. For each edit distance, and each of the three investigated species, the distribution of frequencies obtained when mapping to the two references is shown in the form of a boxplot. The phenomena described above can bee seen to hold across many different samples, but with some spread in the actual values. Another way of investigating the closeness of sample reads to the two references, is by computing the ratio of reads mapping to *Y. pestis* vs reads mapping to *Y. pseudotuberculosis.* This is shown in Figure S2, note that the ratio is larger than 1 for perfect matches when a *Y. pestis* sample is used, and less than 1 for the other species.

Figure 3 and Figure S2 show the results of mapping the eight selected RISE samples of unknown origin against the two reference genomes. All samples, except RISE392, were found to be more similar to *Y. pestis* than to *Y. pseudotuberculosis*, and to have the majority of their reads mapping perfectly to *Y. pestis* (edit distance=0). For RISE392 reads mapped about equally well to both *Y. pestis* and *Y. pseudotuberculosis* reference genomes, and there

were fewer reads mapping perfectly (edit distance > 0) than imperfectly, indicating that RISE392 is neither *Y. pestis* nor *Y. pseudotuberculosis*, but a more distantly related species.

Bayesian classification of species assignment for unknown samples

To further quantify the qualitative assessment of read similarities described above, we constructed a naïve Bayesian classifier capable of predicting the species of an unknown sample based on the distribution of read counts mapping at different edit distances to the *Y. pestis* and *Y. pseudotuberculosis* reference genomes. Specifically, our method uses the following 10 values as input ("feature vector"): the *ratio* between reads mapped to *Y. pestis* and reads mapped to *Y. pseudotuberculosis* for edit distance 0 to 4 (these are the first 5 features), and the *frequency* of reads mapping to *Y. pestis* at edit distance 0 to 4 (the last 5 features). The output is the posterior probabilities that the sample is from *Y. pestis*, *Y. pseudotuberculosis*, or *Y. similis*. The method was trained on the data obtained from mapping reads of known origin to the two reference genomes. Details about the classifier are given below.

When the classifier was used to assess the eight unknown RISE samples, it very clearly classified all samples, except RISE392, as *Y. pestis*, with posterior probabilities of 100% (Table S3). RISE392 was found to have 0% posterior probability of being *Y. pestis*, and was instead classified as *Y. similis* (posterior probability = 100%). It should be noted that our method is only capable of classifying unknown samples as one of the three species mentioned above, and that especially samples classified as *Y. similis*, may generally correspond to any non-pestis, non-pseudotuberculosis, more distantly related species.

We also used the method to classify the remaining unknown RISE samples. The majority of these were classified as *Y. similis* (88 of 102 samples), while 13 (including the 7 investigated above) were classified as *Y. pestis* (data not shown). However, most of these samples have very few reads mapping to our *Yersinia* reference genomes, and classifications are therefore very uncertain. Among samples with more than 500 reads mapping to the reference genome, there were 20 classified as *Y. similis*, and 9 classified as *Y. pestis* (again including the 7 samples mentioned above). Table S3 shows the results also for the additional two putative *Y. pestis* samples. Among these, RISE510 was found in the same mass grave as RISE509 and RISE511 (which we are very certain are *Y. pestis*), but due to low number of reads has relatively low posterior probability of being *Y. pestis* (P = 52%).

Naïve Bayesian classifier: technical details

Naïve Bayesian classifiers use a set of input values (the feature vector) as the basis for computing the probability that an unknown data point belongs to one of a number of classes. In the present case the possible classes were the three species *Y. pestis*, *Y. pseudotuberculosis*, and *Y. similis*, and the feature vector consisted of 10 values: 5 ratios (the number of reads mapping to *Y. pestis* vs the number of reads mapping to *Y. pseudotuberculosis*, for edit distance 0 to 4), and 5 frequencies (the fraction of reads mapping to pestis at edit distance 0 to 4).

Naïve Bayesian classification is based on two main ideas: First, it is assumed that the individual features are independent, conditional on the class, even though this is often incorrect (hence "naïve"). It has been shown that despite this overly simplified assumption, naïve Bayesian classification often has very good performance in classification (Hand and Yu, 2001; Zhang, 2004). The assumption of independence means that it is possible to compute the joint probability of observing any set of feature values, given the class, simply by multiplying the probabilities of observing the individual features, given that class:

$$P(F_1, F_2, \dots, F_{10}|C_1) = P(F_1|C_1)P(F_2|C_1) \dots P(F_{10}|C_1) = \prod_{i=1}^{10} P(F_i|C_1)$$

This quantity (the probability of the observed feature values, given the class) is referred to as the "likelihood". How the individual probabilities are computed depends on the hypotheses about the investigated system. In our case we assume that each of the 10 features has a typical range of values specific to each class (for instance, the ratio for edit=0 is > 1 for pestis, and <1 for the other two species). Specifically, we assume that any given feature value is drawn from a normal distribution with mean and standard deviation depending on the class. The probability density for a given feature value for a given class is therefore found as the normal probability density using the mean and standard deviation for that feature and class. As an example, the probability density of observing the read mapping ratio 1.3 for edit distance = 0 for the class *Y. pestis*, is the following in our model:

$$P(F_1 = 1.3|pestis) = f_{normal}(x = 1.3|\mu = 1.25, \sigma = 0.057) = 4.76$$

The means and standard deviations are parameters in our model, and can be estimated simply by computing means and standard deviations from known examples ("training data" – in our case the data used also in Figure S2B-C these are maximum likelihood estimates of the parameters). Note that the independence assumption also means that it is possible to estimate parameters in the model from much smaller data sets than if features were not taken to be

independent (one just needs sufficient training examples to estimate parameters for each feature individually, instead of examples from all possible combinations of all features).

The second main idea in naïve Bayesian classification is to use Bayes theorem to compute the posterior probability of the possible classes, given the observed feature vector. As an example, the posterior probability for class 1 is computed as follows:

$$P(C_1|\boldsymbol{F}) = \frac{P(\boldsymbol{F}|C_1)P(C_1)}{P(\boldsymbol{F})}$$

Here, $\boldsymbol{F}$ is the entire feature vector (containing 10 values in our case) and the likelihood $P(\boldsymbol{F}|C_1)$ is calculated assuming independence of features as shown above. $P(C_1)$ is known as the prior probability of the class. In the present case we simply used a flat prior distribution, with the same prior probability for all three classes. $P(C_1|\boldsymbol{F})$ is the posterior probability of the class, and quantifies our degree of belief in this class after seeing the data. Finally, $P(\boldsymbol{F})$ is known as the "evidence" and can be seen as a normalizing factor, ensuring that the posterior class probabilities will sum to one. $P(\boldsymbol{F})$ is computed as the sum of the probabilities for the three possible ways of getting the observed features:

$$P(\boldsymbol{F}) = P(\boldsymbol{F}|C_1)P(C_1) + P(\boldsymbol{F}|C_2)P(C_2) + P(\boldsymbol{F}|C_3)P(C_3)$$

As mentioned, we estimated means and standard deviations for each of the 10 features, for each of the 3 classes, from a set of known samples mapped against the *Y. pestis* and *Y. pseudotuberculosis* reference genomes. It turned out that the data available to estimate parameters for *Y. similis* displayed what we judged to be unrealistically little diversity, and we therefore estimated the standard deviations for this class by taking the average of the corresponding standard deviations estimated for *Y. pestis* and *Y. pseudotuberculosis*. (This approach, where parameter values from other groups are used to help regularize the estimate for a group with limited data, is known as shrinkage).

Analysis of sequencing depth, expected coverage, and actual coverage

Sequencing reads are not distributed evenly across a sequenced genome - some positions are covered by more than the average number of reads and others by less. Consequently, coverage (the fraction of positions covered by at least one read) is not necessarily 100% even when the sequencing depth (the average number of reads covering any given position) is well above 1. It is possible to compute the expected coverage based on the distribution of read lengths, under the assumption that read locations have been drawn randomly from the entire

genome (see below). We here use the comparison of actual and expected coverage computed in this manner, as yet another way to assess the authenticity of the analyzed reads. The idea is that if mapped reads do in fact originate from *Y. pestis*, then their locations will be close to randomly distributed across the reference genome, and expected coverage should therefore match actual coverage well. If, on the other hand, the reads do not belong to *Y. pestis*, then their mapped locations on the reference genome are more likely to be biased, for instance with over-representation in regions of low complexity, or perhaps in regions that have been more highly conserved through evolution. In that case, the match between actual and expected coverage should be worse.

Assuming that all reads have exactly the same length the expected coverage can be computed using the following expression: $c = 1 - \left(1 - \frac{l}{g}\right)^r$, where l=read length, g=genome length, and r=number of reads. The rationale is as follows: The probability that any given position in the reference genome will be covered by a read is $\frac{l}{g}$. The probability a position will *not* be covered by a single read is therefore $1 - \frac{l}{g}$. The probability that any given position will *not* be covered after r reads have been placed randomly and independently is therefore $(1 - \frac{l}{g})^r$. The probability that a given read *is* in fact covered after placing r reads, is 1 minus the probability that it is not covered, i.e., $1 - \left(1 - \frac{l}{g}\right)^r$. Since the expected fraction of covered sites, is the same as the probability that any given site is covered, this will also be the expected coverage, c.

Based on the expression above, it is fairly simple to compute the expected coverage also in the event that all reads do not have the same length. If, for instance there are r_l reads of length l_1, and r_2 reads of length l_2, then the expected coverage is simply: $c = 1 - \left(1 - \frac{l_1}{g}\right)^{r_1}\left(1 - \frac{l_2}{g}\right)^{r_2}$. More generally, if the reads have N different lengths, l_1 to l_N, with counts r_l to r_N, then the expected coverage is:

$$c = 1 - \prod_{i=1}^{N}\left(1 - \frac{l_i}{g}\right)^{r_i}$$

Even if the location of reads are in fact randomly sampled from the reference genome, there are still two major reasons why an expected coverage, computed according to this equation, may not correspond to the actual coverage. First, if the reference genome contains repeats with a length longer than the read length, then it will not be possible to uniquely map reads corresponding to these repeats. The expected coverage will therefore only refer to the

mappable part of the reference sequence. For each reference sequence (the *Y. pestis* genome and the three associated plasmids), we, for each sample, determined the mappable fraction using k-mer lengths similar to the average read lengths in that sample. Specifically, we determined the mappable fraction for each reference sequence using kmers of length 40, 50, and 60, and then used the mappability value with the k-mer length closest to the actual average read length for each sample/reference combination. The expected coverage, accounting for mappability, is then computed by multiplying the expected coverage by the fraction of the reference sequence that is mappable: $c_{map} = f_{map}c$. The second reason why expected coverage may differ from actual coverage, is if the reference sequence contains regions that are not present in the sequenced sample. We found this to be the case for the pMT1 plasmid, which, for 6 of the investigated samples compared to the reference sequence, was found to lack a 19 kb region harboring the *ymt* gene important for pathogenicity. Again, this can be accounted for by multiplying the expected coverage by the fraction of the reference sequence that is present: $c_{map,del} = f_{del}f_{map}c$. In the case of pMT1, samples lacking this 19 kb region were clearly seen in plots of expected vs actual coverage as being placed well below the line corresponding to perfect correlation.

Figure 3 shows plots of actual vs. expected coverage computed for all samples for the chromosome and the plasmid sequences, using the equations above (and thus accouting for mappability and for the lacking region in some pMT1). It can be seen that expected coverage computed for the reads corresponding to assumed *Y. pestis* fit very well to the actually observed values. The majority of reads not assumed to be *Y. pestis* have very low read counts mapping to the reference sequences, and are seen as a cloud of points in the lower left corners of the plots. A few samples can be seen to have a high count of reads mapped to the *Y. pestis* reference chromosome, and therefore also high expected coverage, but much lower actual coverage, and are therefore most likely not *Y. pestis*. Included among these is the sample RISE392 (shown as red dots in the plots), which was also deemed not to be *Y. pestis* based on the distribution of edit distances.

Genotyping for phylogenetic analyses

The calls were generated from alignments versus *Y. pseudotuberculosis* IP32953 using samtools-0.1.18 and bcftools-0.1.17 (Li et al., 2009). The genotype calls were filtered by removing heterozygote variants, indels and variants that clustered within 10bp of each other, as well as variants within 10 bp of a gap. Additionally genotype calls in modern samples were required to have at least 10 high quality base calls (given by DP4) and ancient samples to have at least 4 high quality base calls per site. To create full-length consensus sequences for

each sample the missing sites in the VCF files were then filled with N basecalls and converted to fasta.

Heterozygosity estimates

To estimate if the RISE505 or RISE509 strains represented an infection with two different *Y. pestis* strains we determined the number of heterozygote sites in the genomes of RISE505, RISE509, the Black Death strain (Bos et al., 2011) and the strains from Cui et al. (Cui et al., 2013). The rationale for this is that heterozygote genotype calls for haploid organisms are normally caused by mapping errors, but in the case of a mixed infection will be caused by divergence between the strains. To allow for comparison between the samples we sampled all the bam-files to the same average depth as RISE505 (8.7X) and RISE509 (29.4X) using samtools (Li et al., 2009). We excluded the Justinian strain (Wagner et al., 2014) from the analysis due to the low average depth across the chromosome (4.3X). Hereafter, we genotyped each of the individuals based on the *Y. pestis* CO92 chromosome and extracted heterozygote genotype calls with a depth equal to or larger than 10 (base quality >= 13). We removed all transitions, as these are typically patterns of DNA damage, and only kept transversions.

Analysis of virulence associated genes

The 55 genes (Black et al., 2000; Blaylock et al., 2010; Burghout et al., 2004; Bzymek et al., 2012; Cheng and Schneewind, 2000; Cornelis, 2002; Day and Plano, 2000; Day et al., 2000; Diepold et al., 2011; Du et al., 2002; Felek et al., 2010; Fields et al., 1999; Fowler et al., 2009; Haddix and Straley, 1992; Håkansson et al., 1996; Hinnebusch et al., 1996, 2002; Huang and Lindler, 2004; Iriarte and Cornelis, 1999; Juris et al., 2000; Kerschen et al., 2004; Li et al., 2014; Lindler et al., 1990; Mukherjee et al., 2006; Payne and Straley, 1998; Perry and Fetherston, 1997; Plano et al., 1991; Ramamurthi and Schneewind, 2003; Rosqvist et al., 1994; Rouvroit et al., 1992; Silva-Herzog et al., 2008; Sodeinde et al., 1992; Stainier et al., 2000; Williams and Straley, 1998; Woestyn et al., 1994) that we identified as associated with virulence of *Y. pestis* are shown in Figure 5 as well as listed in Table S6. For identification of the DFR4 region we used the location of 1,041kb to 1,063kb in the *Y. pestis microtus* 91001 genome. The mappability of the DFR4 region was calculated using GEM-mappability library (Derrien et al., 2012) with a k-mer of 50 using the entire genome as input.

Genotyping of *pde2*, *pde3* and *rcsA* involved in survival in flea gut

We investigated the loss of function mutations in three genes (*pde2*, *pde3* and *rcsA*) which lead to an *Y. pestis* phenotype that causes blockage of the flea gut and thereby increased

probability of transmission (Sun et al., 2014). The loss of function mutations for the genes are a frameshift mutation (6As -> 7As) in the *pde2* gene, a C->T mutation in the promoter and a nonsense point mutation in the *pde3* gene, and a 30bp internal duplication in the *rcsA* gene.

For *pde2* we used the genotypes of RISE509 that were called using the *Y. pseudotuberculosis* IP32953 genome and we did not find any evidence for an insertion which is in concordance with the 6A genotype (position 1,560,134). Likewise when investigating the genotypes based on the *Y. pestis* CO92 genome we find a deletion corresponding to the 6A genotype (position 1,434,043). For the RISE505 sample the *pde2* positions had low coverage (1-2 reads only) and we were unable to determine the genotype.

For *pde3* we investigated both the promoter mutation (IP32953: C -> T at 3,944,166) and the nonsense mutation (IP32953: G -> A at 3,944,534) in RISE509. Although the promoter mutation is a C-T mutation and therefore likely to be confounded by DNA damage, we found 6 non-damaged (not rescaled by MapDamage2) high quality bases confirming the mutation. Likewise, the G-A nonsense mutation is also likely to be masked by DNA damage, but we identified 62 reads in support of G versus only one read in support of A. Likewise as for *pde2*, the RISE505 sample had low read support but still supported the same genotypes as identified in RISE509.

Because *rcsA* is a 30bp internal duplication we performed *de novo* assembly of the RISE509 data using SPAdes as described above. We identified the contig spanning the region and performed multiple alignment using ClustalX (Larkin et al., 2007) (Figure S6). The *de novo* assembled contig did not have the internal duplication and RISE509 therefore has the ancestral form of *rcsA*.

Genotyping *pla* mutations

We identified a novel non-synonymous C to G mutation in amino acid 31 (amino acid 51 in the CO92 reference sequence, position 6,815 on pPCP1) replacing an isoleucine with a valine. We found the mutation to be supported by 55 reads in RISE505 (1746-1626 cal BC) and 46 reads in RISE509 (2815-2677 cal BC), respectively. All other *Y. pestis* genomes, including RISE397 (1048-885 cal BC) carried the derived isoleucine allele (supported by 7 reads).

We additionally investigated the isoleucine 259 to threonine mutation (279 in the CO92 reference sequence, position 7,500 on pPCP1) (Zimbler et al., 2015). However, because the genotype of CO92 at this position is a C and the ancestral state is a T, the genotyping can be confounded by ancient DNA damage. For each of the RISE397, RISE505 and RISE509

samples, the non-damaged bases (not rescaled by MapDamage2) at this site were all supporting the ancestral allele (T) with 3, 2 and 2 reads respectively. We additionally called genotypes for RISE397, RISE505 and RISE509 based on the *Y. pestis microtus* 91001 *pla* gene which contains the ancestral T allele. Here the ancestral allele was supported by 11, 19 and 6 reads, respectively.

Genotyping the *flhD* gene

All *Y. pestis* strains sequenced prior to this study have an insertion of a T in the *flhD* gene (CO92 position: 1,892,659) that is a regulatory gene involved in flagella synthesis (Minnich and Rohde, 2007). When investigating RISE505 and RISE509 for this insertion, we found them to harbor the ancestral and functional *flhD* allele supported by 16 and 29 high quality bases, respectively. The downstream deleted base (in the CO92 genome) was not supported by any high quality reads in any of the two samples.

Supplemental References

Aul, J. (1935). Étude anthropologique des ossements humains néolithiques de Sope. Õpetatud Eesti Seltsi Aastaraam. 1933 224–282.

Batzilla, J., Höper, D., Antonenka, U., Heesemann, J., and Rakin, A. (2011). Complete genome sequence of Yersinia enterocolitica subsp. palearctica serogroup O:3. J. Bacteriol. *193*, 2067.

Black, D.S., Marie-Cardine, A., Schraven, B., and Bliska, J.B. (2000). The Yersinia tyrosine phosphatase YopH targets a novel adhesion-regulated signalling complex in macrophages. Cell. Microbiol. *2*, 401–414.

Blaylock, B., Berube, B.J., and Schneewind, O. (2010). YopR impacts type III needle polymerization in Yersinia species. Mol. Microbiol. *75*, 221–229.

Burghout, P., Beckers, F., de Wit, E., van Boxtel, R., Cornelis, G.R., Tommassen, J., and Koster, M. (2004). Role of the pilot protein YscW in the biogenesis of the YscC secretin in Yersinia enterocolitica. J. Bacteriol. *186*, 5366–5375.

Bzymek, K.P., Hamaoka, B.Y., and Ghosh, P. (2012). Two translation products of Yersinia yscQ assemble to form a complex essential to type III secretion. Biochemistry *51*, 1669–1677.

Chain, P.S.G., Hu, P., Malfatti, S.A., Radnedge, L., Larimer, F., Vergez, L.M., Worsham, P., Chu, M.C., and Andersen, G.L. (2006). Complete genome sequence of Yersinia pestis strains Antiqua and Nepal516: evidence of gene reduction in an emerging pathogen. J. Bacteriol. *188*, 4453–4463.

Cheng, L.W., and Schneewind, O. (2000). Yersinia enterocolitica TyeA, an intracellular regulator of the type III machinery, is required for specific targeting of YopE, YopH, YopM, and YopN into the cytosol of eukaryotic cells. J. Bacteriol. *182*, 3183–3190.

Cholewa, P. (1998). Osady neolityczne na stanowisku nr 1 w Chociwelu, gm: Strzelin. Neolithic settlements in site 1 in Chociwel, near Strzelin. Wrocław Wydaw. Uniw. Wrocławskiego. *30*, 81–168.

Cornelis, G.R. (2002). The Yersinia Ysc-Yop “type III” weaponry. Nat. Rev. Mol. Cell Biol. *3*, 742–752.

Day, J.B., and Plano, G. V (2000). The Yersinia pestis YscY protein directly binds YscX, a secreted component of the type III secretion machinery. J. Bacteriol. *182*, 1834–1843.

Day, J.B., Guller, I., and Plano, G. V (2000). Yersinia pestis YscG protein is a Syc-like chaperone that directly binds yscE. Infect. Immun. *68*, 6466–6471.

Deagle, B.E., Eveson, J.P., and Jarman, S.N. (2006). Quantification of damage in DNA recovered from highly degraded samples--a case study on DNA in faeces. Front. Zool. *3*, 11.

Deng, W., Burland, V., Plunkett, G., Boutin, A., Mayhew, G.F., Liss, P., Perna, N.T., Rose, D.J., Mau, B., Zhou, S., et al. (2002). Genome Sequence of Yersinia pestis KIM. J. Bacteriol. *184*, 4601–4611.

Derrien, T., Estellé, J., Marco Sola, S., Knowles, D.G., Raineri, E., Guigó, R., and Ribeca, P. (2012). Fast computation and applications of genome mappability. PLoS One *7*, e30377.

Diepold, A., Wiesand, U., and Cornelis, G.R. (2011). The assembly of the export apparatus (YscR,S,T,U,V) of the Yersinia type III secretion apparatus occurs independently of other structural components and involves the formation of an YscV oligomer. Mol. Microbiol. *82*, 502–514.

Du, Y., Rosqvist, R., and Forsberg, A. (2002). Role of fraction 1 antigen of Yersinia pestis in inhibition of phagocytosis. Infect. Immun. *70*, 1453–1460.

Eppinger, M., Rosovitz, M.J., Fricke, W.F., Rasko, D.A., Kokorina, G., Fayolle, C., Lindler, L.E., Carniel, E., and Ravel, J. (2007). The complete genome sequence of Yersinia pseudotuberculosis IP31758, the causative agent of Far East scarlet-like fever. PLoS Genet. *3*, e142.

Eppinger, M., Guo, Z., Sebastian, Y., Song, Y., Lindler, L.E., Yang, R., and Ravel, J. (2009). Draft genome sequences of Yersinia pestis isolates from natural foci of endemic plague in China. J. Bacteriol. *191*, 7628–7629.

Eppinger, M., Worsham, P.L., Nikolich, M.P., Riley, D.R., Sebastian, Y., Mou, S., Achtman, M., Lindler, L.E., and Ravel, J. (2010). Genome sequence of the deep-rooted Yersinia pestis strain Angola reveals new insights into the evolution and pangenome of the plague bacterium. J. Bacteriol. *192*, 1685–1699.

Felek, S., Muszyński, A., Carlson, R.W., Tsang, T.M., Hinnebusch, B.J., and Krukonis, E.S. (2010). Phosphoglucomutase of Yersinia pestis is required for autoaggregation and polymyxin B resistance. Infect. Immun. *78*, 1163–1175.

Fields, K.A., Nilles, M.L., Cowan, C., and Straley, S.C. (1999). Virulence Role of V Antigen of Yersinia pestis at the Bacterial Surface. Infect. Immun. *67*, 5395–5408.

Fowler, J.M., Wulff, C.R., Straley, S.C., and Brubaker, R.R. (2009). Growth of calcium-blind mutants of Yersinia pestis at 37 degrees C in permissive Ca2+-deficient environments. Microbiology *155*, 2509–2521.

Haddix, P.L., and Straley, S.C. (1992). Structure and regulation of the Yersinia pestis yscBCDEF operon. J. Bacteriol. *174*, 4820–4828.

Håkansson, S., Schesser, K., Persson, C., Galyov, E.E., Rosqvist, R., Homblé, F., and Wolf-Watz, H. (1996). The YopB protein of Yersinia pseudotuberculosis is essential for the translocation of Yop effector proteins across the target cell plasma membrane and displays a contact-dependent membrane disrupting activity. EMBO J. *15*, 5812–5823.

Hand, D.J., and Yu, K. (2001). Idiot's Bayes? Not So Stupid After All? Int. Stat. Rev. *69*, 385–398.

Hinnebusch, B.J., Perry, R.D., and Schwan, T.G. (1996). Role of the Yersinia pestis hemin storage (hms) locus in the transmission of plague by fleas. Science *273*, 367–370.

Huang, X.-Z., and Lindler, L.E. (2004). The pH 6 antigen is an antiphagocytic factor produced by Yersinia pestis independent of Yersinia outer proteins and capsule antigen. Infect. Immun. *72*, 7212–7219.

Huang, W., Li, L., Myers, J.R., and Marth, G.T. (2012). ART: a next-generation sequencing read simulator. Bioinformatics *28*, 593–594.

Indreko, R. (1935). Sépultures néolithiques en Estonie. Õpetatud Eesti Seltsi Aastaraam. 1933 202–223.

Iriarte, M., and Cornelis, G.R. (1999). Identification of SycN, YscX, and YscY, three new elements of the Yersinia yop virulon. J. Bacteriol. *181*, 675–680.

Jonuks, T. (2009). Eesti muinasusund. Dissertationes archaeologiae universitatis Tartuensis 2. Tartu.

Juris, S.J., Rudolph, A.E., Huddler, D., Orth, K., and Dixon, J.E. (2000). A distinctive role for the Yersinia protein kinase: actin binding, kinase activation, and cytoskeleton disruption. Proc. Natl. Acad. Sci. U. S. A. *97*, 9431–9436.

Kerschen, E.J., Cohen, D.A., Kaplan, A.M., and Straley, S.C. (2004). The plague virulence protein YopM targets the innate immune response by causing a global depletion of NK cells. Infect. Immun. *72*, 4589–4602.

Khalyapin, M. V. (2001). The first cemetery of the Sintashta Culture. In The Bronze Age in Eastern Europe: Characteristics of Cultures, the Chronology and Periodization, Y.I. Kolev, ed. (Samara: NTTZ), pp. 417–425.

Kriiska, A., Lõugas, L., Lõhmus, M., Mannermaa, K., and Johanson, K. (2007). New AMS dates from Estonian Stone Age burial sites. Est. J. Archaeol. *11*, 83–121.

Larkin, M.A., Blackshields, G., Brown, N.P., Chenna, R., McGettigan, P.A., McWilliam, H., Valentin, F., Wallace, I.M., Wilm, A., Lopez, R., et al. (2007). Clustal W and Clustal X version 2.0. Bioinformatics *23*, 2947–2948.

Lasak, I. (1996). Obiekty kultury unietyckiej z Chociwela, woj. wrocławskie. Unětice culture objects from Chociwel, province of Wrocław. Archeologiczne *37*.

Li, Y., Li, L., Huang, L., Francis, M.S., Hu, Y., and Chen, S. (2014). Yersinia Ysc-Yop type III secretion feedback inhibition is relieved through YscV-dependent recognition and secretion of LcrQ. Mol. Microbiol. *91*, 494–507.

Lindler, L.E., Klempner, M.S., and Straley, S.C. (1990). Yersinia pestis pH 6 antigen: genetic, biochemical, and virulence characterization of a protein involved in the pathogenesis of bubonic plague. Infect. Immun. *58*, 2569–2577.

Lõugas, L., Kriiska, A., and Maldre, L. (2007). New dates for the Late Neolithic Corded Ware Culture burials and early husbandry in the East Baltic region. Archaeofauna *16*, 21–31.

Moora, H. (1932). Die Vorzeit Estlands (Tartu).

Mukherjee, S., Keitany, G., Li, Y., Wang, Y., Ball, H.L., Goldsmith, E.J., and Orth, K. (2006). Yersinia YopJ acetylates and inhibits kinase activation by blocking phosphorylation. Science *312*, 1211–1214.

Olalde, I., Allentoft, M.E., Sánchez-Quinto, F., Santpere, G., Chiang, C.W.K., DeGiorgio, M., Prado-Martinez, J., Rodríguez, J.A., Rasmussen, S., Quilez, J., et al. (2014). Derived immune

and ancestral pigmentation alleles in a 7,000-year-old Mesolithic European. Nature *507*, 225–228.

Orlando, L., Ginolhac, A., Zhang, G., Froese, D., Albrechtsen, A., Stiller, M., Schubert, M., Cappellini, E., Petersen, B., Moltke, I., et al. (2013). Recalibrating Equus evolution using the genome sequence of an early Middle Pleistocene horse. Nature *499*, 74–78.

Payne, P.L., and Straley, S.C. (1998). YscO of Yersinia pestis is a mobile core component of the Yop secretion system. J. Bacteriol. *180*, 3882–3890.

Pedersen, J.S., Valen, E., Velazquez, A.M.V., Parker, B.J., Rasmussen, M., Lindgreen, S., Lilje, B., Tobin, D.J., Kelly, T.K., Vang, S., et al. (2014). Genome-wide nucleosome map and cytosine methylation levels of an ancient human genome. Genome Res. *24*, 454–466.

Plano, G. V, Barve, S.S., and Straley, S.C. (1991). LcrD, a membrane-bound regulator of the Yersinia pestis low-calcium response. J. Bacteriol. *173*, 7293–7303.

Pokutta, D.A. (2013). Population Dynamics, Diet and Migrations of the Unetice Culture in Poland. University of Gothenburg.

Ramamurthi, K.S., and Schneewind, O. (2003). Yersinia yopQ mRNA encodes a bipartite type III secretion signal in the first 15 codons. Mol. Microbiol. *50*, 1189–1198.

Reuter, S., Connor, T.R., Barquist, L., Walker, D., Feltwell, T., Harris, S.R., Fookes, M., Hall, M.E., Petty, N.K., Fuchs, T.M., et al. (2014). Parallel independent evolution of pathogenicity within the genus Yersinia. Proc. Natl. Acad. Sci. U. S. A. *111*, 6768–6773.

Rosqvist, R., Magnusson, K.E., and Wolf-Watz, H. (1994). Target cell contact triggers expression and polarized transfer of Yersinia YopE cytotoxin into mammalian cells. EMBO J. *13*, 964–972.

Rouvroit, C., Sluiters, C., and Cornelis, G. (1992). Role of the transcriptional activator, VirF, and temperature in the expression of the pYV plasmid genes of Yersinia enterocolitica. Mol. Microbiol. *6*, 395–409.

Shen, X., Wang, Q., Xia, L., Zhu, X., Zhang, Z., Liang, Y., Cai, H., Zhang, E., Wei, J., Chen, C., et al. (2010). Complete genome sequences of Yersinia pestis from natural foci in China. J. Bacteriol. *192*, 3551–3552.

Silva-Herzog, E., Ferracci, F., Jackson, M.W., Joseph, S.S., and Plano, G. V (2008). Membrane localization and topology of the Yersinia pestis YscJ lipoprotein. Microbiology *154*, 593–607.

Song, Y., Tong, Z., Wang, J., Wang, L., Guo, Z., Han, Y., Zhang, J., Pei, D., Zhou, D., Qin, H., et al. (2004). Complete genome sequence of Yersinia pestis strain 91001, an isolate avirulent to humans. DNA Res. *11*, 179–197.

Stainier, I., Bleves, S., Josenhans, C., Karmani, L., Kerbourch, C., Lambermont, I., Tötemeyer, S., Boyd, A., and Cornelis, G.R. (2000). YscP, a Yersinia protein required for Yop secretion that is surface exposed, and released in low Ca2+. Mol. Microbiol. *37*, 1005–1018.

Thomson, N.R., Howard, S., Wren, B.W., Holden, M.T.G., Crossman, L., Challis, G.L., Churcher, C., Mungall, K., Brooks, K., Chillingworth, T., et al. (2006). The Complete Genome Sequence and Comparative Genome Analysis of the High Pathogenicity Yersinia enterocolitica Strain 8081. PLoS Genet. *2*, e206.

Umanski, A., Kiryushin, Y., and Grushin, S. (2007). The burial traditions of the Andronovo people of Chumysh area (based on Kytmanovo data) (Barnaul: Altai University Press).

Vadetskaya, E., Polyakov, A., and Stepanova, N. (2014). The set sites of the Afanasievo culture (Barnaul: Azbuka).

Wang, X., Li, Y., Jing, H., Ren, Y., Zhou, Z., Wang, S., Kan, B., Xu, J., and Wang, L. (2011). Complete genome sequence of a Yersinia enterocolitica "Old World" (3/O:9) strain and comparison with the "New World" (1B/O:8) strain. J. Clin. Microbiol. *49*, 1251–1259.

Williams, A.W., and Straley, S.C. (1998). YopD of Yersinia pestis Plays a Role in Negative Regulation of the Low-Calcium Response in Addition to Its Role in Translocation of Yops. J. Bacteriol. *180*, 350–358.

Woestyn, S., Allaoui, A., Wattiau, P., and Cornelis, G.R. (1994). YscN, the putative energizer of the Yersinia Yop secretion machinery. J. Bacteriol. *176*, 1561–1569.

Zhang, H. (2004). The optimality of naive Bayes. AA *1*, 3.

Zhang, Z., Hai, R., Song, Z., Xia, L., Liang, Y., Cai, H., Shen, X., Zhang, E., Xu, J., Yu, D., et al. (2009). Spatial Variation of Yersinia pestis from Yunnan Province of China. Am. J. Trop. Med. Hyg. *81*, 714–717.
